# Dendrimers Functionalized with Palladium Complexes of N-, N,N-, and N,N,N-Ligands

**DOI:** 10.3390/molecules26082333

**Published:** 2021-04-17

**Authors:** Quentin Vanbellingen, Paul Servin, Anaïs Coinaud, Sonia Mallet-Ladeira, Régis Laurent, Anne-Marie Caminade

**Affiliations:** 1 Laboratoire de Chimie de Coordination du CNRS, 205 Route de Narbonne, BP 44099, CEDEX 4, 31077 Toulouse, France; quentin.vanbellingen@servier.com (Q.V.); paul.servin@xeroscleaning.com (P.S.); amors.anais@gmail.com (A.C.); sonia.ladeira@lcc-toulouse.fr (S.M.-L.); regis.laurent@lcc-toulouse.fr (R.L.); 2 LCC-CNRS, Université de Toulouse, CNRS, CEDEX 4, 31077 Toulouse, France; 3 Technologie Servier—Center of Excellence in Drug Safety and Pharmacokinetics 25/27 rue Eugène Vignat, CS 11749, CEDEX 1, 45007 Orléans, France

**Keywords:** dendrimer, ligands, imine, palladium, complexes, phenol, X-ray diffraction, catalysis

## Abstract

Pyridine, pyridine imine, and bipyridine imine ligands functionalized by a phenol have been synthesized and characterized, in many cases by X-ray diffraction. Several of these N-, N,N-, and N,N,N,-ligands have been grafted onto the surface of phosphorhydrazone dendrimers, from generation 1 to generation 3. The complexation ability of these monomers and dendrimers towards palladium(II) has been assayed. The corresponding complexes have been either isolated or prepared in situ. In both cases, the monomeric and dendritic complexes have been tested as catalysts in Heck couplings and in Sonogashira couplings. In some cases, a positive dendritic effect has been observed, that is, an increase of the catalytic efficiency proportional to the dendrimer generation.

## 1. Introduction

Dendrimers are hyperbranched nanomolecules, also called molecular trees, which pertain to the “nanoworld” by virtue of their size, but which differ fundamentally from “hard” nanoparticles constituted of metals. Dendrimers are most generally synthesized by a divergent process, starting from a multifunctional core to which are attached progressively several layers of branching units. Each sequence of reactions increases the number of terminal functions and provides a new “generation” of the dendrimers. Different types of dendrimers are known, which differ by the nature of their core and of their branches. Among all types of dendrimers [1], those possessing main group elements as branching units have often exceptional properties [2], which in some cases are not attainable with purely organic dendrimers [3]. Two principal types of dendrimers comprising main group elements in their structure are known: carbosilane dendrimers [4], and phosphorhydrazone dendrimers [5]. One of the main advantages of the latter is their easy characterization by ^31^P NMR. Indeed, this technique allows monitoring each step of the synthetic process [6] and is powerful to ascertain the completion of the reactions that occur on the surface of the dendrimers [7].

Phosphorhydrazone dendrimers have outstanding properties in many different fields, related to catalysis, nanomaterials, and also biology/nanomedicine [8]. They have been used successfully as soluble supports of catalytic entities, allowing for instance the use of less expensive metals such as copper [9]. Dendrimer complexes have been recovered and reused many times [10], and even the possibility to switch ON/OFF the catalytic efficiency has been proven [11].

**Figure 1:**
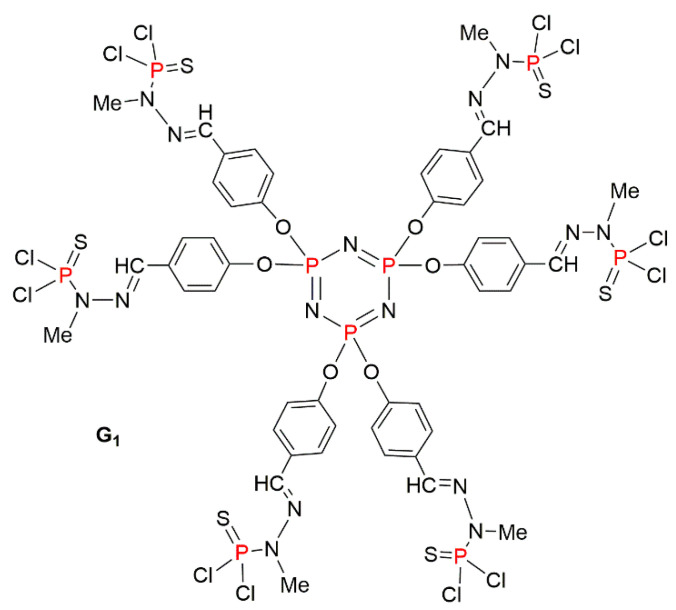
Full structure of the first generation of phosphorhydrazone dendrimer.

In this paper, we report the synthesis and characterization (often by X-ray crystal structure diffraction) of ligands incorporating one, two, or three nitrogen atoms in their structure. These ligands are functionalized in all cases by a phenol to enable their grafting to the P(S)Cl_2_ terminal functions of phosphorhydrazone dendrimers (see the first generation of these dendrimers, **G_1_**, in Figure 1). We report also in this paper that the palladium complexes of these dendrimers are useful catalysts in C-C cross-coupling reactions, in particular of Heck [12] and Sonogashira [13,14] types.

## 2. Results

### 2.1. Synthesis and Characterization of Functionalized Ligands

**Figure 2:**
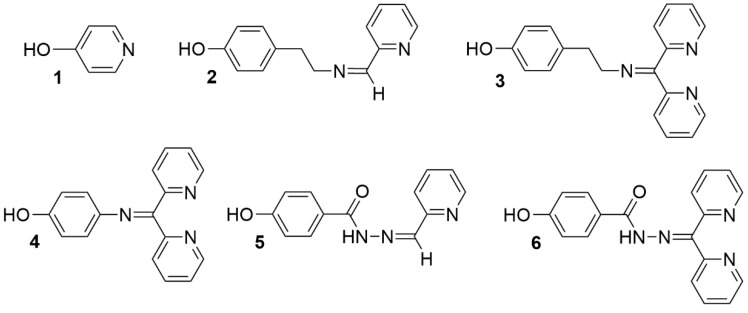
N-, N,N- and N,N,N-ligands functionalized by a phenol.

With the exception of 4-hydroxypyridine **1**, which is commercially available, all the other ligands were synthesized by condensation reactions. Some of them (compounds **2** and **3**) have been previously used for the functionalization of phosphorhydrazone dendrimers in a biological context as anti-cancer agents [15], but their synthesis and characterization were not described. Figure 2 displays all the potential ligands that have been synthesized (**2**–**6**) for this new study about catalysis.

**Scheme 1:**
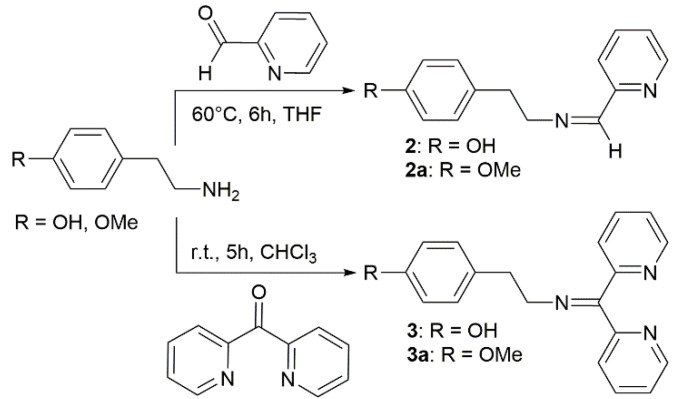
Synthesis of compounds **2**, **2a**, **3** and **3a**.

Compounds **2** and **3** are synthesized by condensation reactions between tyramine and either 2-pyridinecarboxaldehyde (for **2**) or di(2-pyridyl)ketone (for **3**). Analogous derivatives with a methoxy group (compounds **2a** and **3a**) instead of the hydroxyl group have been also synthesized, starting from 4-methoxyphenethylamine (family **a**) (Scheme 1).

**Scheme 2:**
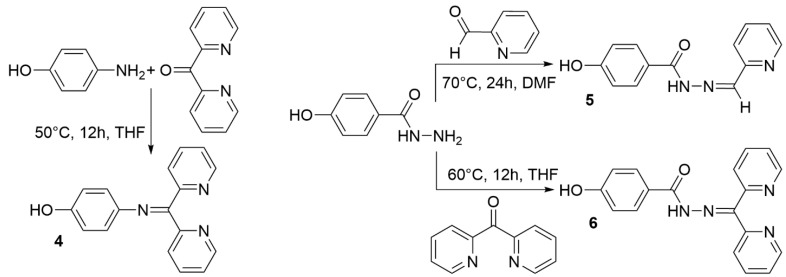
Synthesis of phenols **4**, **5**, and **6**.

2-pyridinecarboxaldehyde and di(2-pyridyl)ketone have been also condensed with other phenol-amine derivatives, such as 4-aminophenol to afford compound **4**, and 4-hydroxybenzhydrazide to afford compounds **5** and **6**. These reactions are shown in Scheme 2.

All these compounds have been characterized by ^1^H NMR, including COSY (correlation spectroscopy) and NOESY (nuclear Overhauser effect spectroscopy) experiments when necessary, and ^13^C NMR, including JMOD (J-modulated spin-echo) and HMQC (heteronuclear multiple-quantum correlation) experiments when necessary, to ascertain in particular the disappearance of the HCO or CO group. For compounds having two pyridine groups in their structure, such as compounds **3**, **4**, and **6**, each pyridine group is different, as illustrated by two different sets of signals both in ^1^H and ^13^C NMR spectra.

**Figure 3:**
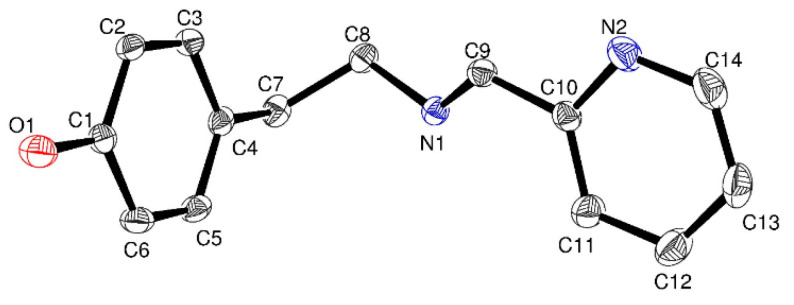
Crystal structure of compound **2**.

Several of these ligands have been characterized by X-ray diffraction studies. In all cases, the ellipsoids are represented with 50% probability. Figure 3 displays the crystal structure of the phenol derivative **2**. The formation of the C=N bond is demonstrated by the short N1-C9 distance (1.2671(17) Å). The dihedral angle N1-C9-C10-N2 (163.16(13)°), as well as the N1…N2 distance (3.534(2) Å) indicate that, in the crystal, the nitrogen atoms are in the *anti*-conformation.

**Figure 4:**
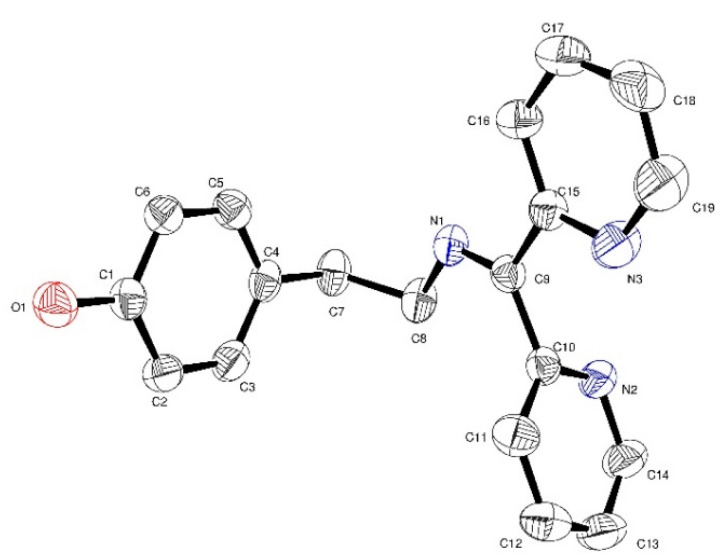
Crystal structure of compound **3**.

Figure 4 displays the crystal structure of compound **3**. The N1-C9 bond length (1.271(2) Å) corresponds well to a double bond. The two dihedral angles N-C-C-N are very different. The N1-C9-C10-N2 angle is 75.3(2)°, with an N1…N2 distance of 3.100(3) Å, indicating a tendency to a *syn*-conformation for this part of the molecule, which should be favorable for the complexation. The dihedral angle N1-C9-C15-N3 is −178.23(19)°, with N1…N3 of 3.539(3) Å, indicating a tendency to an *anti*-conformation of this part of the molecule. The N2…N3 distance is 3.503(3) Å. These data corroborate the presence of two very different sets of pyridine signals observed in the ^1^H and ^13^C NMR spectra of **3**.

**Figure 5:**
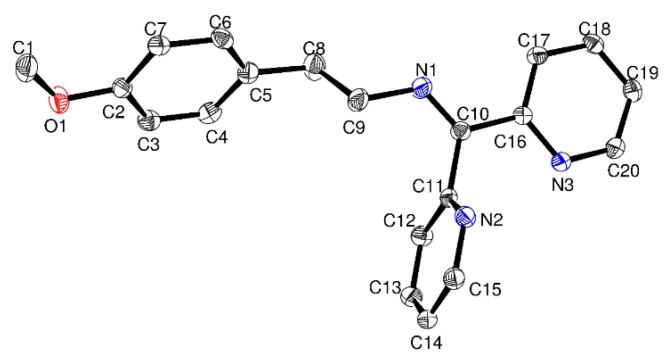
Crystal structure of compound **3a**.

The crystal structure of compound **3a** is shown in Figure 5. The only difference with compound **3** is the replacement of the HO group by an MeO group, thus only small changes are expected in the structure. The length of the imine bond N1-C10 (1.2714(17) Å) is very similar, and there are also two different pyridine groups. The dihedral angle N1-C10-C11-N2 is −94.06(16)°, and the N1…N2 distance is 3.261(2) Å; these values indicate a tendency to a *syn*-conformation. The dihedral angle N1-C10-C16-N3 is −175.73(12)°, and the N1…N3 distance is 3.550(2) Å, corresponding to an *anti*-conformation. The N2…N3 distance in compound **3a** (3.323(2) Å) is shorter than for **3** (3.503(3) Å).

**Figure 6:**
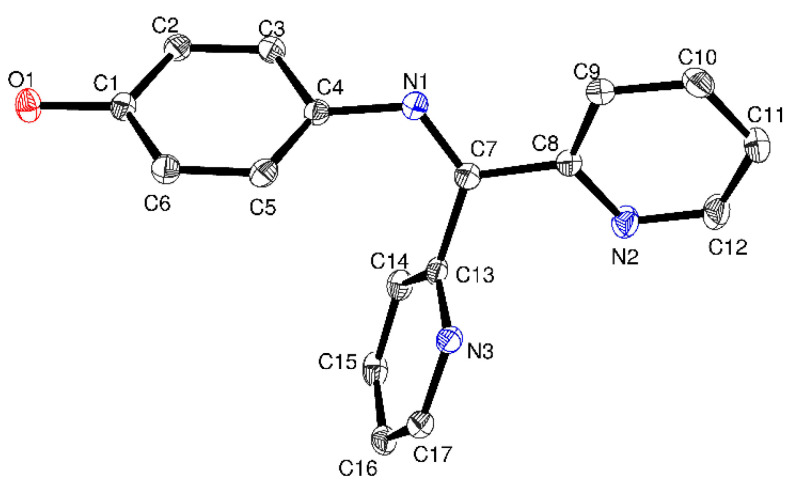
Crystal structure of compound **4**.

Figure 6 displays the crystal structure of compound **4**. The difference with compound **3** is that the imine bond is directly attached to the phenol group, instead to an ethyl chain used as linker. However, this difference has only a marginal influence on the imine bond length (N1-C7 = 1.2796(16) Å). The pyridine groups are different, but not as different as for the other compounds. The dihedral angle N1-C7-C8-N2 is −155.47(12)°, with an N1…N2 distance of 3.522(2) Å; the dihedral angle N1-C7-C13-N3 is −116.67(13)°, with an N1…N3 distance of 3.419(2) Å. Both pyridine groups are in *anti*-positions relative to the imine bond. The consequence is that the N2…N3 distance (between the nitrogen atoms of the pyridine groups) is the shortest of the series (3.180(2) Å).

**Figure 7:**
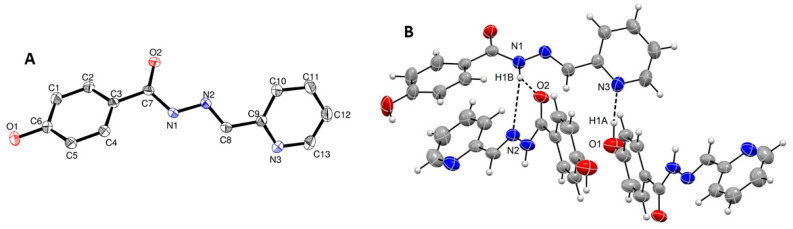
(**A**) Crystal structure of compound **5**. (**B**) Packing in the crystal showing the arrangement of three molecules of compound **5**, and the hydrogen bonds.

The crystal structure of compound **5** is shown in Figure 7. The difference with compound **2** is that the CH_2_-CH_2_ linkage is replaced by a CO-NH linkage. Despite these changes, the imine bond length is very similar (N2-C8 = 1.2763(16) Å), as well as the dihedral angle N2-C8-C9-N3 (−165.54(13)°) and the N2…N3 distance (3.530(2) Å). As expected when considering the packing of compound **5** in the crystal, the OH group is involved in H bonding with the nitrogen atom of another molecule (*d* H1A…N3 = 1.73(3) Å). In addition, the CO-NH linkage is also involved in hydrogen bonding. The hydrogen atom linked to N1 (H1B) interacts both with N2 and O2 of another molecule (d H1B…O2 = 2.146(18) Å, H1B…N2 = 2.535(18) Å). Both molecules are oriented head to tail (Figure 7B).

### 2.2. Grafting of The Functionalized Ligands to The Surface of Dendrimers

**Scheme 3:**
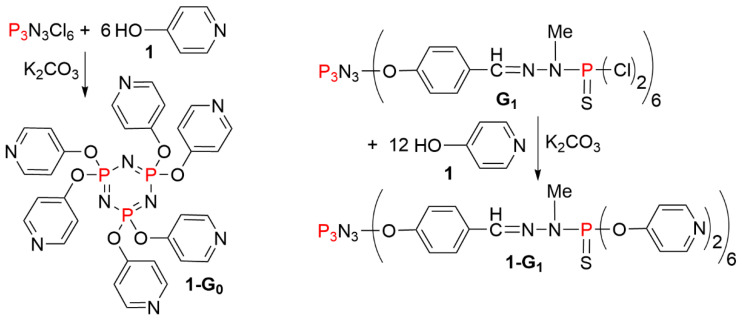
Synthesis of phosphorus dendrimers **1-G_0_** and **1-G_1_**, functionalized by the N-ligand **1**.

**Figure 8:**
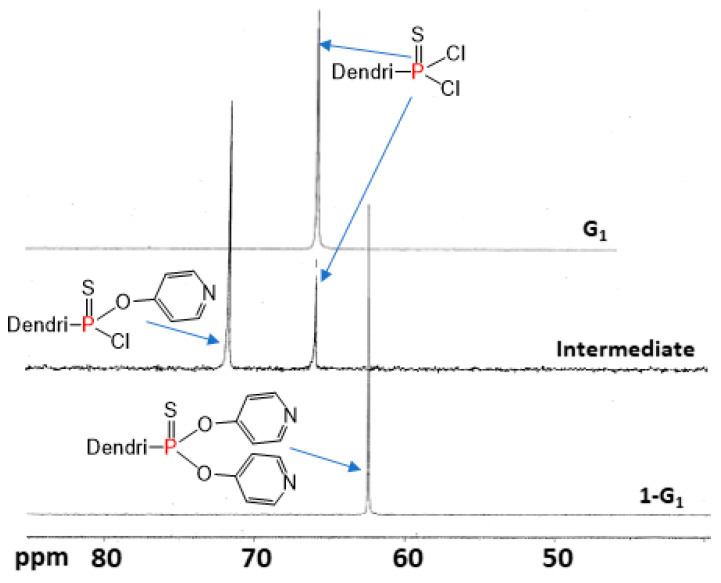
Changes in the ^31^P {^1^H} NMR spectra during the synthesis of dendrimer **1-G_1_** from **G_1_**. Only the part of the spectra corresponding to the terminal groups is shown.

The grafting of the ligands as terminal groups of the phosphorhydrazone dendrimers is carried out in all cases in the presence of a base, either potassium carbonate or cesium carbonate. Six equivalents of 4-hydroxypyridine **1** have been grafted to generation 0 of the dendrimers (N_3_P_3_Cl_6_). The reaction is monitored by ^31^P NMR, which displays the replacement of the singlet at δ = 20.8 ppm (N_3_P_3_Cl_6_) by a complex series of signals during the course of the reaction, followed by the appearance of a single singlet at δ = 10.2 ppm, corresponding to the full substitution, that is, to compound **1-G_0_**. The same reaction was carried out using 12 equivalents of compound **1** to react with the first generation of the dendrimer **G_1_**, which structure is shown in Figure 1 to afford dendrimer **1-G_1_** (Scheme 3). The reaction is also monitored by ^31^P NMR, which first displays the decrease of the signal corresponding to the P(S)Cl_2_ terminal functions (δ = 65.8 ppm) on behalf of the appearance of another singlet at δ = 71.7 ppm, corresponding to the monosubstitution on each terminal phosphorus atom (P(S)Cl(OC_5_H_4_N)). The full substitution affords another singlet at δ = 62.4 ppm. Figure 8 displays the ^31^P NMR spectra of the terminal groups of compounds for the reaction from **G_1_** to **1-G_1_**. Of course, in all cases the signal corresponding to the N_3_P_3_ core is also detected, at δ = 11.2 ppm in the case of dendrimer **1-G_1_**.

**Figure 9:**
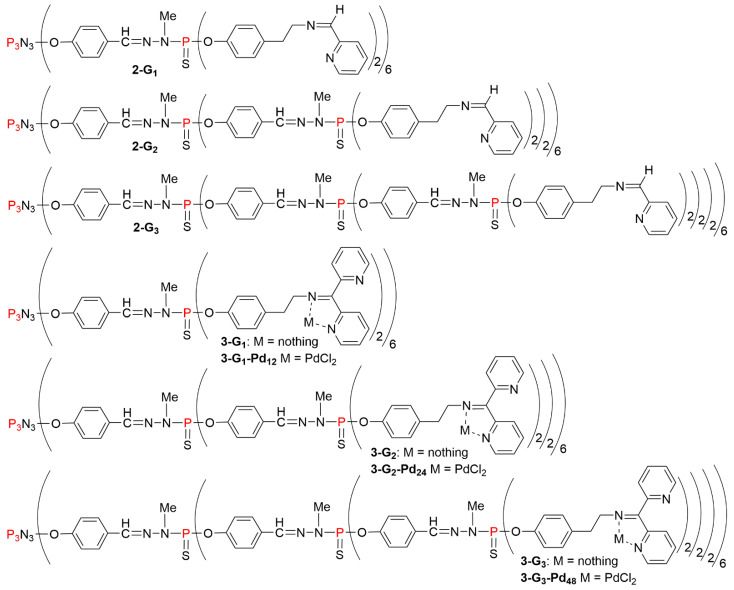
Phosphorhydrazone dendrimers from generations 1 to 3, functionalized with ligands **2** and **3**, affording dendrimers **2-Gn** and **3-Gn** respectively. Structure of the palladium complexes the **3-Gn** family, namely **3-Gn-Pdx** (*n* = 1–3, x = 12, 24, 48).

The reaction of ligand **2** has been carried out with phosphorhydrazone dendrimers of generations 1 to 3, that is, dendrimers **G_1_**, **G_2_**, and **G_3_**, in the presence of cesium carbonate as a base, using 12, 24, and 48 equivalents of ligand **2**, respectively. The synthesis of the resulting dendrimers **2-G_1_**, **2-G_2_**, and **2-G_3_** was carried out as previously reported [15], thus only their linear representation is shown in Figure 9. The same experiments, in the same conditions, were carried out with ligand **3** and generations 1, 2, and 3 of the phosphorhydrazone dendrimers, to afford dendrimers **3-G_1_**, **3-G_2_**, and **3-G_3_**, possessing 12, 24, and 48 ligands as terminal functions, respectively (Figure 9).

Ligand **4** is relatively similar to ligand **3**, but the nitrogen atom is directly linked to the aromatic in **4**, instead of an ethyl linker in the case of **3**. Despite an increased temperature and a longer reaction time, no reaction was observed when attempting to react ligand **4** with dendrimer **G_1_**, as shown by ^31^P NMR. Other attempts were made with compounds **5** and **6**, to be grafted to dendrimer **G_1_**. In these cases, the reaction begun easily, but the dendrimer precipitated before the completion of the reaction. Changing the solvent to DMF improved the advancement of the reaction. However, no fully substituted dendrimer could be isolated in these improved conditions.

### 2.3. Complexation of Palladium by Monomers and Dendrimers

**Figure 10:**
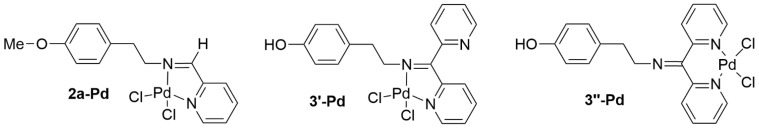
Complexation of compound **2a**, and the two possible forms of the Pd complex of **3**.

Having in hand several dendrimers functionalized with nitrogen ligands, their ability to complex palladium derivatives has been assayed. Compound **2a** was reacted with PdCl_2_COD (COD = cyclooctadiene) to afford compound **2a-Pd**, in which the complexation occurs between the nitrogen atoms of the imine function and of the pyridine group (Figure 10). In the case of the family of compounds **3**, there is an ambiguity concerning which nitrogen atoms are implied in the complexation. Indeed, the complexation may occur either between the two pyridines, to form a 6-membered ring, or between the imine and one pyridine, to form a 5-membered ring. The complexation was first carried out with ligand **3** and PdCl_2_COD to afford **3-Pd**. Characterization of **3-Pd** by ^1^H NMR could not ascertain the localization of palladium in this compound, either the form **3′-Pd** or **3′′-Pd** (Figure 10). Indeed, the chemical shifts of the hydrogen atoms on the carbon adjacent to the nitrogen atom of both pyridine rings are shifted upon complexation, from 8.45 and 8.65 ppm for **3** to 8.88 and 9.18 ppm, respectively, for **3-Pd**. This may correspond to form **3”-Pd**, as both signals are modified (Δδ = +0.43 and +0.53 ppm, respectively). ^13^C NMR affords additional information, when considering the carbon atom of the imine function, and both carbon atoms attached to it. Indeed, the imine carbon atom gives a signal at 166.6 ppm for **3**, and 176.3 ppm for **3-Pd** (Δδ = +9.7 ppm). Carbon atoms attached to the imine carbon give signals at 155.3 and 156.8 ppm for **3**, and 148.7 and 157.3 ppm for **3-Pd** (Δδ = −6.6 ppm, and +0.5 ppm, respectively). These data suggest that palladium in **3-Pd** should be complexed as shown in structure **3′-Pd**. In view of the contradictory results afforded by ^1^H and ^13^C NMR, it appears necessary to use another method for finding the real structure of compound **3-Pd**.

**Figure 11:**
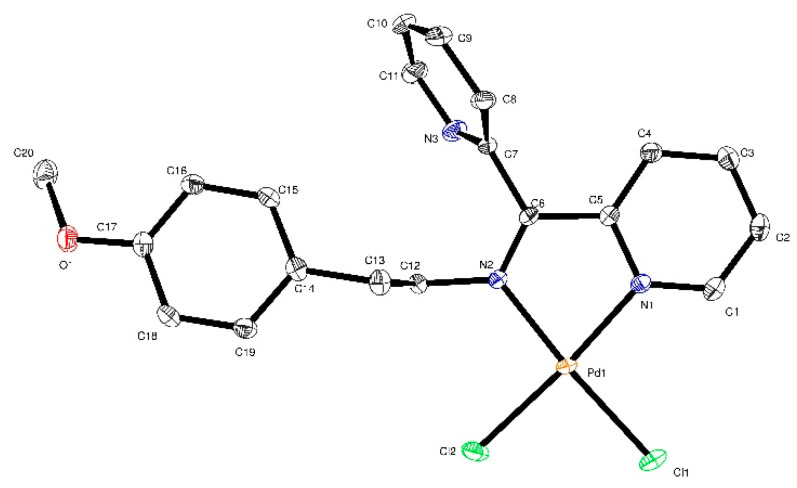
Crystal structure of the palladium complex **3a-Pd**.

In order to ascertain the location of the complexation with this type of multifunctional ligands, attempts were made to crystallize a palladium complex. Attempts were successful with **3a-Pd**, and the structure of this complex could be obtained by X-ray diffraction (Figure 11). This structure shows unambiguously that the complexation of palladium occurs between the imine and one pyridine group, thus corresponding to the form **3′-Pd** in Figure 10. A few other examples of bipyridine imine palladium complexes are known. The complexation between both pyridine groups was proposed in several cases [16,17], but each time a crystal structure was obtained, the complexation was observed between the imine and one pyridine [18,19,20,21]. In the case of compound **3a-Pd**, the imine bond (N2-C6 = 1.290(2) Å) is slightly longer than for the same (free) ligand **3a** (1.2796(16) Å). Obviously, the pyridine rings are very different. The dihedral angle N2-C6-C5-N1 is -2.9(2)° (almost flat), with the shortest N2…N1 distance of the series (2.613(2) Å). The dihedral angle N2-C6-C7-N3 of the non-complexed pyridine ring is −69.1(2)°, with a relatively short N2…N3 distance of 3.082(2) Å. On the contrary, the distance between the nitrogen atoms of both pyridine rings is the largest of the series, with N1 N3 = 4.623(2) Å. Of course, in solution, the free rotation around the C6-C7 bond can lead the N3 atom closer to palladium.

**Figure 12:**
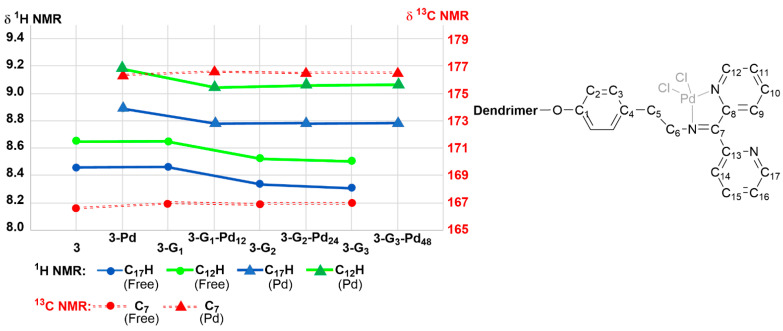
^1^H NMR data for the hydrogen atoms on the carbon adjacent to the nitrogen atom of both pyridine rings (C_12_-H and C_17_-H), and ^13^C NMR data of the carbon of the imine bond (C_7_), for monomer and dendrimers, free and complexed in both cases.

The same type of complexation was carried out with dendrimers **3-G_1_**, **3-G_2_**, and **3-G_3_**, using 12, 24, and 48 equivalents of PdCl_2_COD, respectively. The corresponding complexes **3-G_1_-Pd_12_**, **3-G_2_-Pd_24_** and **3-G_3_-Pd_48_** are less soluble than the non-complexed dendrimers. In consequence, the quality of the spectra is lower. However, ^31^P NMR data confirmed that there is no cleavage of the structure upon complexation. Furthermore, comparison of ^1^H NMR data between free and complexed dendrimers are coherent with the data obtained with the monomer **3** and **3-Pd**. Moreover, ^13^C NMR is very informative of the formation of the complexes, in particular with the presence of a signal at ca 176.4 ppm for the carbon of the imine bond, instead ca 167.0 for the non-complexed dendrimer (Figure 12). Thus, the complexation of the dendrimers **3-G_n_-Pd_12n_** should be analogous to that of the monomer **3a-Pd**, between the imine and one pyridine group, as shown in Figure 9.

### 2.4. Catalytic Attempts

Most of the nitrogen ligands shown in this paper contain Schiff bases in their structure. The importance of Schiff base complexes used as catalysts in a wide range of reactions such as polymerizations, oxidations, or additions, has been reviewed previously [22]. Being interested since a long time in catalyzed C–C cross-couplings [23], essentially with dendritic phosphine complexes [24], it appeared tempting to perform such types of reactions with nitrogen complexes. Schiff bases palladium complexes have been used as catalysts in Heck couplings, but in most cases, the phenol function pertaining to the ligand act also as ligand for the palladium [25,26]. The complexes can be either isolated or generated in situ. Thus, first attempts were carried out with the aim of determining if it was needed to isolate the complexes, or if mixing the dendrimers with palladium (II) was sufficient. The catalyzed coupling of iodobenzene with styrene was chosen as a test reaction. It was carried out essentially with the family of compounds **3**, monomers and dendrimers, either pre-complexed with PdCl_2_, or complexed in situ with Pd(OAc)_2_. The results are gathered in Table 1. In all cases 1 mol% of palladium was used. It means that the quantity of palladium is identical in all experiments. For instance, if one equivalent of generation 1 dendrimer is used, it will be compared with 12 equivalents of monomer, because the first generation dendrimer has 12 Pd complexes on its surface. At 40° C, only the in situ prepared complexes (entries 2 and 4) display a weak activity. At 70° C, all the complexes are efficient, but the preformed complexes seem slightly more efficient than the in situ prepared complexes (compare entry 5 to 6, or entry 7 to 8). Besides, the first generation of the dendrimer **3-G_1_-Pd_12_** (entry 7) is more efficient than the larger generations (entries 9 and 10). An attempt has been made with the N ligand **1-G_1_**, treated with 12 equivalents of Pd(OAc)_2_, but a poor efficiency was observed (entry 11 in Table 1). One may presume that a simple pyridine group is not sufficient to ensure a stable complexation of palladium.

Another series of experiments was carried out using again iodobenzene but with a more reactive alkene, namely butyl acrylate. In that case, all the experiments have been carried out at 70 °C with the in situ formed complexes (Table 2), as the in situ complexation is easier to carry out. Furthermore, the results obtained for the previous Heck couplings indicated that there is not a large difference in the catalytic efficiency between the pre-formed and in situ formed complexes. For the reaction of butyl acrylate, 1 mol% of palladium was used at the beginning (entries 1 to 4). In view of the excellent results obtained, the quantity of palladium was decreased to 0.1 mol%, remarkably with the same efficiency (entries 5 to 8 in Table 2).

In view of these interesting results with Heck couplings, other catalytic tests were carried out for another type of cross-coupling reactions, that is, Sonogashira couplings, using both the **2** and **3** families of ligands for the in situ complexation of Pd(OAc)_2_. The first example of Sonogashira coupling concerns 4-iodotoluene and phenylacetylene (Table 3). Experiments were carried out at 40 and 70 °C, with the monomer **2a** and the dendrimers **2-Gn** (generations 1, 2, and 3), in the presence of Pd(OAc)_2_ (one Pd per pyridine imine group in all cases). A positive dendritic effect [27] is observed at 40 °C, with an increase in the percentage of conversion, from the first generation (55%) to the second (69%) and the third (71%) generation. The second and the third generations perform better than the monomer. Of course, the quantity of pyridine imine groups and of palladium is kept constant in all experiments. Surprisingly, the results are worse when the temperature is increased to 70 °C.

A second example of Sonogashira couplings was attempted using iodo- or bromo-benzene and phenylacetylene, catalyzed by the in situ prepared Pd complexes of **1-G_1_** and of the family of compounds **3** (**3** and **3-Gn**, *n* = 1–3), in all cases at 40° C, in view of the results shown above. 100% conversion is observed with iodobenzene and 1 mol% of catalyst with all the compounds of the **3** family (monomer and dendrimers) (Table 4, entries 2 to 5). In contrast, the dendrimer **1-G_1_** affords only 66% conversion (entry 1), and thus it was not used in other experiments. In view of the good results obtained with the **3** family, experiments were carried out using a lower quantity of catalysts (0.1 instead of 1 mol%). In these conditions, the conversion was still good: 73% with compound **3** (entry 6) and 81% with dendrimer **3-G_1_** (entry 7). Experiments were also carried out with the more challenging bromobenzene, using 1 mol% of catalyst. Both **3** and **3-G_1_** afforded diphenylacetylene in 37% conversion (entries 8 and 9).

## 3. Discussion

Among the six phenol amino ligands shown in Figure 2, only compounds **1**-**3** were successfully grafted on the surface of the dendrimers. No reaction was observed with compound **4**. This absence of reactivity is possibly due to a large delocalization of the phenate form (impossible for instance in compound **3**), which should largely decrease its reactivity. The problem is different with compounds **5** and **6**, which reacted, but the dendrimer precipitated before completion of the reaction. The problem is presumably due to the hydrazide linkage which may induce multiple hydrogen bonding. We have already demonstrated that other types of hydrazide derivatives (namely P and T Girard’s reagents) linked to phosphorhydrazone dendrimers or dendrons induced the spontaneous formation of hydrogels in water [28,29,30,31], characteristic of a decreased solubility of the dendrimer. Furthermore, the packing in the crystal structure of compound **5** (Figure 7B) has pointed out the presence of such interactions, in a head to tail arrangement, which will favor the interactions between dendrimers, thus decreasing largely the solubility.

The first catalytic attempts were carried out with the aim of determining if it was needed to isolate the complexes, or if creating it in situ was suitable. As there was no large difference in the catalytic efficiency between both methods, in situ formation of the complex was chosen in most cases. On the contrary, there are large differences in the catalytic efficiency depending on the type of ligands. It can be shown from the comparison between Table 3 and Table 4 that the family of compounds **3**, which possesses one imine and two pyridine groups, affords more efficient catalysts than the family of compounds **2**, which also possesses one imine, but only one pyridine group. The difference might be related to the presence of a pyridine group in the second coordination sphere in the series **3** but which does not exist in the series **2**. Indeed, the second pyridine group possibly acts as a ligand of the second coordination sphere, which is well known to play a key role in catalysis [32], including for Heck couplings [33].

Our catalytic attempts compare well with recently published results, using nitrogen complexes of palladium. As an example, 2,2′-(bisdiamino)azobenzene ligands (H_2_L) complexing Pd ([(HL)Pd(PPh_3_)]ClO_4_) was used as a catalyst (1 mol%) in refluxing THF for Heck coupling of iodobenzene with styrene. *trans*-stilbene was isolated in 94% yield [34]. This result compares well with 90–98% conversion obtained in our case (Table 1). The coupling of iodobenzene and butyl acrylate is generally easier than the coupling with styrene. The monomer and the three generations of the **3** family are particularly efficient as the yield is 100% even when using only 0.1 mol% of catalyst. This result is better than a recent example using 0.8 mol% of dendrimers complexing palladium and linked to silica, used as catalysts in the same reaction [35].

In a third example, N,N-bidentate ligands bearing N=P^V^-C-C-NH backbones were ligands complexing PdCl_2_, which was used as catalyst (1 mol%) in the Sonogashira coupling of 4-iodotoluene with phenylacetylene in hard conditions (110° C in DMF). 1-methyl-4-(2-phenylethynyl)-benzene was obtained in 97% yield (measured by GC) [36]. Our experiments were carried out in really milder conditions (40° C instead of 110° C) and in the presence of water (CH_3_CN/water (6:1)) and afforded 1-methyl-4-(2-phenylethynyl)-benzene in reasonable yields (71% with the generation 3 dendrimer) as shown in Table 3. In a fourth example, glycine was the ligand of PdCl_2_, affording complex [PdCl_2_(NH_2_CH_2_COOH)_2_], which was used as catalyst (1 mol%) in the Sonogashira coupling of iodobenzene with phenylacetylene at 60° C. Diphenyl acetylene was isolate in 30% to 93% yield, depending on the solvent used, the best result being obtained in water/acetone (3/3mL) [37]. Our results in CH_3_CN/water (6:1) are carried out in milder conditions (40°C) (Table 4), and the conversion was quantitative with 1 mol% of dendritic catalysts. The experiments carried out with 0.1 mol% dendritic catalysts, which afforded 81% conversion, are better than those obtained previously.

## 4. Materials and Methods

The NMR data were obtained with Bruker AV 300, DPX 300 or AV 400. Chemical shifts are reported in ppm, relative to SiMe_4_ for ^1^H and ^13^C NMR, and to 85% H_3_PO_4_ for ^31^P NMR. Catalytic experiments have been carried out with a Radley system (Carousel12 Reaction Station).

Crystallography data were collected on a Bruker Kappa Apex II diffractometer equipped with a 30 W air-cooled microfocus source (**3a-Pd**), on a Rigaku Oxford Gemini EOS dual source diffractometer (**2**, **3a**, **4** and **5**), or on a STOE Imaging Plate diffractometer System (**3**), with MoK_α_ radiation (λ = 0.71073 Å) or CuK_α_ radiation (λ = 1.54184 Å). Cooler devices were used to collect the data at low temperature (180(2) K or 100(2) K for **3a-Pd**). Phi- and omega- scans were performed for data collection and an empirical absorption correction was applied. The structures were solved by intrinsic phasing method (SHELXT) [38] or by direct methods with SIR92 [39] and refined by means of least-squares procedures on F² with SHELXL [40] and on F with CRYSTALS [41]. All non-hydrogen atoms were refined anisotropically. Hydrogen atoms were located in a difference map but those attached to carbon atoms were repositioned geometrically and then refined using a riding model. For **5**, the solvent molecules were highly disordered and difficult to model correctly. Therefore, the SQUEEZE function of PLATON [42] was used to eliminate the contribution of the electron density in the solvent region from the intensity data, and the solvent-free model was employed for the final refinement. CCDC 1991349, 1991350, 1991351, 1991352, 1991353 and 1991354 contain the supplementary crystallographic data for compounds **2**, **3**, **3a**, **3a-Pd**, **4** and **5**, respectively. These data can be obtained free of charge via http://www.ccdc.cam.ac.uk/conts/retrieving.html (accessed on 1 April 2021), or from the Cambridge Crystallographic Data Centre, 12 Union Road, Cambridge CB2 1EZ, UK; fax: (+44) 1223-336-033; or email: deposit@ccdc.cam.ac.uk.

**Figure 13:**
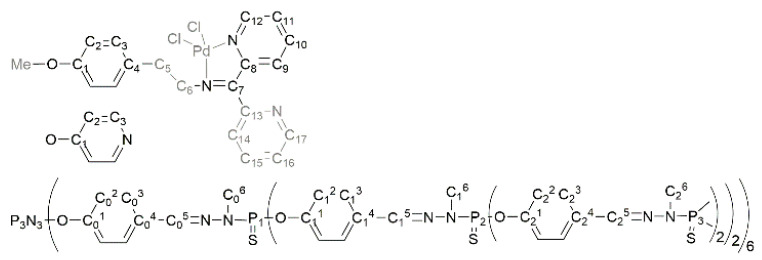
Numbering used for NMR assignment. The atoms in grey color are not present in some compounds.

All reactions were carried out under argon, generally using Schlenk tubes. Solvents were dried before use. Dendrimers **2-Gn** (*n* = 1–3) and **3-Gn** (*n* = 1–3) have been synthesized according to published procedures [15], as well as compound **1-G_0_** [43]. The numbering used for NMR assignment of the ligands and dendrimers is shown in Figure 13. Dendrimers are able to entrap solvent molecules, which are very difficult to eliminate, thus perturbing elemental analyses. Mass analyses cannot be performed for dendrimers due to cleavages and rearrangement at the level of the hydrazone linkages [44].

**Compound 2**. In a Schlenk tube, a mixture of 1g (7.25 mmol) of tyramine, 1 equivalent of pyridine-2-carboxaldehyde (0.775 g), 20 mL of THF, and molecular sieves were heated at 60° C for 6h. The mixture was filtered, and the solution was centrifuged to eliminate traces of molecular sieves. The solution was evaporated to dryness, then compound **2** was recrystallized in absolute ethanol, to afford compound **2** as a white powder in 84% yield. Crystals of **2** suitable for X-ray diffraction were obtained by a further recrystallization in absolute ethanol. ^1^H NMR (300 MHz, CDCl_3_): *δ* = 3.00 (t, *^3^J_HH_* = 9Hz, 2H, C_5_H), 3.91 (t, *^3^J_HH_* = 9Hz, 2H, C_6_H), 5.15 (s, 1H, OH), 6.77 (d, *^3^J_HH_* = 8.4 Hz, 2H, C_2_H), 7.09 (d, *^3^J_HH_* = 8.4 Hz, 2H, C_3_H), 7.34 (dd, *^3^J_HH_* = 8 Hz, 1H, C_11_H), 7.9 (dd, *^3^J_HH_* = 8 Hz, 1H, C_10_H), 8.00 (d, *^3^J_HH_* = 8 Hz, 1H, C_9_H), 8.30 (s, 1H, C_7_H), 8.65 (d, *^3^J_HH_* = 4 Hz, 1H, C_12_H). ^13^C {^1^H} NMR (75 MHz, CDCl_3_): *δ* = 36.2 (C_5_), 62.9 (C_6_), 115.5 (C_2_), 121.5 (C_9_), 125.0 (C_11_), 129.9 (C_3_), 130.9 (C_4_), 137.1 (C_10_), 149.1 (C_12_), 154.1 (C_8_), 154.8 (C_1_), 162.2 (C_7_).

**Compound 2a.** In a Schlenk tube, a mixture of 1g (6.61 mmol) of 4-methoxyphenethylamine, 1.1 equivalent of pyridine-2-carboxaldehyde (0.779 g), 15 mL of THF, and molecular sieves were heated at 60° C for 6h. The mixture was filtered, then evaporated to dryness, to afford an oil, which was evaporated to dryness overnight. Compound **2a** was obtained as an orange oil in 51% yield. ^1^H NMR (400 MHz, DMSO-d_6_): *δ* = 2.89 (t, *^3^J_HH_* = 8 Hz, 2H, C_5_H), 3.69 (s, 3H, CH_3_), 3.84 (t, *^3^J_HH_* = 8 Hz, 2H, C_6_H), 6.83 (d, *^3^J_HH_* = 8 Hz, 2H, C_2_H), 7.16 (d, *^3^J_HH_* = 8 Hz, 2H, C_3_H), 7.38 (dd, *^3^J_HH_* = 8 Hz, 1H, C_11_H), 7.81 (dd, *^3^J_HH_* = 8 Hz, 1H, C_10_H), 7.98 (d, *^3^J_HH_* = 8 Hz, 1H, C_9_H), 8.29 (s, 1H, C_7_H), 8.62 (d, *^3^J_HH_* = 4 Hz, 1H, C_12_H). ^13^C {^1^H} NMR (100 MHz, DMSO-d_6_): *δ* = 36.28 (C_5_), 55.30 (CH_3_), 62.57 (C_6_), 114.07 (C_2_), 120.80 (C_9_), 125.36 (C_11_), 130.21 (C_3_), 131.99 (C_4_), 137.09 (C_10_), 149.71 (C_12_), 154.71 (C_8_), 158.08 (C_1_), 162.43 (C_7_).

**Compound 3**. Into a small round bottom flask were added 1.3 g (7.1 mmol) of di(2-pyridyl)ketone, 0.97 g (7.1 mmol) of tyramine and three small spoons of MgSO_4_. To the flask was added 7.5 mL of chloroform giving a slurry. After five hours of reaction (supervised by TLC THF/Hexane (1:1)) a filtration and evaporation were made. The product **3** was a white powder, obtained in 79% yield. Recrystallization in DCM gave colorless crystals of **3**, suitable for analysis by X-ray diffraction. ^1^H NMR (300 MHz, CDCl_3_/THF-d_8_): *δ* = 2.92 (t, *^3^J_HH_* = 7.5 Hz, 2H, C_5_H), 3.61 (t, *^3^J_HH_* = 7.5 Hz, 2H, C_6_H), 6.61 (d, *^3^J_HH_* = 8.4 Hz, 2H, C_2_H), 6.91 (d, *^3^J_HH_* = 8.4 Hz, 2H, C_3_H), 6.95 (d, ^3^J_HH_ = 7.8 Hz, 1H, C_9_H), 7.23 (m, 2H, C_11_H, C_16_H), 7.69 (m, 2H, C_10_H, C_15_H), 8.10 (d, *^3^J_HH_* = 8.1 Hz, 1H, C_14_H), 8.45 (d, *^3^J_HH_* = 4.2 Hz, 1H, C_17_H), 8.65 (d, ^3^J_HH_ = 4.8 Hz, 1H, C_12_H). ^13^C {^1^H} NMR (75 MHz, CDCl_3_/THF-d_8_): *δ* = 36.45 (C_5_), 55.85 (C_6_), 115.07 (C_2_), 122.19 (C_14_), 122.91 (C_11_), 123.56 (C_9_), 123.96 (C_16_), 129.71 (C_3_), 130.83 (C_4_), 135.84 (C_10_), 136.21 (C_15_), 148.64 (C_17_), 149.40 (C_12_), 155.33 (C_8_), 155.42 (C_1_), 156.85 (C_13_), 166.63 (C_7_).

**Compound 3a**. Into a small round bottom flask were added 1.0 g (5.5 mmol) of di(2-pyridyl) ketone, 0.83 g (5.5 mmol) of methoxyphenethylamine and three small spoons of MgSO_4_ in 20mL of THF. After five hours of reaction (supervised by TLC THF/Hexane (1:1)) a filtration and evaporation were made. The resulting powder was washed with dichloromethane/pentane. The product **3a** was a white powder, obtained in 80% yield. Recrystallized in DCM gave colorless crystals of **3a**, suitable for analysis by X-ray diffraction. ^1^H NMR (400 MHz, CDCl_3_): *δ* = 3.02 (t, *^3^J_HH_* = 8 Hz, 2H, C_5_H), 3.68 (t, *^3^J_HH_* = 6 Hz, 2H, C_6_H), 3.79 (s, 3H, CH_3_), 6.82 (d, *^3^J_HH_* = 8.4 Hz, 2H, C_2_H), 7.03 (d, *^3^J_HH_* = 7.8 Hz, 1H, C_9_H), 7.07 (d, *^3^J_HH_* = 8.4 Hz, 2H, C_3_H), 7.30 (m, 2H, C_11_H, C_16_H), 7.75 (m, 2H, C_10_H, C_15_H), 8.14 (d, *^3^J_HH_* = 8 Hz, 1H, C_14_H), 8.53 (br d, *^3^J_HH_* = 4.2 Hz, 1H, C_17_H), 8.73 (br d, ^3^J_HH_ = 4.8 Hz, 1H, C_12_H). ^13^C {^1^H} NMR (100 MHz, CDCl_3_): *δ* = 36.49 (C_5_), 55.24 (CH_3_), 55.78 (C_6_), 113.71 (C_2_), 122.23 (C_14_), 123.04 (C_11_), 123.51 (C_9_), 124.10 (C_16_), 129.83 (C_3_), 132.26 (C_4_), 135.96 (C_10_), 136.37 (C_15_), 148.88 (C_17_), 149.66 (C_12_), 155.34 (C_8_), 156.88 (C_1_), 157.96 (C_13_), 166.85 (C_7_).

**Compound 4**. In a Schlenk tube were mixed 4-amino phenol (296 mg, 2.71 mmol), di(2-pyridyl) ketone (500 mg, 2.71 mmol) and THF (10 mL) in the presence of activated molecular sieves. The mixture was stirred at 50° C for 12 h. Progress of the reaction was monitored by thin layer chromatography (TLC) with THF/pentane as eluent (v:v 1/1). The mixture was filtered, evaporated to dryness, and then recrystallized in dichloromethane. Yellow crystals of **4** were obtained in 58% yield. One of them was chosen for X-ray diffraction studies. ^1^H NMR (400 MHz, DMSO-d_6_): *δ* = 6.56 (s, 4H, C_2_, C_3_), 7.15 (d, *^3^J_HH_* = 8 Hz, 1H, C_9_), 7.30 (dd, *^3^J_HH_* = 4 Hz, 1H, C_11_), 7.46 (dd, *^3^J_HH_* = 4 Hz, 1H, C_16_), 7.71 (dd, *^3^J_HH_* = 8 Hz, 1H, C_10_), 7.95 (dd, *^3^J_HH_* = 8 Hz, 1H, C_15_), 8.23 (d, *^3^J_HH_* = 8 Hz, 1H, C_14_), 8.49 (d, *^3^J_HH_* = 4 Hz, 1H, C_17_), 8.54 (d, *^3^J_HH_* = 4 Hz, 1H, C_12_), 9.26 (s, 1H, OH). ^13^C {^1^H} NMR (100 MHz, DMSO-d_6_): *δ* = 115.5 (C_3_), 122.7 (C_14_), 123.1 (C_2_), 123.5 (C_11_), 124.8 (C_9_), 125.2 (C_16_), 136.3 (C_10_), 137.3 (C_15_), 141.7 (C_4_), 148.9 (C_17_), 149.4 (C_12_), 154.8 (C_1_), 156.0 (C_8_), 157.2 (C_13_), 165.5 (C_7_).

**Compound 5**. In a Schlenk tube were mixed 4-hydroxybenzhydrazide (500 mg, 3.28 mmol), pyridine-2-carboxaldehyde (352 mg, 3.28 mmol) and DMF (30 mL) in the presence of activated molecular sieves. The mixture was stirred at 70° C for 24 h, then 10 mL of DMF were added. The mixture was filtered, evaporated to dryness, by co-evaporation with toluene. The compound was then recrystallized in absolute ethanol. Compound **5** was obtained as a white powder in 45% yield. A further recrystallization in absolute ethanol afforded crystals of **5** suitable for X-ray diffraction studies. ^1^H NMR (400 MHz, DMSO-d_6_): *δ* = 6.88 (d, *^3^J_HH_* = 8 Hz, 2H, C_2_), 7.40 (dd, *^3^J_HH_* = 4 Hz, 1H, C_11_), 7.81 (d, *^3^J_HH_* = 8 Hz, 2H, C_3_), 7.87 (d, *^3^J_HH_* = 8 Hz, 1H, C_9_), 7.95 (m, 1H, C_10_), 8.45 (s, 1H, C_7_), 8.61 (d, *^3^J_HH_* = 4 Hz, 1H, C_12_), 10.11 (s, 1H, OH), 11.83 (s, 1H, NH). ^13^C {^1^H} NMR (75 MHz, DMSO-d_6_): *δ* = 115.5 (C_2_), 120.2 (C_10_), 124.1 (C_11_), 124.7 (C_4_), 130.3 (C_3_), 137.3 (C_9_), 147.5 (C_7_), 149.9 (C_12_), 153.9 (C_8_), 161.3 (C_1_), 163.5 (C_5_).

**Compound 6**. In a Schlenk tube were mixed 4-hydroxybenzhydrazide (410 mg, 2.7 mmol), di(2-pyridyl) ketone (500 mg, 2.7 mmol) and THF (15 mL). The mixture was stirred at 60° C for 15 h. Progress of the reaction was monitored by TLC. The mixture was evaporated to dryness and was washed 2 times with THF/pentane (1:2), to afford compound **6** as a yellow powder in 92% yield. ^1^H NMR (300 MHz, DMSO-d_6_): *δ* = 6.95 (d, *^3^J_HH_* = 8.4 Hz, 2H, C_2_H), 7.49 (m, 2H, C_9_H and C_16_H), 7.61 (m, 1H, C_11_H), 7.80 (d, *^3^J_HH_* = 8.4 Hz, 2H, C_3_H), 7.92 (d, *^3^J_HH_* = 8.1 Hz, 1H, C_14_H), 8.02 (m, 2H, C_10_H, and C_15_H), 8.62 (d, *^3^J_HH_* = 4.5 Hz, 1H, C_17_H), 8.98 (d, *^3^J_HH_* = 4.5 Hz, 1H, C_12_H). ^13^C {^1^H} NMR (75 MHz, DMSO-d_6_): *δ* = 116.1 (C_2_), 123.8 (C_14_), 124.3 (C_11_), 125.2 (C_9_), 127.5 (C_16_), 130.0 (br s, C_3_ and C_4_), 137.7 (C_10_), 138.3 (C_15_), 145.6 (C_7_), 148.6 (C_17_), 148.9 (C_12_), 152.0 (C_8_), 156.2 (C_1_), 161.7 (C_13_), 163.1 (br s, C_5_).

**Dendrimer 1-G_1_**. In a round bottom flask, we added 0.512 g (0.28 mmol) of first generation dendrimer **G_1_**, 0.351 g (3.7 mmol) of p-hydroxypyridine **1** and 1.02 g (7.4 mmol) of K_2_CO_3_. To these solids were added 50 mL of acetone and the reaction was left stirring overnight. The solvent was evaporated, and the resulting powder was solubilized with CHCl_3_, then filtrated over Celite, washed with CHCl_3_ and evaporated to dryness. This process was repeated one time to afford dendrimer **1-G_1_** as a pale yellow powder in 57% yield. ^31^P {^1^H} NMR (81 MHz, CDCl_3_): *δ* = 11.20 (N_3_P_3_), 62.39 (P_1_). ^1^H NMR (200 MHz, CDCl_3_), 3.34 (d, *^3^J_HP_* = 10.7 Hz, 18H, C_0_^6^H), 7.02 (d, *^3^J_HH_* = 8.5 Hz, 12H, C_0_^2^H), 7.13 (d, *^3^J_HH_* = 4.8 Hz, 24H, C_2_H), 7.51 (d, *^3^J_HH_* = 8.5 Hz, 12H, C_0_^3^H), 7.58 (s, 6H, C_0_^5^H), 8.51 (d, ^3^*J_HH_* = 4.8 Hz, 24H, C_3_H). ^13^C {^1^H} NMR (50 MHz, CDCl_3_): *δ* = 32.68 (d, *^2^J_CP_* = 13.0 Hz, C_0_^6^), 116.28 (d, *^3^J_CP_* = 4.5 Hz, C_2_), 121.27 (C_0_^2^), 128.19 (C_0_^3^), 131.57 (C_0_^4^), 139.58 (d, *^3^J_CP_* = 14.5 Hz, C_0_^5^), 150.79 (br s, C_0_^1^), 151.60 (C_3_), 157.15 (d, *^2^J_CP_* = 7.2 Hz, C_1_).

**Compound 2a-Pd.** In a Schlenk tube 0.18 g (0.631 mmoles) of PdCl_2_COD and 0.154 g (0.631 mmoles) of ligand **2a** were mixed in 10 mL of THF. The mixture was stirred at room temperature for 22h. After filtration, the product was washed with THF/pentane (1/10), then with THF/ether (1:10). Complex **2a-Pd** was obtained as a yellow-orange powder in 75% yield. ^1^H NMR (400 MHz, DMSO-d_6_): *δ* = 3.04 (t, *^3^J_HH_* = 8 Hz, 2H, C_5_H), 3.71 (s, 3H, CH_3_), 3.89 (t, *^3^J_HH_* = 8 Hz, 2H, C_6_H), 6.88 (d, *^3^J_HH_* = 8 Hz, 2H, C_2_H), 7.21 (d, *^3^J_HH_* = 8 Hz, 2H, C_3_H), 7.88 (dd, *^3^J_HH_* = 8 Hz, 1H, C_11_H), 8.04 (d, *^3^J_HH_* = 8 Hz, 1H, C_9_H), 8.33 (dd, *^3^J_HH_* = 8 Hz, 1H, C_10_H), 8.41 (s, 1H, C_7_H), 8.99 (d, *^3^J_HH_* = 4 Hz, 1H, C_12_H). ^13^C {^1^H} NMR (100 MHz, DMSO-d_6_): *δ* = 35.77 (C_5_), 55.48 (Me), 61.11 (C_6_), 114.44 (C_2_), 128.57 (C_11_), 129.11 (C_9_), 130.04 (C_3_), 130.46 (C_4_), 141.87 (C_10_), 150.69 (C_12_), 156.15 (C_8_), 158.45 (C_1_), 171.82 (C_7_).

**Compound 3-Pd**. In a Schlenk tube were mixed 0.19 g (0.66 mmoles) of PdCl_2_COD and 0.2 g (0.66 mmoles) of ligand **3** in 10 mL of THF. The mixture was stirred at room temperature for 15h. After filtration, the product was washed two times with THF/pentane (1/10), and one time with THF/ether (1:10). Complex **3-Pd** was obtained as a yellow powder in 81% yield. ^1^H NMR (300 MHz, DMSO-d_6_): *δ* = 3.00 (m, 2H, C_5_H), 3.65 (m, 2H, C_6_H), 6.60 (d, *^3^J_HH_* = 8.4 Hz, 2H, C_2_H), 6.78 (d, *^3^J_HH_* = 8.4 Hz, 2H, C_3_H), 7.21 (d, *^3^J_HH_* = 7.8 Hz, 1H, C_9_H), 7.73 (m, 2H, C_11_H, C_14_H), 7.93 (m, 1H, C_16_H), 8.11 (m, 1H, C_10_H), 8.21 (m, 1H, C_15_H), 8.88 (d, *^3^J_HH_* = 4.2 Hz, 1H, C_17_H), 9.18 (d, *^3^J_HH_* = 4.8 Hz, 1H, C_12_H), 9.22 (s, 1H, HO). ^13^C {^1^H} NMR (75 MHz, THF-d_8_): *δ* = 35.85 (C_5_), 57.2 (C_6_), 115.8 (C_2_), 124.9 (C_14_), 126.5 (C_11_), 129.5 (C_9_), 129.7 (C_16_), 130.0 (C_3_), 128.1 (C_4_), 138.4 (C_10_), 141.8 (C_15_), 148.7 (C_8_), 150.9 (C_17_), 151.05 (C_12_), 156.5 (C_1_), 157.3 (C_13_), 176.3 (C_7_).

**Compound 3a-Pd**. In a Schlenk tube were mixed 0.20 g (0.631 mmol) of ligand **3a** and 0.18 g (0.631 mmol) of PdCl_2_COD, in 10 mL of THF. The mixture was stirred at room temperature for 20h, affording a yellow-orange mixture. After filtration, the product was washed with THF/pentane (1/10), then with THF/ether. Single crystals were obtained by slow evaporation at room temperature of solutions of **3a-Pd** in dichloromethane. ^1^H NMR (400 MHz, DMF-d_6_): *δ* = 3.31 (br s, 2H, C_5_H), 3.94 (s, 3H, Me), 4.05 (br s, 2H, C_6_H), 7.00 (d, *^3^J_HH_* = 8 Hz, 2H, C_2_H), 7.21 (d, *^3^J_HH_* = 8 Hz, 2H, C_3_H), 7.58 (d, *^3^J_HH_* = 7.8 Hz, 1H, C_9_H), 7.99 (m, H, C_11_H), 8.11 (d, *^3^J_HH_* = 8 Hz, 1H, C_14_H), 8.21 (m, 1H, C_16_H), 8.40 (t, *^3^J_HH_* = 8 Hz, 1H, C_15_H), 8.52 (t, *^3^J_HH_* = 8 Hz, 1H, C_10_H), 9.14 (d, *^3^J_HH_* = 4 Hz, 1H, C_17_H), 9.52 (d, *^3^J_HH_* = 4 Hz, 1H, C_12_H). ^13^C {^1^H} NMR (100 MHz, DMF-d_6_): *δ* = 35.95 (C_5_), 55.02 (Me), 57.22 (C_6_), 114.19 (C_2_), 124.98 (C_14_), 126.33 (C_11_), 129.25 (C_9_), 129.60 (C_16_), 129.85 (C_3_), 130.14 (C_4_), 138.20 (C_10_), 141.54 (C_15_), 149.04 (C_8_), 150.90 (C_17_), 151.01 (C_12_), 157.53 (C_1_), 158.79 (C_13_), 176.41 (C_7_).

**Dendrimer 3-G_1_-Pd**. Into a Schlenk tube were added 400 mg (0.08 mmol) of dendrimer **3-G_1_** and 273 mg (0.95 mmol) of PdCl_2_(COD) to10 mL of THF. The reaction was left stirring at room temperature for 15 h. A solid had started to precipitate in the Schlenk. The solvent was evaporated and the product was washed twice with THF/pentane (1:10), and then with THF/ether. Dendrimer **3-G_1_-Pd** was obtained as a pale yellow powder in 86% yield. ^31^P {^1^H} NMR (121 MHz, DMSO-d_6_): *δ* = 8.5 (N_3_P_3_), 62.6 (P_1_). ^1^H NMR (300 MHz, DMSO-d_6_): *δ* = 2.97 (m, 24H, C_5_H), 3.17 (d, *^3^J_CP_* = 10.2 Hz, 18H, C_0_^6^H), 3.60 (m, 24H, C_6_H), 6.93 (br m, 60H, C_0_^2^H, C_2_H, C_3_H), 7.16 (d, *^3^J_HH_* = 7.8 Hz, 12H, C_9_H), 7.54 (m, 12H, C_11_H), 7.57 (d+s, *^3^J_HH_* = 8.4 Hz, 12H, C_0_^3^H and 6H, C_0_^5^H), 7.73 (d, *^3^J_HH_* = 8.1 Hz, 1H, C_14_H), 7.85 (m, 12H, C_16_H), 8.02 (m, 12H, C_10_H), 8.18 (m, 12H, C_15_H), 8.78 (d, *^3^J_HH_* = 4.5 Hz, 12H, C_17_H), 9.04 (d, *^3^J_HH_* = 4.5 Hz, 12H, C_12_H). ^13^C {^1^H} NMR (75 MHz, DMSO-d_6_): *δ* = 33.36 (d, *^2^J_CP_* = 12 Hz, C_0_^6^), 35.85 (C_5_), 56.91 (C_6_), 121.48 (C_0_^2^, C_2_), 125.01 (C_14_), 126. 44 (C_11_), 128.66 (C_0_^3^), 129.55 (C_9_), 129.82 (C_16_), 130.27 (C_3_), 132.47 (C_0_^4^), 135.37 (C_4_), 138.24 (C_10_), 140.20 (br, C_0_^5^), 141.77 (C_15_), 148.48 (C_8_), 149.11 (d, *^2^J_CP_* = 7 Hz, C_1_), 150.39 (C_17_), 150.85 (C_0_^1^), 150.99 (C_12_), 157.25 (C_13_), 176.46 (C_7_).

**Dendrimer 3-G_2_-Pd**. Same experimental procedure as for **1-G_1_-Pd**, but with 200 mg (0.018 mmol) of dendrimer **3-G_2_** and 122 mg (0.43 mmol) of PdCl_2_(COD) in10 mL of THF. The dendrimer **3-G_2_-Pd** was obtained as a yellow powder in 76% yield. ^31^P {^1^H} NMR (121 MHz, DMSO-d_6_): *δ* = 8.3 (N_3_P_3_), 62.5 (br s, P_2_, P_1_). ^1^H NMR (300 MHz, DMSO-d_6_): *δ* = 3.01 (m, 48H, C_5_H), 3.20 (m, 54H, C_0_^6^H, C_1_^6^H), 3.60 (m, 48H, C_6_H), 6.93 (br m, 108H, C_0_^2^H, C_2_H, C_3_H), 7.13 (br m, 72H, C_1_^2^H, C_9_H, C_11_H), 7.62 (br m, 102H, C_0_^3^H, C_0_^6^H, C_1_^3^H, C_1_^6^H, C_14_H, C_16_H), 8.00 (br m, 24H, C_10_H), 8.13 (br m, 24H, C_15_H), 8.77 (br m, 24H, C_17_H), 9.07 (br m, 24H, C_12_H). ^13^C {^1^H} NMR (75 MHz, DMSO-d_6_): *δ* = 33.42 (m, C_0_^6^, C_1_^6^), 35.90 (C_5_), 56.78 (C_6_), 121.48 (C_0_^2^, C_1_^2^, C_2_), 124.94 (C_14_), 126. 48 (C_11_), 128.87 (C_0_^3^, C_1_^3^, C_9_), 129.72 (C_16_), 130.28 (C_3_), 132.50 (C_0_^4^, C_1_^4^), 135.27 (C_4_), 138.24 (C_10_), 140.20 (br, C_0_^5^, C_1_^5^), 141.72 (C_15_), 148.42 (C_8_), 149.12 (C_1_), 150.80 (br, C_0_^1^, C_1_^1^, C_12_, C_17_), 157.15 (C_13_), 176.39 (C_7_).

**Dendrimer 3-G_3_-Pd**. Same experimental procedure as for **1-G_1_-Pd**, but with 200 mg (0. 0085 mmol) of dendrimer **3-G_3_** and 117 mg (0.41 mmol) of PdCl_2_(COD) in10 mL of THF. The dendrimer **3-G_3_-Pd** was obtained as a yellow powder in 74% yield. ^31^P {^1^H} NMR (121 MHz, DMSO-d_6_): *δ* = 8.1 (br s, N_3_P_3_), 62.5 (br s, P_3,_ P _2,_ P _1_). ^1^H NMR (300 MHz, DMSO-d_6_): *δ* = 3.00 (m C_5_H, 96H), 3.21 (m, 126H, C_0_^6^H, C_1_^6^H, C_2_^6^H), 3.60 (m, 96H, C_6_H), 6.93 (br m, 204H, C_0_^2^H, C_2_H, C_3_H), 7.10 (br m, 168H, C_1_^2^H, C_2_^2^H, C_9_H, C_11_H), 7.62 (br m, 222H, C_0_^3^H, C_0_^6^H, C_1_^3^H, C_1_^6^H, C_2_^3^H, C_2_^6^H, C_14_H, C_16_H), 7.99 (br m, 48H, C_10_H), 8.11 (br m, 48H, C_15_H), 8.75 (m, 48H, C_17_H), 9.03 (m, 48H, C_12_H). ^13^C {^1^H} NMR (75 MHz, DMSO-d_6_): *δ* = 33.48 (m, C_0_^6^, C_1_^6^, C_2_^6^), 35.90 (C_5_), 56.73 (C_6_), 121.49 (C_0_^2^, C_1_^2^, C_2_^2^, C_2_), 124.78 (C_14_), 126. 50 (C_11_), 128.87 (C_0_^3^, C_1_^3^, C_2_^3^, C_9_), 129.56 (C_16_), 130.30 (C_3_), 132.50 (C_0_^4^, C_1_^4^, C_2_^4^), 135.26 (C_4_), 138.22 (C_10_), 140.20 (br, C_0_^5^, C_1_^5^, C_2_^5^), 141.69 (C_15_), 148.40 (C_8_), 149.30 (C_1_), 151.09 (br, C_0_^1^, C_1_^1^, C_2_^1^, C_12_, C_17_), 157.11 (C_13_), 176.37 (C_7_).

## 5. Conclusions

A series of N-, N,N- and N,N,N-ligands based on pyridine and pyridine-imine functions, and further functionalized with a phenol (eventually protected by a methyl) has been synthesized. Characterization of most of these compounds by X-ray diffraction studies confirms their structure. The phenols have been tentatively grafted to phosphorhydrazone dendrimers, generally up to the third generation (48 ligands on the surface). Three families of dendrimers have been synthesized, possessing either one pyridine group on each terminal function (**1-Gn**, *n* = 0, 1), or one pyridine imine (**2**, **2a**, **2-Gn**, *n* = 1–3), or two pyridines and one imine (**3**, **3a**, **3-Gn**, *n* = 1–3). The PdCl_2_ complexes of the family of compounds **3** have been synthesized and isolated. The crystal structure of compound **3a-Pd** was recorded, to ascertain the location of Pd. The complexation was not between the two pyridine groups (as often proposed in the literature), but between the imine and one pyridine.

The isolated Pd complexes of the family **3**, and also the complexes prepared in situ by the complexation of Pd(OAc)_2_ with the **1**, **2**, and **3** families of compounds have been tested in several catalytic C-C cross-coupling reactions, of type Heck and Sonogashira. The family **1** (only pyridine terminal functions) was found poorly efficient in both cases. Family **2** was found relatively efficient for Sonogashira couplings, and displayed a positive dendritic effect, the best results being obtained with the third generation **2-G_3_**. However, this family of compounds is less efficient than the family of compounds **3**, which was thus applied in more catalytic tests. With this family **3**, 0.1 mol% of Pd was sufficient for catalyzing Heck couplings between iodobenzene and butyl acrylate, and also for Sonogashira couplings between iodobenzene and phenylacetylene. These results compare very well in terms of quantity of metal and of temperature with the best results in the literature concerning catalysis with Schiff bases palladium complexes, as emphasized in a recent review [27].

## Figures and Tables

**Table 1:**
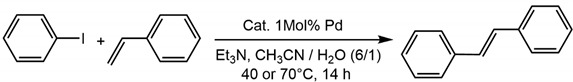
Catalysis of Heck couplings between iodobenzene and styrene.

Entry	T °C	Catalyst Pd	Conversion %
1	40	**3-Pd**	0
2	40	**3** + Pd(OAc)_2_	12
3	40	**3-G_1_-Pd_12_**	0
4	40	**3-G_1_** + 12 Pd(OAc)_2_	5
5	70	**3-Pd**	98
6	70	**3** + Pd(OAc)_2_	91
7	70	**3-G_1_-Pd_12_**	90
8	70	**3-G_1_** + 12 Pd(OAc)_2_	80
9	70	**3-G_2_-Pd_24_**	60
10	70	**3-G_3_-Pd_48_**	66
11	70	**1-G_1_** + 12 Pd(OAc)_2_	16

**Table 2:**
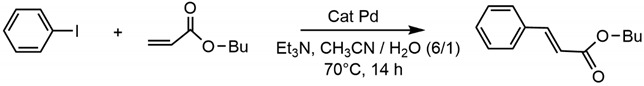
Catalysis of Heck couplings between iodobenzene and butyl acrylate.

Entry	Mol % Pd	Catalyst Pd	Conversion %
1	1	**3** + Pd(OAc)_2_	100
2	1	**3-G_1_** + 12 Pd(OAc)_2_	100
3	1	**3-G_2_** + 24 Pd(OAc)_2_	100
4	1	**3-G_3_** + 48 Pd(OAc)_2_	100
5	0.1	**3** + Pd(OAc)_2_	100
6	0.1	**3-G_1_** + 12 Pd(OAc)_2_	100
7	0.1	**3-G_2_** + 24 Pd(OAc)_2_	100
8	0.1	**3-G_3_** + 48 Pd(OAc)_2_	100

**Table 3:**
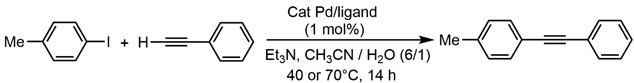
Catalysis of Sonogashira couplings between iodotoluene and phenylacetylene.

Entry	T °C	Catalyst Pd	Conversion %
1	40	**2a** + Pd(OAc)_2_	64
2	40	**2-G_1_** + 12 Pd(OAc)_2_	55
3	40	**2-G_2_** + 24 Pd(OAc)_2_	69
4	40	**2-G_3_** + 48 Pd(OAc)_2_	71
5	70	**2a** + Pd(OAc)_2_	68
6	70	**2-G_1_** + 12 Pd(OAc)_2_	53
7	70	**2-G_2_** + 24 Pd(OAc)_2_	61
8	70	**2-G_3_** + 48 Pd(OAc)_2_	59

**Table 4:**
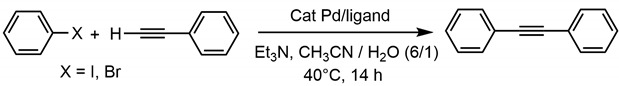
Catalysis of Sonogashira couplings between iodo- or bromo-benzene and phenylacetylene, at 40° C.

Entry	X	Mol % Pd	Catalyst Pd	Conversion %
1	I	1	**1-G_1_** + 12 Pd(OAc)_2_	66
2	I	1	**3** + Pd(OAc)_2_	100
3	I	1	**3-G_1_** + 12 Pd(OAc)_2_	100
4	I	1	**3-G_2_** + 24 Pd(OAc)_2_	100
5	I	1	**3-G_3_** + 48 Pd(OAc)_2_	100
6	I	0.1	**3** + Pd(OAc)_2_	73
7	I	0.1	**3-G_1_** + 12 Pd(OAc)_2_	81
8	Br	1	**3** + Pd(OAc)_2_	37.5
9	Br	1	**3-G_1_** + 12 Pd(OAc)_2_	37

## Data Availability

Data is contained within the article or supplementary material.

## Supplementary Materials

The following are available online, NMR spectra (^1^H, ^13^C and ^31^P NMR) of all new compounds (**2**, **2a**, **2a-Pd**, **3**, **3a**, **3-Pd**, **3a-Pd**, **4**, **5**, **6**, **1-G_0_**, **1-G_1_**, **3-G_1_-Pd_12_**, **3-G_2_-Pd_24_**, **3-G_3_-Pd_48_**). CIF files for compounds **2**, **3**, **3a**, **3a-Pd**, **4** and **5**.

## Sample Availability

No samples of compounds are available from the authors.
