# Supplementary material for: Dendrimers Functionalized with Palladium Complexes of N-, N,N-, and N,N,N-Ligands
Source: Molecules. 2021 Apr 17;26(8):2333. doi: 10.3390/molecules26082333 (PMC8073930; doi:10.3390/molecules26082333)
Supplement: Supplementary file 1 [file molecules-26-02333-s001.zip › molecules-1179491-supplementary.pdf]

Supporting information belonging to the paper

Dendrimers Functionalized with Palladium Complexes of N-, N,N-, and N,N,N-Ligands

Quentin Vanbellingen, Paul Servin, Anaïs Coinaud, Sonia Mallet-Ladeira, Régis Laurent, Anne-Marie Caminade*

Table of contents

^{1}H NMR of Compound **2** **S3**
^{1}H COSY NMR of Compound **2** **S4**
^{1}H TOCSY NMR of Compound **2** **S5**
^{1}C {^{2}H} NMR of Compound **2** **S6**
^{33}C JMOD NMR of Compound **2** **S7**
^{4}C DEPT NMR of Compound **2** **S8**
^{5}C HMBC NMR of Compound **2** **S9**
^{63}C HMQC NMR of Compound **2** **S10**
^{7}H NMR of Compound **2a** **S11**
^{8}C {^{1}H} NMR of Compound **2a** **S12**
^{9}C JMOD NMR of Compound **2a** **S13**
^{10}C HMQC NMR of Compound **2a** **S14**
^{11}H NMR of Compound **2a-Pd**
S15
^{12}C {^{1}H} NMR of Compound **2a-Pd** **S16**
^{13}C HMQC NMR of Compound **2a-Pd**
S17
^{1}H NMR of Compound **3** **S18**

---

[1] C{^{1}H} NMR of Compound **3a-Pd** **S29**
[2] H NMR of Compound **4** **S30**
[3] H COSY NMR of Compound **4** **S31**
[4] C{^{1}H} NMR of Compound **4** **S32**
[5] C HMQC NMR of Compound **4** **S33** ^{13}C HMQC long range NMR of Compound **4** **S34**
[6] H NMR of Compound **5** **S35**
[7] H COSY NMR of Compound **5** **S36**
[8] C{^{1}H} NMR of Compound **5** **S37**
[9] C HMBC NMR of Compound **5** **S38**
[10] C HMQC NMR of Compound **5** **S39**
[11] H NMR of Compound **6** **S40**
[12] C{^{1}H} NMR of Compound **6** **S41**

^{1}H COSY NMR of Compound **3** **S19**
^{1}H NOESY NMR of Compound **3** **S20**
^{13}C {^{1}H} NMR of Compound **3** **S21**
^{13}C JMOD NMR of Compound **3** **S22**
^{13}C HMQC NMR of Compound **3** **S23**
^{1}H NMR of Compound **3a** **S24**
^{13}C{^{1}H} NMR of Compound **3a** **S25**
^{1}H NMR of Compound **3-Pd** **S26**
^{13}C{^{1}H} NMR of Compound **3-Pd** **S27**
^{1}H NMR of Compound **3a-Pd**
S28 ^{13}C HMQC of Compound **6**
S42
^{31}P {^{1}H} NMR during the course of the synthesis of **1-G$_0$** **S43**
^{31}P {^{1}H} NMR of **1-G$_0$** **S44**
^{31}P {^{1}H} NMR of dendrimer **1-G$_1$** **S45**
^{1}H NMR of dendrimer **1-G$_1$** **S46**
^{13}C {^{1}H} NMR of dendrimer **1-G$_1$** **S47**
^{31}P {^{1}H} NMR of dendrimer **3-G$_1$-Pd$_{12}$**
S48
^{1}H NMR of dendrimer **3-G$_1$-Pd$_{12}$** **S49**
^{13}C {^{1}H} NMR of dendrimer **3-G$_1$-Pd$_{12}$**
S50
^{13}C JMOD NMR of dendrimer **3-G$_1$-Pd$_{12}$** **S51**
^{13}C HMQC NMR of dendrimer **3-G$_1$-Pd$_{12}$** **S52**
^{31}P {^{1}H} NMR of dendrimer **3-G$_2$-Pd$_{24}$**
S53
^{1}H NMR of dendrimer **3-G$_2$-Pd$_{24}$** **S54**
^{13}C {^{1}H} NMR of dendrimer **3-G$_2$-Pd$_{24}$**
S55
^{13}C JMOD NMR of dendrimer **3-G$_2$-Pd$_{24}$** **S56**
^{31}P {^{1}H} NMR of dendrimer **3-G$_3$-Pd$_{48}$**
S57
^{1}H NMR of dendrimer **3-G$_3$-Pd$_{48}$** **S58**
^{13}C {^{1}H} NMR of dendrimer **3-G$_3$-Pd$_{48}$**
S59

^{1}H NMR of Compound 2

1

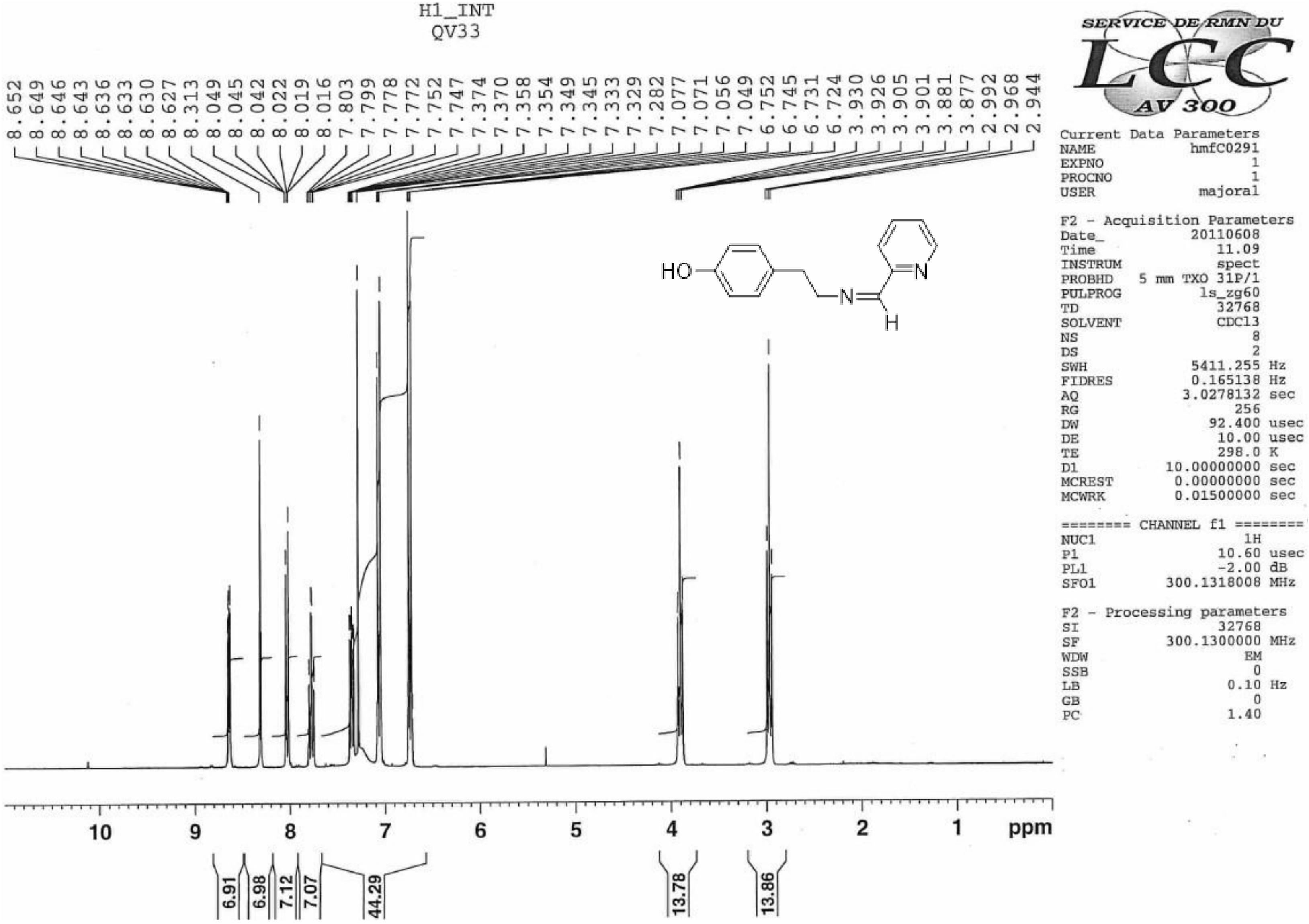
H1_INT
QV33
8.652
8.649
8.646
8.643
8.636
8.633
8.630
8.627
8.313
8.049
8.045
8.042
8.022
8.019
8.016
7.803
7.799
7.778
7.772
7.752
7.747
7.374
7.370
7.358
7.354
7.349
7.345
7.333
7.329
7.282
7.077
7.071
7.056
7.049
6.752
6.745
6.731
6.724
3.930
3.926
3.905
3.901
3.881
3.877
2.992
2.968
2.944
HO
N
N
H
10
9
8
7
6
5
4
3
2
1
ppm
6.91
6.98
7.12
7.07
44.29
13.78
13.86
SERVICE DE RMN DU
LCC
AV 300
Current Data Parameters
NAME hmfC0291
EXPNO 1
PROCNO 1
USER majoral
F2 - Acquisition Parameters
Date_ 20110608
Time 11.09
INSTRUM spect
PROBHD 5 mm TXO 31P/1
PULPROG ls_zg60
TD 32768
SOLVENT CDCl3
NS 8
DS 2
SWH 5411.255 Hz
FIDRES 0.165138 Hz
AQ 3.0278132 sec
RG 256
DW 92.400 usec
DE 10.00 usec
TE 298.0 K
D1 10.00000000 sec
MCREST 0.00000000 sec
MCWRK 0.01500000 sec
======== CHANNEL f1 ========
NUC1 1H
P1 10.60 usec
PL1 -2.00 dB
SFO1 300.1318008 MHz
F2 - Processing parameters
SI 32768
SF 300.1300000 MHz
WDW EM
SSB 0
LB 0.10 Hz
GB 0
PC 1.40

^{1}H COSY NMR of Compound 2

1

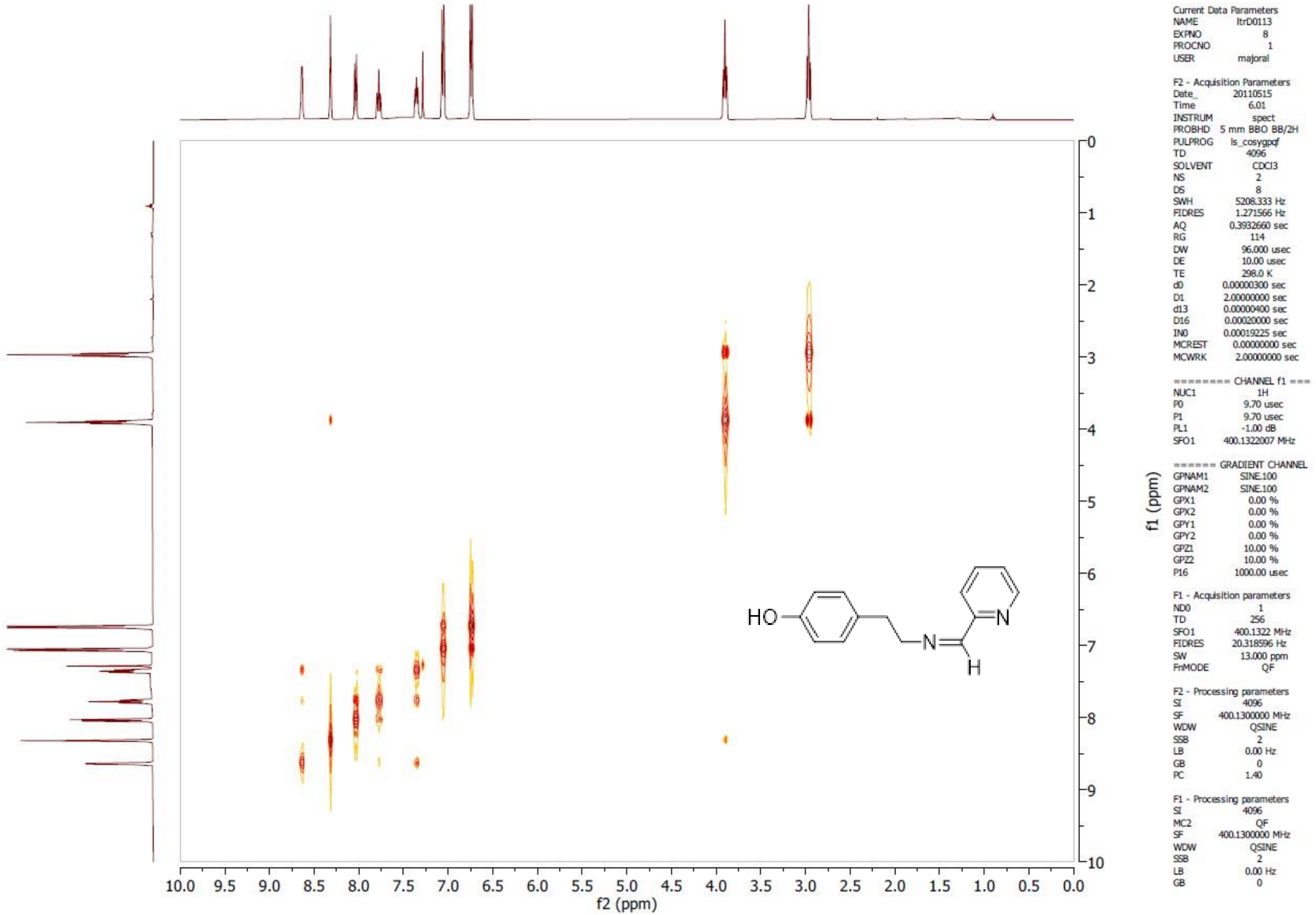
HO
N
N
H
f2 (ppm)
f1 (ppm)
10.0 9.5 9.0 8.5 8.0 7.5 7.0 6.5 6.0 5.5 5.0 4.5 4.0 3.5 3.0 2.5 2.0 1.5 1.0 0.5 0.0
0 1 2 3 4 5 6 7 8 9 10
Current Data Parameters
NAME ltrD0113
EXPNO 8
PROCNO 1
USER majoral
F2 - Acquisition Parameters
Date_ 20110515
Time 6.01
INSTRUM spect
PROBHD 5 mm BBO BB/2H
PULPROG ls_cosygpqf
TD 4096
SOLVENT CDCl3
NS 2
DS 8
SWH 5208.333 Hz
FIDRES 1.271566 Hz
AQ 0.3932660 sec
RG 114
DW 96.000 usec
DE 10.00 usec
TE 298.0 K
d0 0.00000300 sec
D1 2.00000000 sec
d13 0.00000400 sec
D16 0.00020000 sec
IN0 0.00019225 sec
MCREST 0.00000000 sec
MCWRK 2.00000000 sec
======== CHANNEL f1 ===
NUC1 1H
P0 9.70 usec
P1 9.70 usec
PL1 -1.00 dB
SFO1 400.1322007 MHz
====== GRADIENT CHANNEL
GPNAM1 SINE.100
GPNAM2 SINE.100
GPX1 0.00 %
GPX2 0.00 %
GPY1 0.00 %
GPY2 0.00 %
GPZ1 10.00 %
GPZ2 10.00 %
P16 1000.00 usec
F1 - Acquisition parameters
ND0 1
TD 256
SFO1 400.1322 MHz
FIDRES 20.318596 Hz
SW 13.000 ppm
FnMODE QF
F2 - Processing parameters
SI 4096
SF 400.1300000 MHz
WDW QSINE
SSB 2
LB 0.00 Hz
GB 0
PC 1.40
F1 - Processing parameters
SI 4096
MC2 QF
SF 400.1300000 MHz
WDW QSINE
SSB 2
LB 0.00 Hz
GB 0

^{1}H TOCSY NMR of Compound 2

1

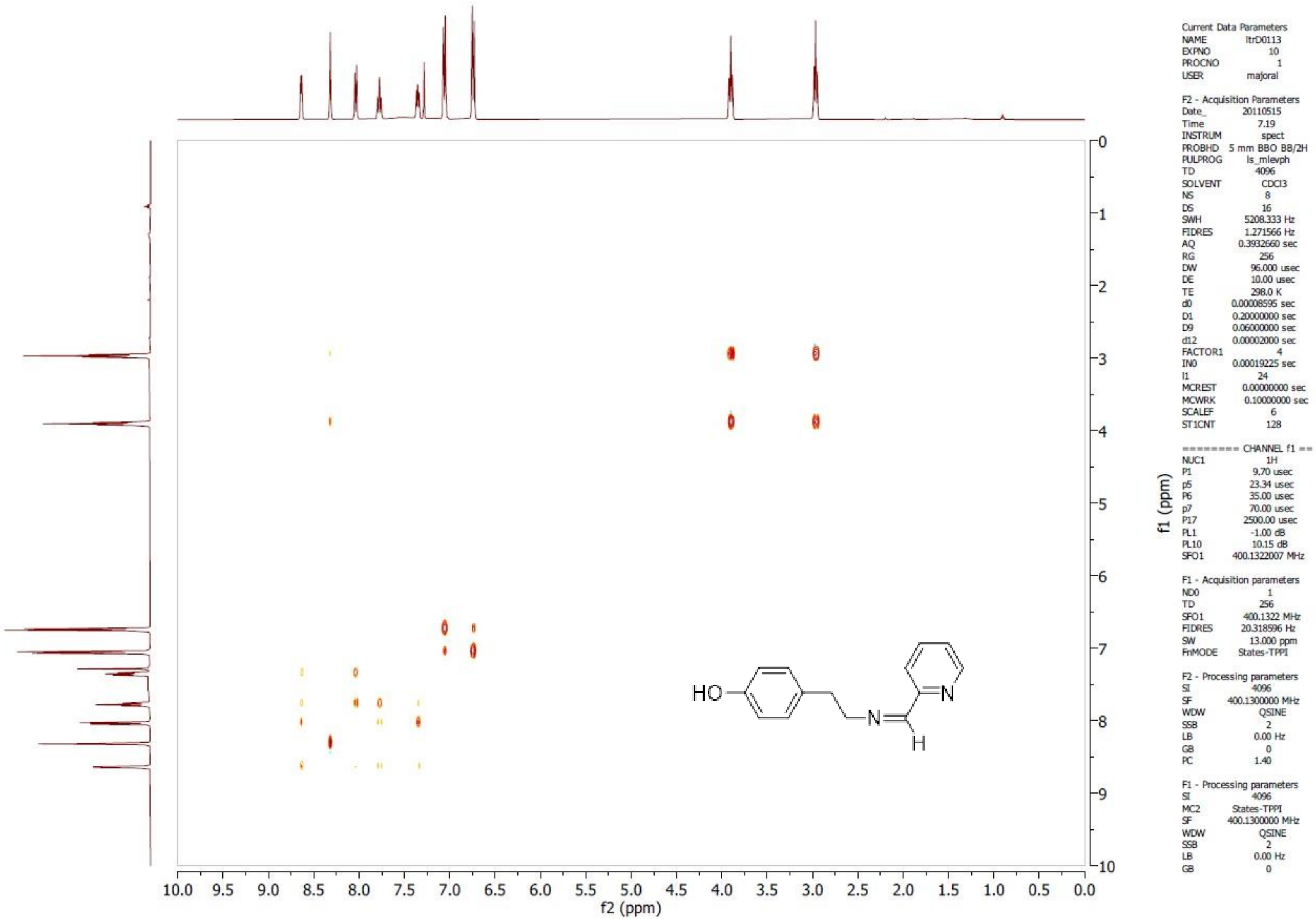
HO
N
N
H
f2 (ppm)
f1 (ppm)
10.0 9.5 9.0 8.5 8.0 7.5 7.0 6.5 6.0 5.5 5.0 4.5 4.0 3.5 3.0 2.5 2.0 1.5 1.0 0.5 0.0
0 1 2 3 4 5 6 7 8 9 10
Current Data Parameters
NAME ltrD0113
EXPNO 10
PROCNO 1
USER majoral
F2 - Acquisition Parameters
Date_ 20110515
Time 7.19
INSTRUM spect
PROBHD 5 mm BBO BB/2H
PULPROG ls_mlevph
TD 4096
SOLVENT CDCl3
NS 8
DS 16
SWH 5208.333 Hz
FIDRES 1.271566 Hz
AQ 0.3932660 sec
RG 256
DW 96.000 usec
DE 10.00 usec
TE 298.0 K
d0 0.00008595 sec
D1 0.20000000 sec
D9 0.06000000 sec
d12 0.00002000 sec
FACTOR1 4
IN0 0.00019225 sec
l1 24
MCREST 0.00000000 sec
MCWRK 0.10000000 sec
SCALEF 6
ST1CNT 128
======== CHANNEL f1 ==
NUC1 1H
P1 9.70 usec
p5 23.34 usec
P6 35.00 usec
p7 70.00 usec
P17 2500.00 usec
PL1 -1.00 dB
PL10 10.15 dB
SFO1 400.1322007 MHz
F1 - Acquisition parameters
ND0 1
TD 256
SFO1 400.1322 MHz
FIDRES 20.318596 Hz
SW 13.000 ppm
FnMODE States-TPPI
F2 - Processing parameters
SI 4096
SF 400.1300000 MHz
WDW QSINE
SSB 2
LB 0.00 Hz
GB 0
PC 1.40
F1 - Processing parameters
SI 4096
MC2 States-TPPI
SF 400.1300000 MHz
WDW QSINE
SSB 2
LB 0.00 Hz
GB 0

^{13}C {^{1}H} NMR of Compound 2

^{13}C

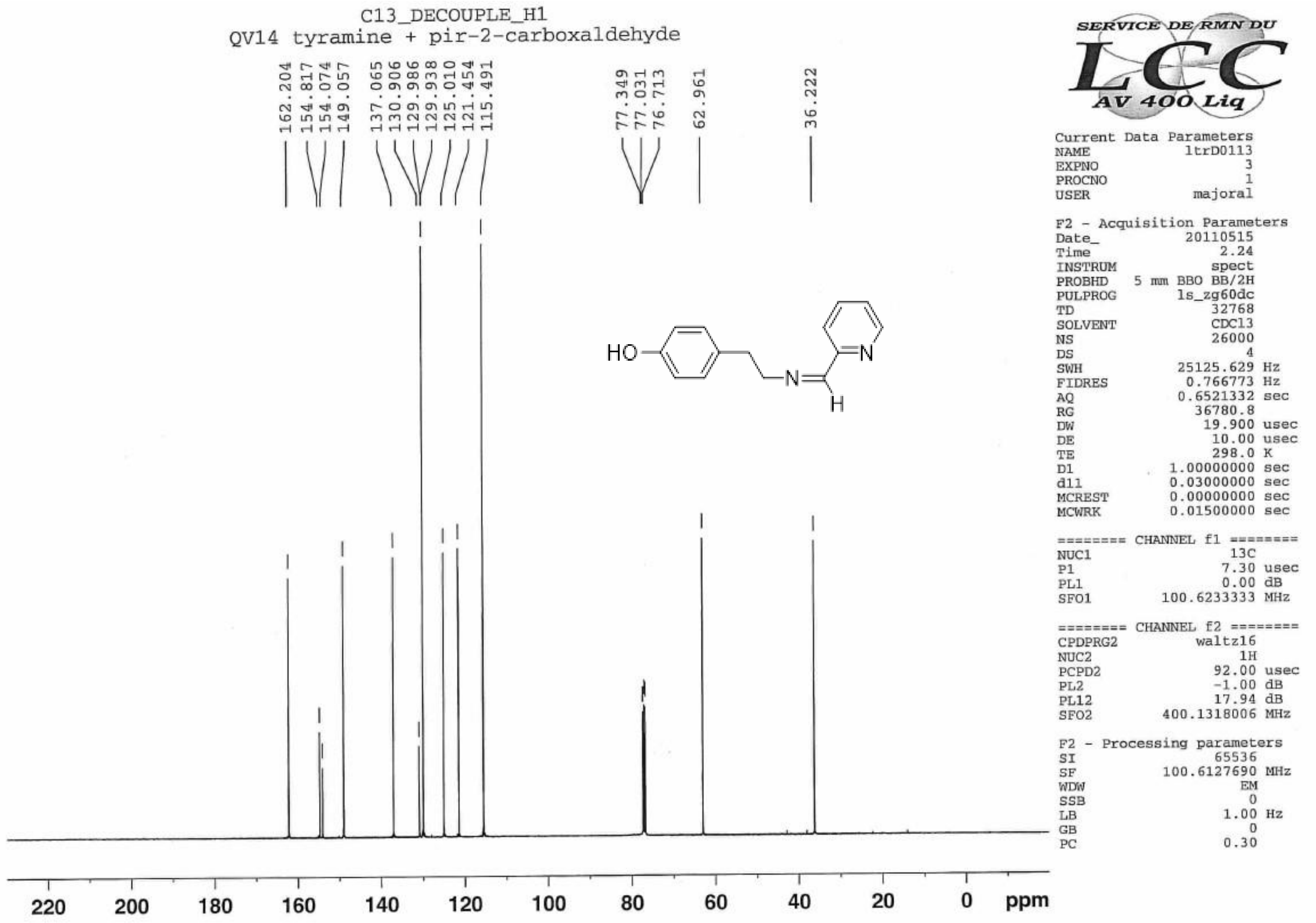
C13_DECOUPLE_H1
QV14 tyramine + pir-2-carboxaldehyde
162.204
154.817
154.074
149.057
137.065
130.906
129.986
129.938
125.010
121.454
115.491
77.349
77.031
76.713
62.961
36.222
HO
N
N
H
SERVICE DE RMN DU
LCC
AV 400 Liq
Current Data Parameters
NAME 1trD0113
EXPNO 3
PROCNO 1
USER majoral
F2 - Acquisition Parameters
Date_ 20110515
Time 2.24
INSTRUM spect
PROBHD 5 mm BBO BB/2H
PULPROG 1s_zg60dc
TD 32768
SOLVENT CDCl3
NS 26000
DS 4
SWH 25125.629 Hz
FIDRES 0.766773 Hz
AQ 0.6521332 sec
RG 36780.8
DW 19.900 usec
DE 10.00 usec
TE 298.0 K
D1 1.00000000 sec
d11 0.03000000 sec
MCREST 0.00000000 sec
MCWRK 0.01500000 sec
======== CHANNEL f1 ========
NUC1 13C
P1 7.30 usec
PL1 0.00 dB
SFO1 100.6233333 MHz
======== CHANNEL f2 ========
CPDPRG2 waltz16
NUC2 1H
PCPD2 92.00 usec
PL2 -1.00 dB
PL12 17.94 dB
SFO2 400.1318006 MHz
F2 - Processing parameters
SI 65536
SF 100.6127690 MHz
WDW EM
SSB 0
LB 1.00 Hz
GB 0
PC 0.30
220 200 180 160 140 120 100 80 60 40 20 0 ppm

^{13}C JMOD NMR of Compound 2

^{13}C

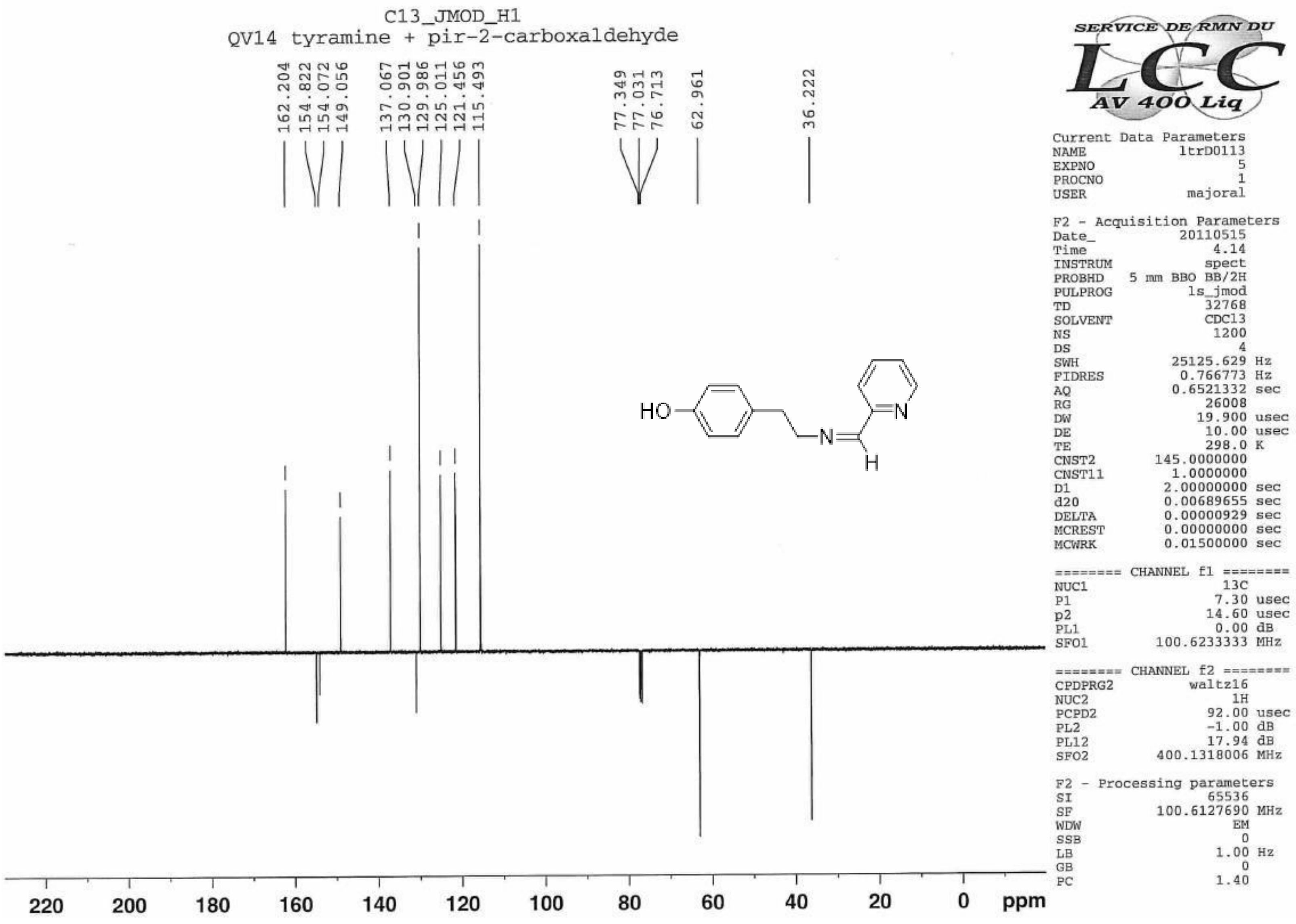
C13_JMOD_H1
QV14 tyramine + pir-2-carboxaldehyde
162.204
154.822
154.072
149.056
137.067
130.901
129.986
125.011
121.456
115.493
77.349
77.031
76.713
62.961
36.222
HO
N
N
H
SERVICE DE RMN DU
LCC
AV 400 Liq
Current Data Parameters
NAME 1trD0113
EXPNO 5
PROCNO 1
USER majoral
F2 - Acquisition Parameters
Date_ 20110515
Time 4.14
INSTRUM spect
PROBHD 5 mm BBO BB/2H
PULPROG ls_jmod
TD 32768
SOLVENT CDCl3
NS 1200
DS 4
SWH 25125.629 Hz
FIDRES 0.766773 Hz
AQ 0.6521332 sec
RG 26008
DW 19.900 usec
DE 10.00 usec
TE 298.0 K
CNST2 145.0000000
CNST11 1.0000000
D1 2.00000000 sec
d20 0.00689655 sec
DELTA 0.00000929 sec
MCREST 0.00000000 sec
MCWRK 0.01500000 sec
======== CHANNEL f1 ========
NUC1 13C
P1 7.30 usec
p2 14.60 usec
PL1 0.00 dB
SFO1 100.6233333 MHz
======== CHANNEL f2 ========
CPDPRG2 waltz16
NUC2 1H
PCPD2 92.00 usec
PL2 -1.00 dB
PL12 17.94 dB
SFO2 400.1318006 MHz
F2 - Processing parameters
SI 65536
SF 100.6127690 MHz
WDW EM
SSB 0
LB 1.00 Hz
GB 0
PC 1.40
220
200
180
160
140
120
100
80
60
40
20
0
ppm

^{13}C DEPT NMR of Compound 2

^{13}C

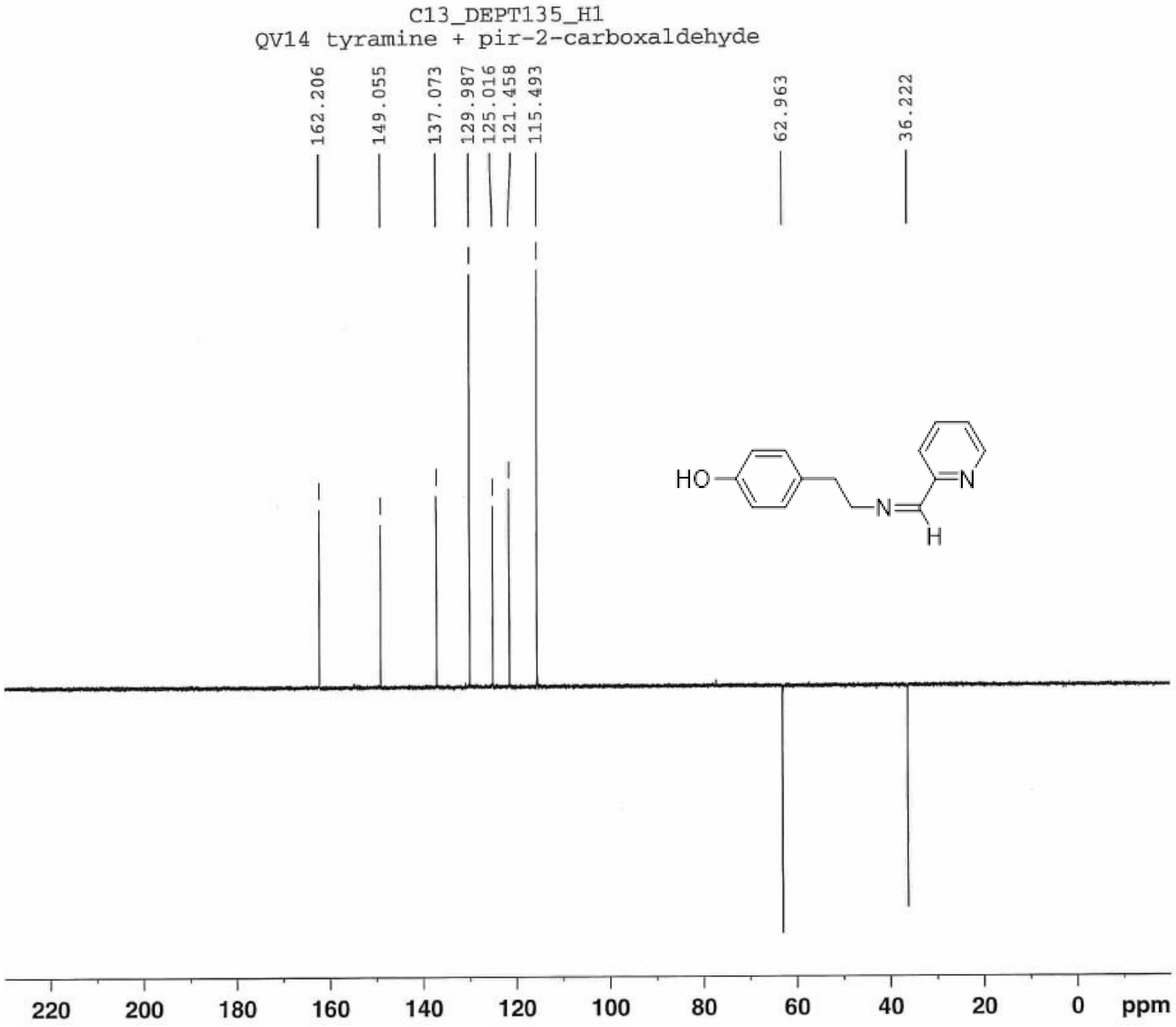

Current Data Parameters
NAME ltrD0113
EXPNO 4
PROCNO 1
USER majoral

F2 - Acquisition Parameters
Date_ 20110515
Time 3.19
INSTRUM spect
PROBHD 5 mm BBO BB/2H
PULPROG ls_dept135
TD 32768
SOLVENT CDCl3
NS 1200
DS 4
SWH 25125.629 Hz
FIDRES 0.766773 Hz
AQ 0.6521332 sec
RG 29193
DW 19.900 usec
DE 10.00 usec
TE 298.0 K
CNST2 145.0000000
D1 2.00000000 sec
d2 0.00344828 sec
d12 0.00002000 sec
DELTA 0.00000929 sec
MCREST 0.00000000 sec
MCWRK 0.01500000 sec

======== CHANNEL f1 ========
NUC1 13C
P1 7.30 usec
p2 14.60 usec
PL1 0.00 dB
SFO1 100.6233333 MHz

======== CHANNEL f2 ========
CPDPRG2 waltz16
NUC2 1H
P3 10.40 usec
p4 20.80 usec
PCPD2 92.00 usec
PL2 -1.00 dB
PL12 17.94 dB
SFO2 400.1318006 MHz

F2 - Processing parameters
SI 65536
SF 100.6127690 MHz
WDW EM
SSB 0
LB 1.00 Hz
GB 0
PC 1.40

^{13}C HMBC NMR of Compound 2

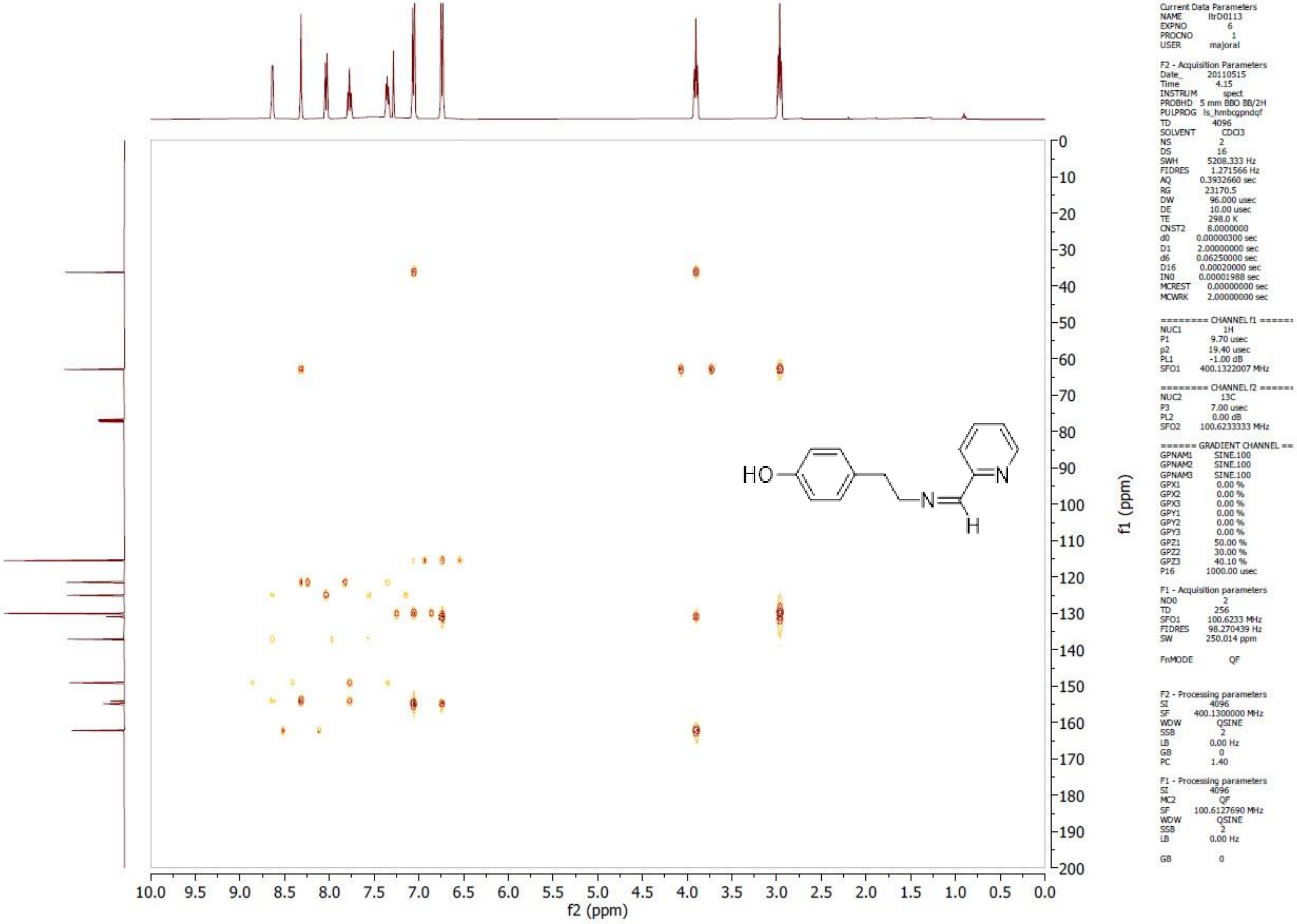

C HMQC NMR of Compound 2

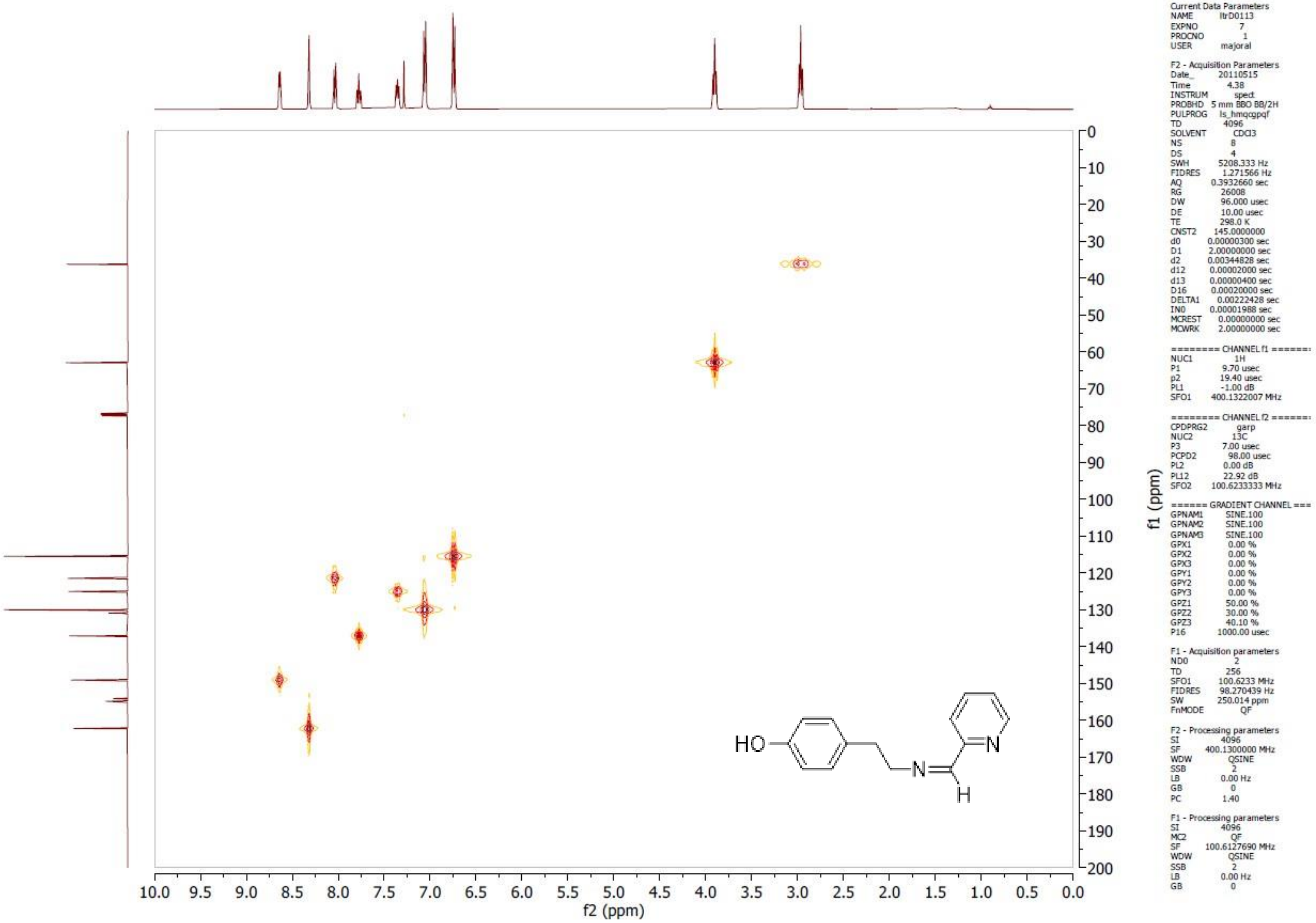

^{1}H NMR of Compound 2a

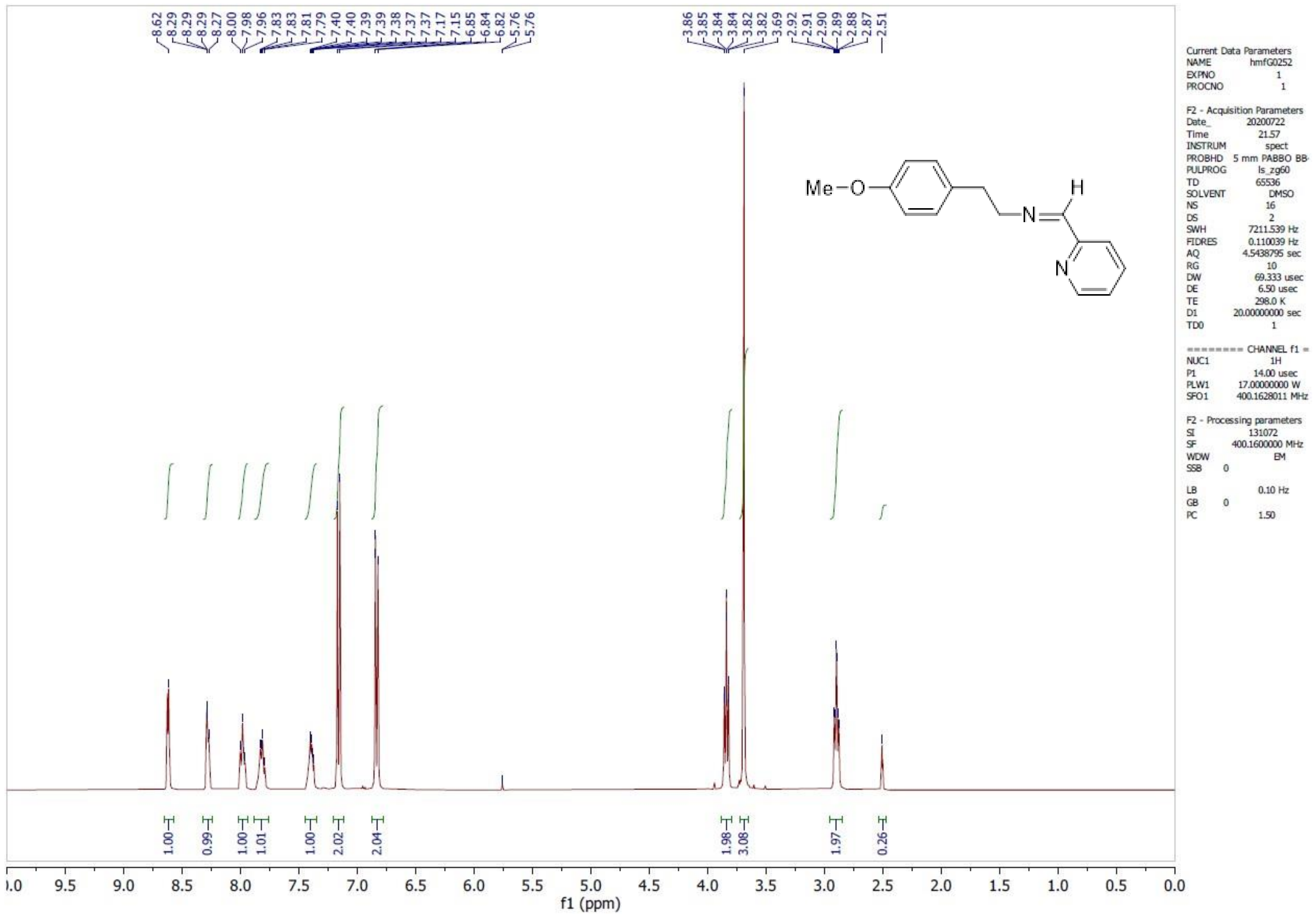

13

C {1H} NMR of Compound 2a

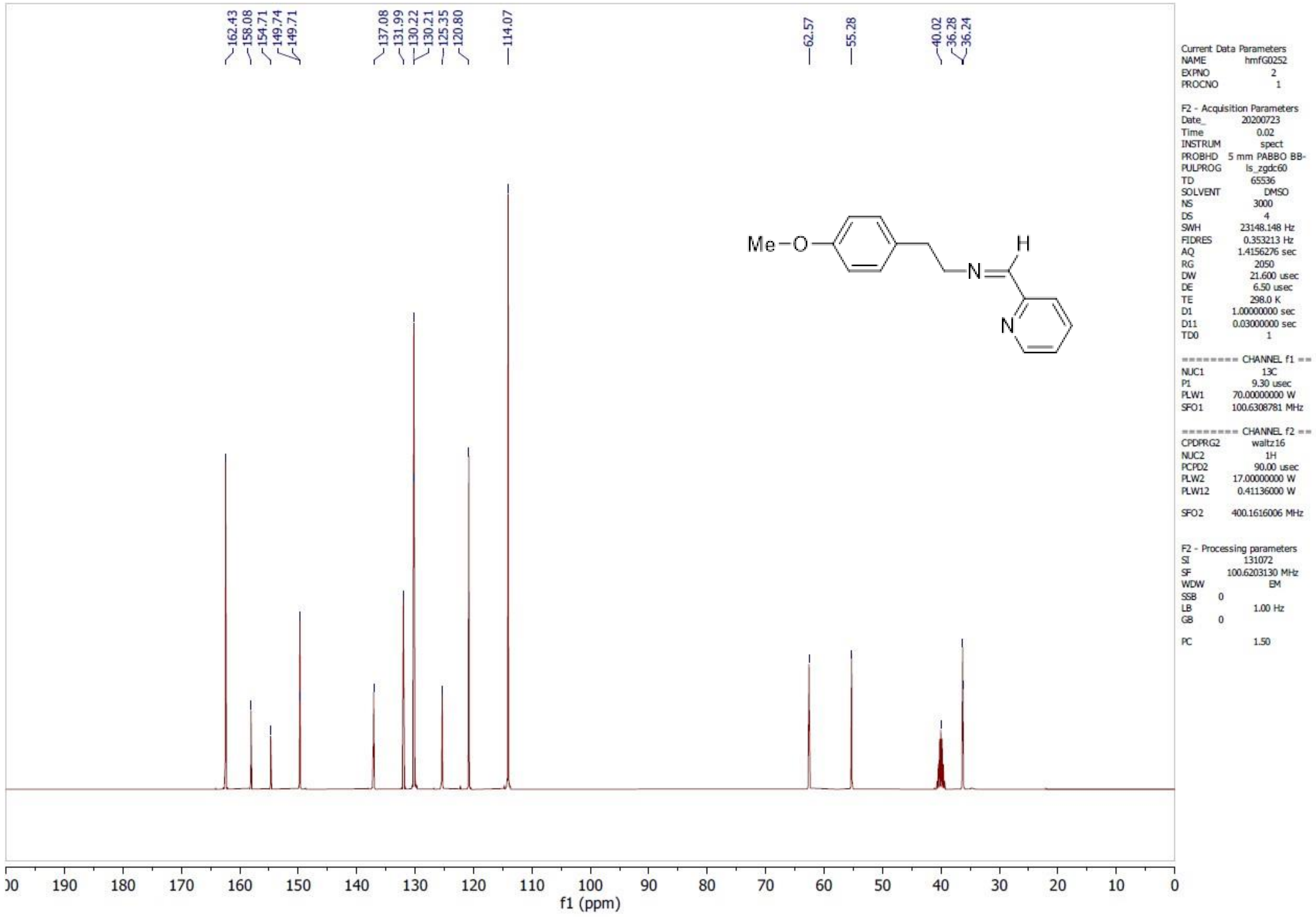

[13] C JMOD NMR of Compound 2a

13

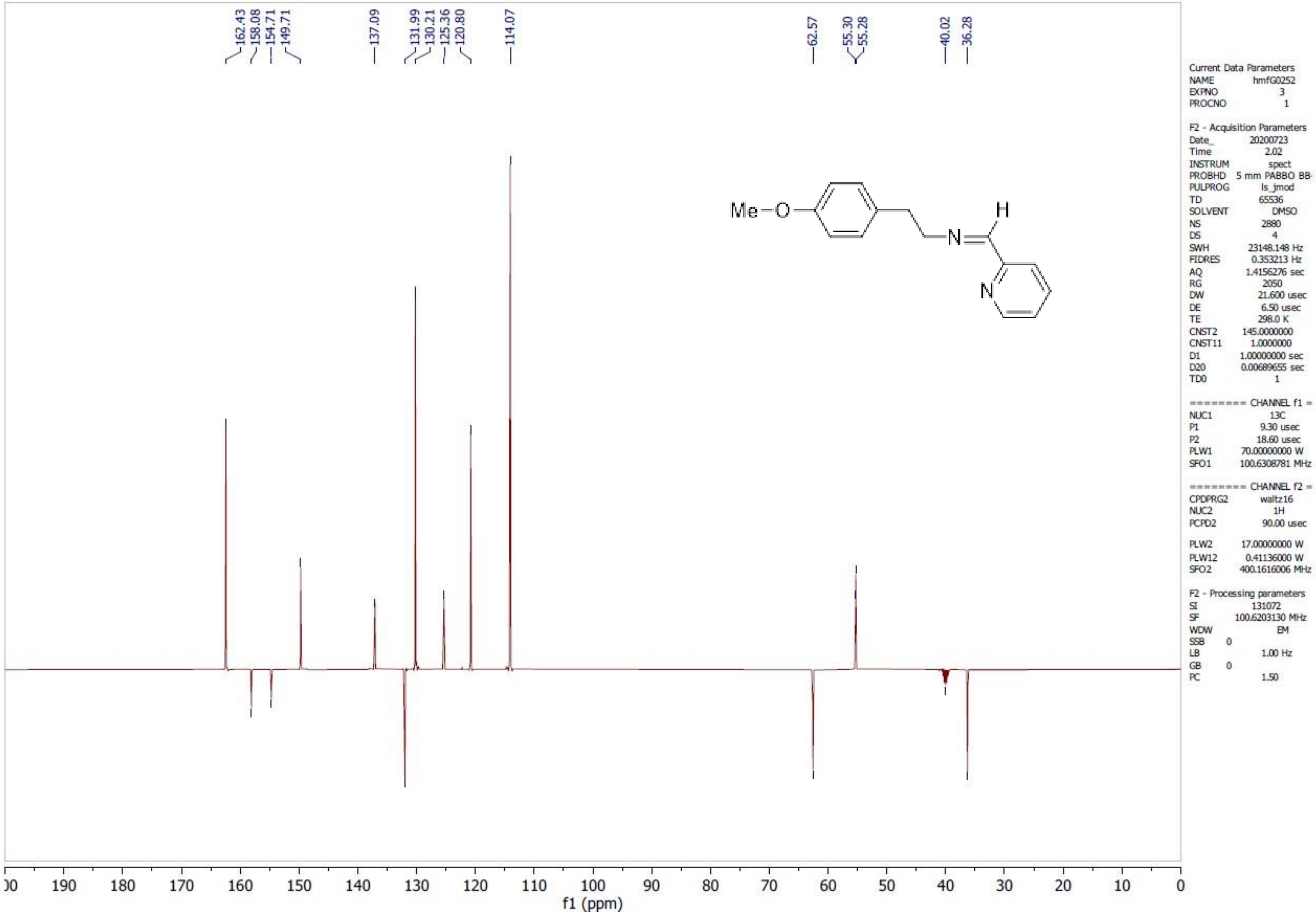
162.43
158.08
154.71
149.71
137.09
131.99
130.21
125.36
120.80
114.07
62.57
55.30
55.28
40.02
36.28
Me−O
N
H
N
Current Data Parameters
NAME hmfG0252
EXPNO 3
PROCNO 1
F2 - Acquisition Parameters
Date_ 20200723
Time 2.02
INSTRUM spect
PROBHD 5 mm PABBO BB-
PULPROG ls_jmod
TD 65536
SOLVENT DMSO
NS 2880
DS 4
SWH 23148.148 Hz
FIDRES 0.353213 Hz
AQ 1.4156276 sec
RG 2050
DW 21.600 usec
DE 6.50 usec
TE 298.0 K
CNST2 145.0000000
CNST11 1.0000000
D1 1.00000000 sec
D20 0.00689655 sec
TD0 1
======== CHANNEL f1 =
NUC1 13C
P1 9.30 usec
P2 18.60 usec
PLW1 70.00000000 W
SFO1 100.6308781 MHz
======== CHANNEL f2 =
CPDPRG2 waltz16
NUC2 1H
PCPD2 90.00 usec
PLW2 17.00000000 W
PLW12 0.41136000 W
SFO2 400.1616006 MHz
F2 - Processing parameters
SI 131072
SF 100.6203130 MHz
WDW EM
SSB 0
LB 1.00 Hz
GB 0
PC 1.50
f1 (ppm)

C HMQC NMR of Compound 2a

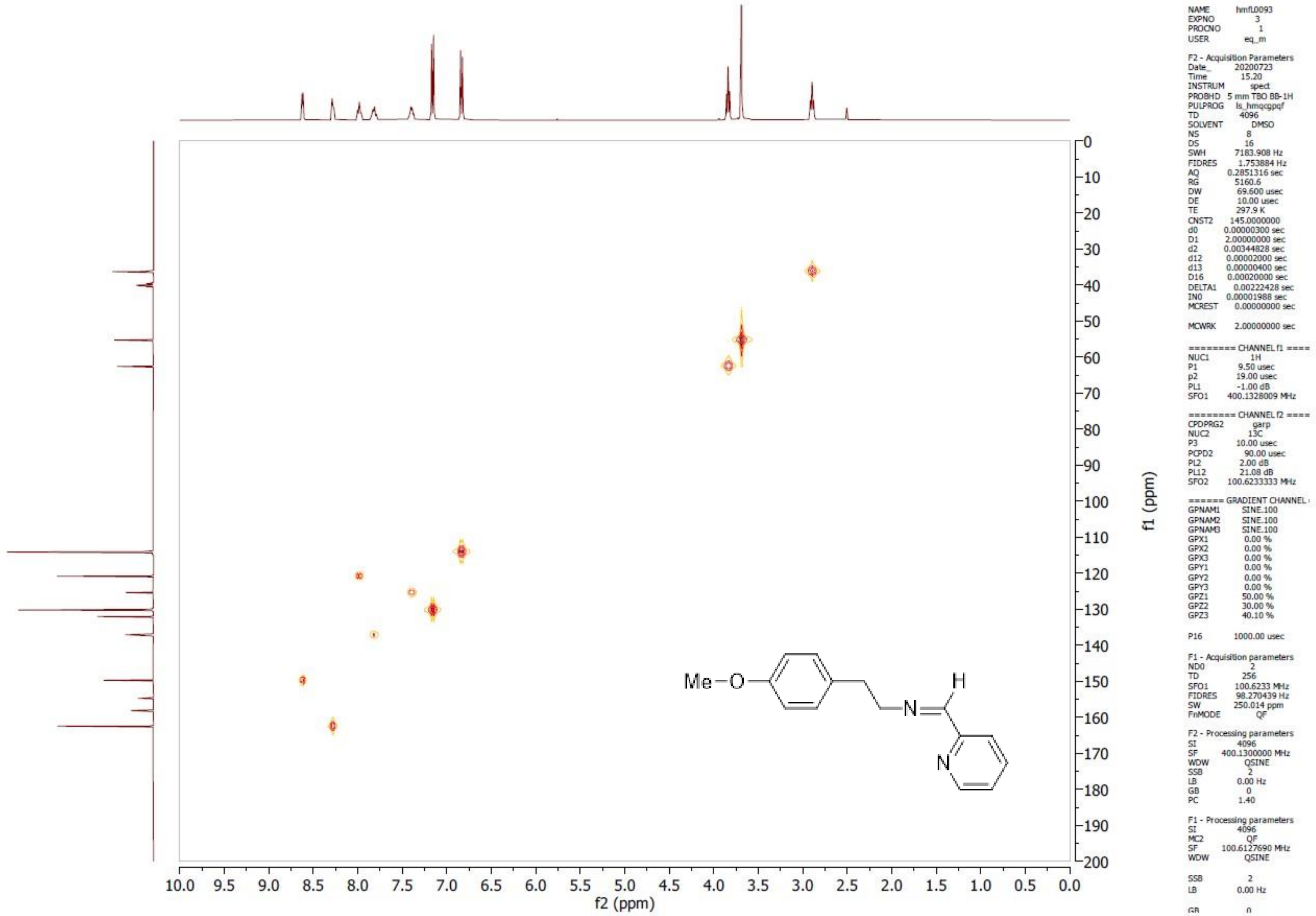

13

^{1}H NMR of Compound 2a-Pd

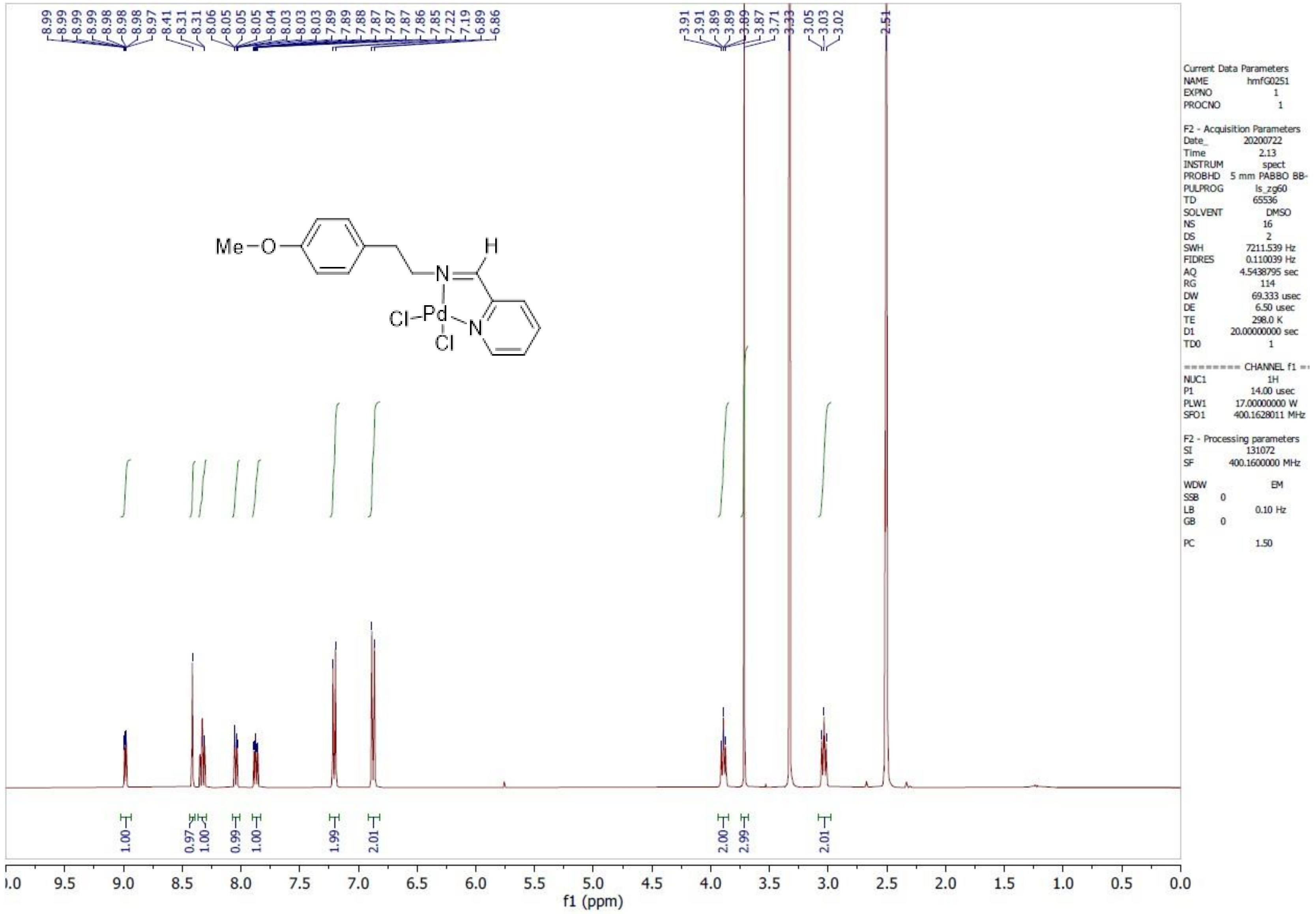

C {¹H} NMR of Compound 2a-Pd

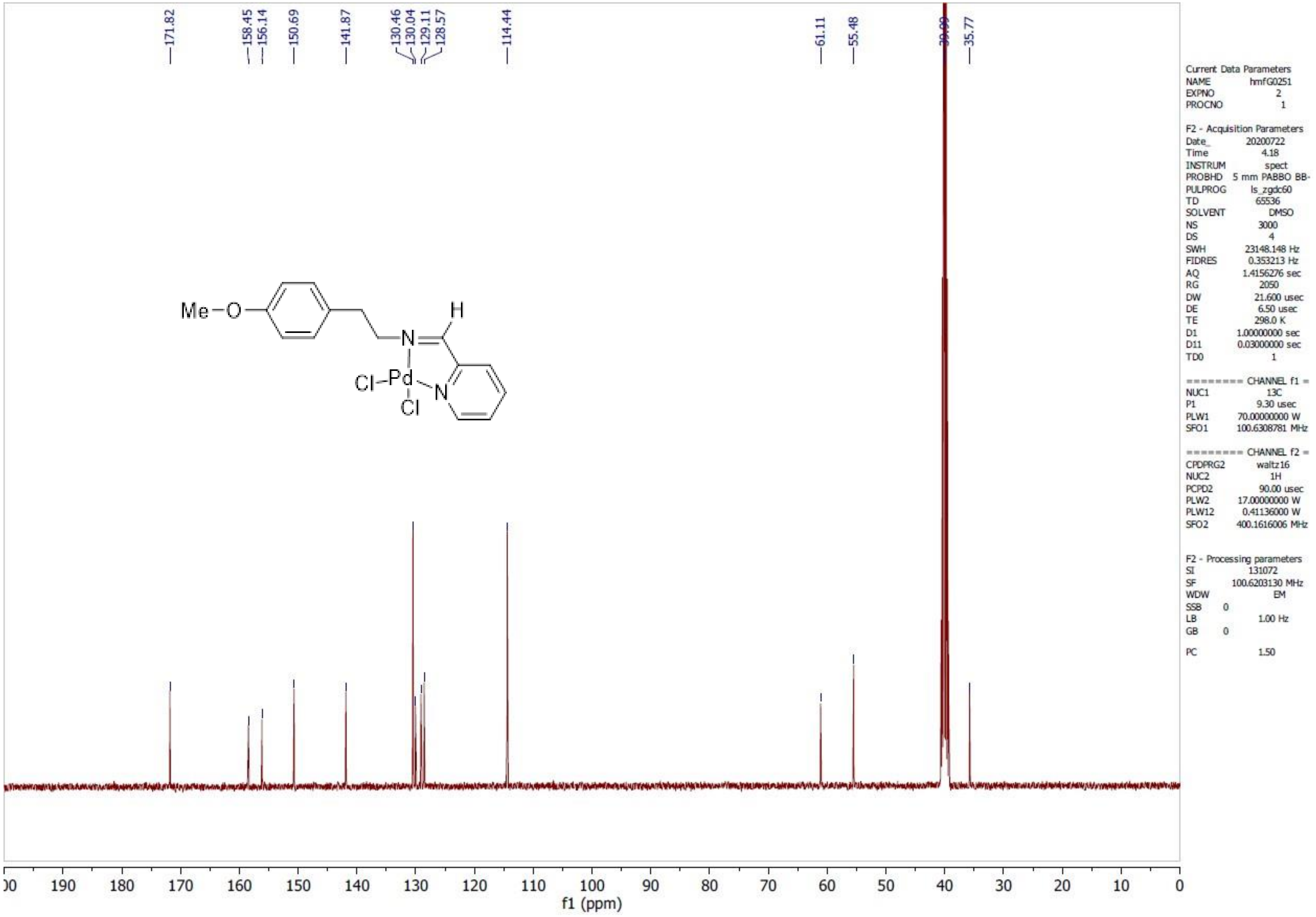

^{13}C HMQC NMR of Compound 2a-Pd

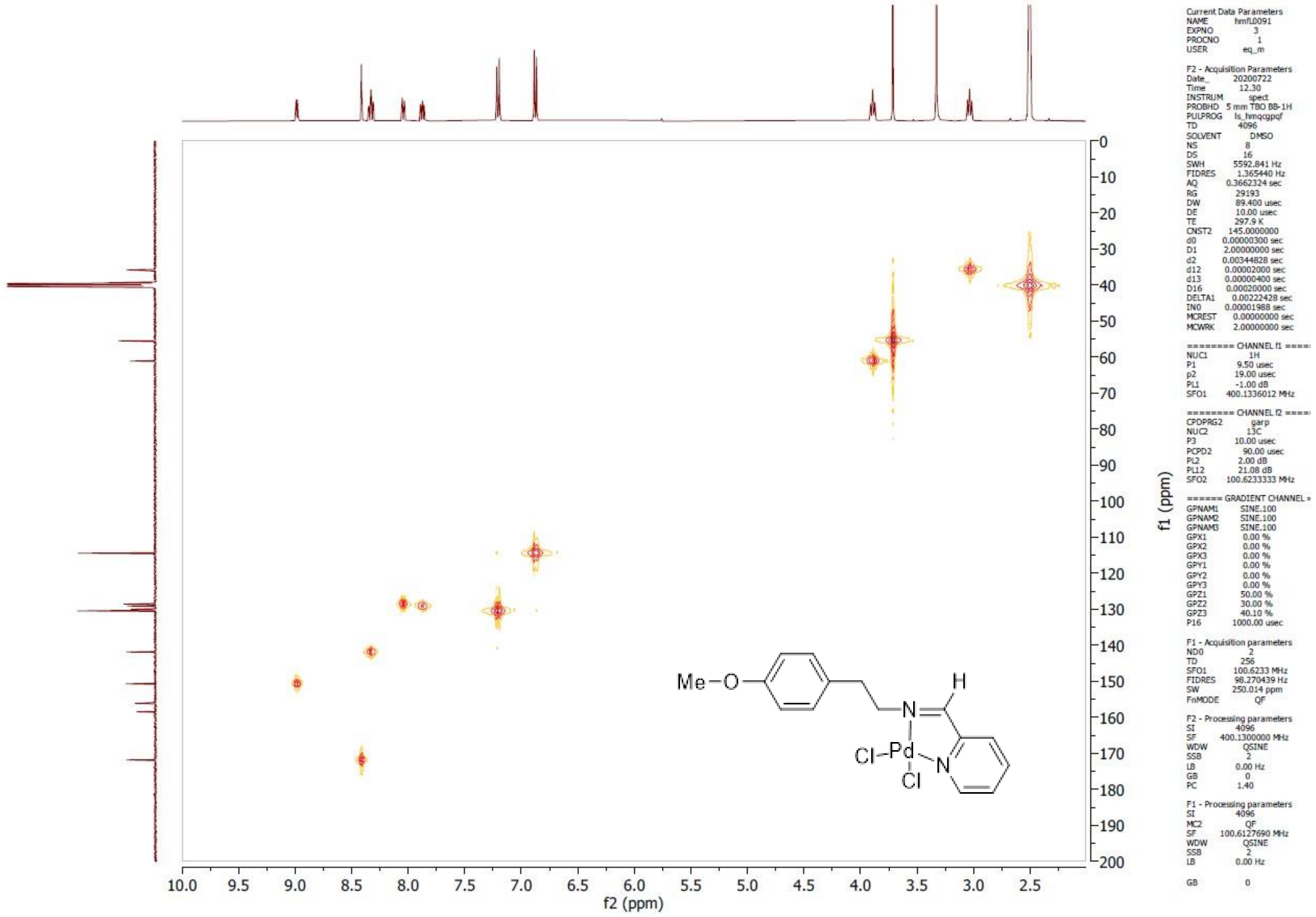

^{1}H NMR of Compound 3

1

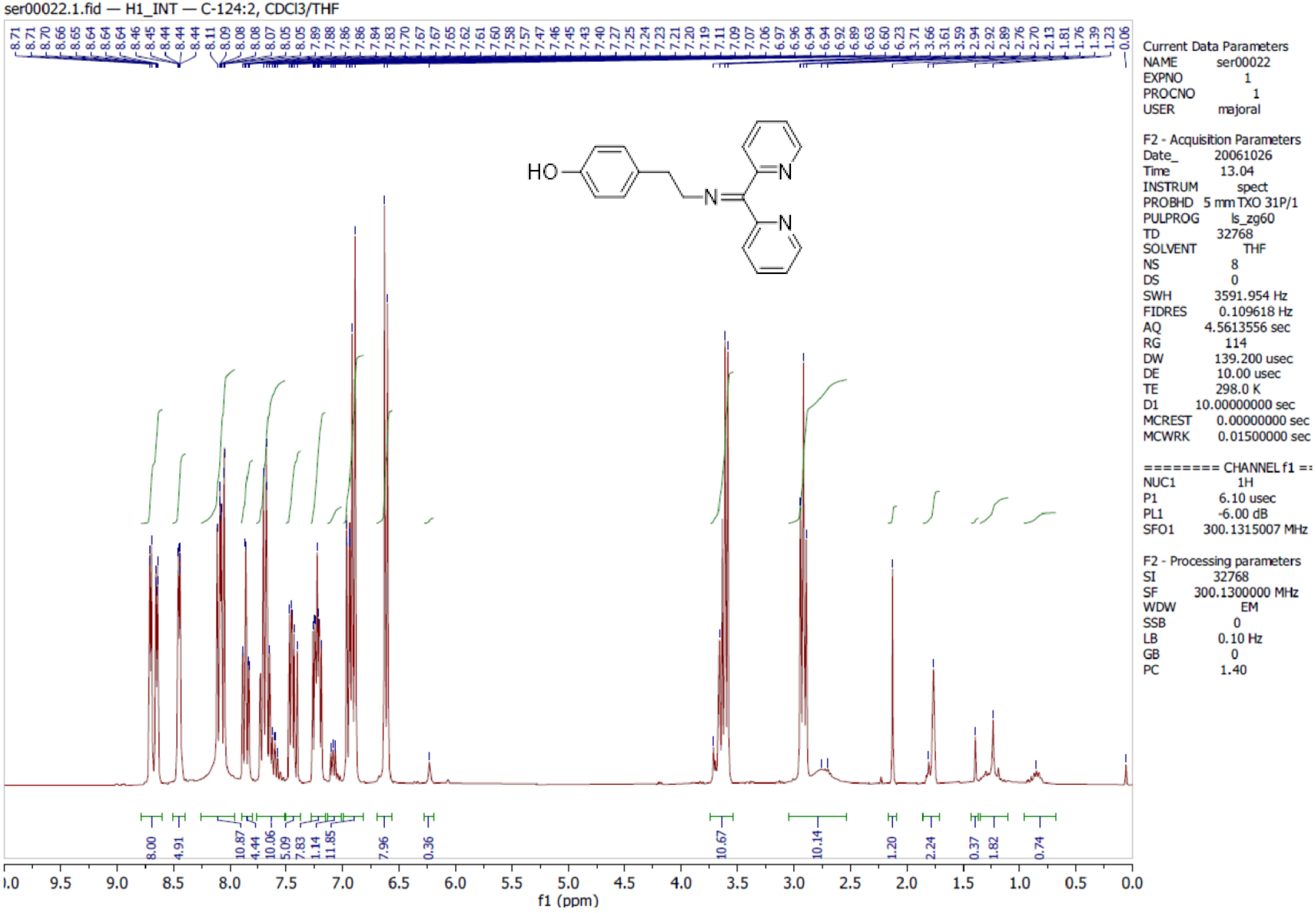
ser00022.1.fid — H1_INT — C-124:2, CDCl3/THF
HO
N
N
N
Current Data Parameters
NAME ser00022
EXPNO 1
PROCNO 1
USER majoral
F2 - Acquisition Parameters
Date_ 20061026
Time 13.04
INSTRUM spect
PROBHD 5 mm TXO 31P/1
PULPROG ls_zg60
TD 32768
SOLVENT THF
NS 8
DS 0
SWH 3591.954 Hz
FIDRES 0.109618 Hz
AQ 4.5613556 sec
RG 114
DW 139.200 usec
DE 10.00 usec
TE 298.0 K
D1 10.00000000 sec
MCREST 0.00000000 sec
MCWRK 0.01500000 sec
======== CHANNEL f1 ==
NUC1 1H
P1 6.10 usec
PL1 -6.00 dB
SFO1 300.1315007 MHz
F2 - Processing parameters
SI 32768
SF 300.1300000 MHz
WDW EM
SSB 0
LB 0.10 Hz
GB 0
PC 1.40
f1 (ppm)

^{1}H COSY NMR of Compound 3

1

H1_COSY_GP_QF
C-124:2, CDCl3/THF

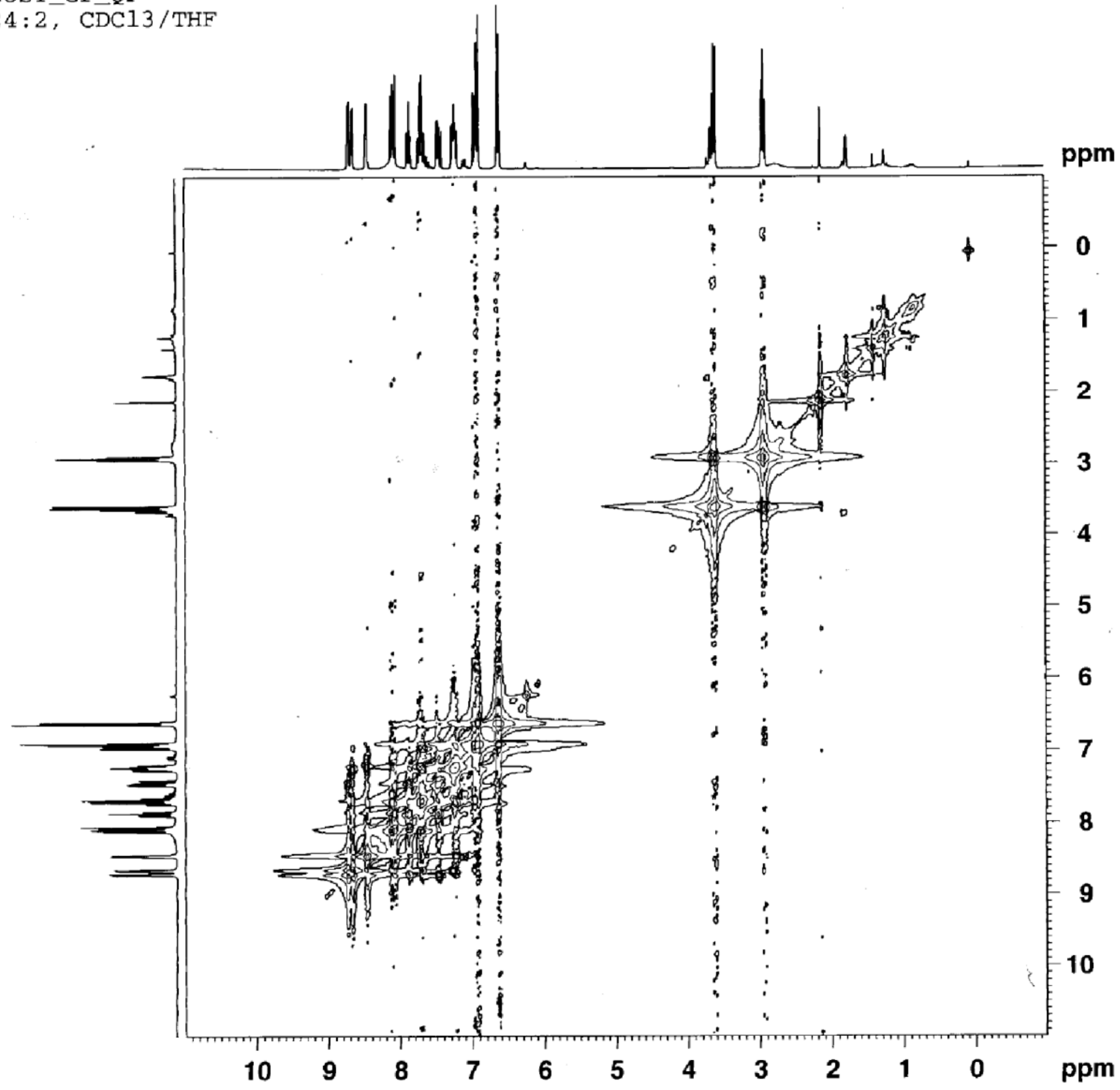

SERVICE DE RMN DU LCC AV 300

```
Current Data Parameters
NAME                ser00022
EXPNO                      5
PROCNO                     1
USER                 majoral

F2 - Acquisition Parameters
Date_               20061026
Time                   13.52
INSTRUM                spect
PROBHD   5 mm TXO 31P/1
PULPROG        ls_cosygpqf
TD                      4096
SOLVENT                  THF
NS                         2
DS                         0
SWH                 3591.954 Hz
FIDRES              0.876942 Hz
AQ                 0.5702132 sec
RG                       128
DW                   139.200 usec
DE                     10.00 usec
TE                     298.0 K
d0                0.00000300 sec
D1                2.00000000 sec
d13               0.00000400 sec
D16               0.00020000 sec
IN0               0.00027840 sec
MCREST            0.00000000 sec
MCWRK             2.00000000 sec

======== CHANNEL f1 ========
NUC1                      1H
P0                      6.10 usec
P1                      6.10 usec
PL1                    -6.00 dB
SFO1             300.1315007 MHz

====== GRADIENT CHANNEL =====
GPNAM1              SINE.100
GPNAM2              SINE.100
GPX1                    0.00 %
GPX2                    0.00 %
GPY1                    0.00 %
GPY2                    0.00 %
GPZ1                   10.00 %
GPZ2                   10.00 %
P16                  1000.00 usec

F1 - Acquisition parameters
ND0                        1
TD                       256
SFO1                300.1315 MHz
FIDRES             14.031071 Hz
SW                    11.968 ppm
FnMODE                    QF

F2 - Processing parameters
SI                      4096
SF               300.1300000 MHz
WDW                    QSINE
SSB                        2
LB                      0.00 Hz
GB                         0
PC                      1.40

F1 - Processing parameters
SI                      4096
MC2                       QF
SF               300.1300000 MHz
WDW                    QSINE
SSB                        2
LB                      0.00 Hz
GB                         0
```

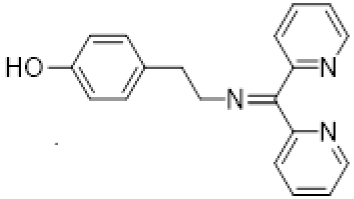

^{1}H NOESY NMR of Compound 3

1

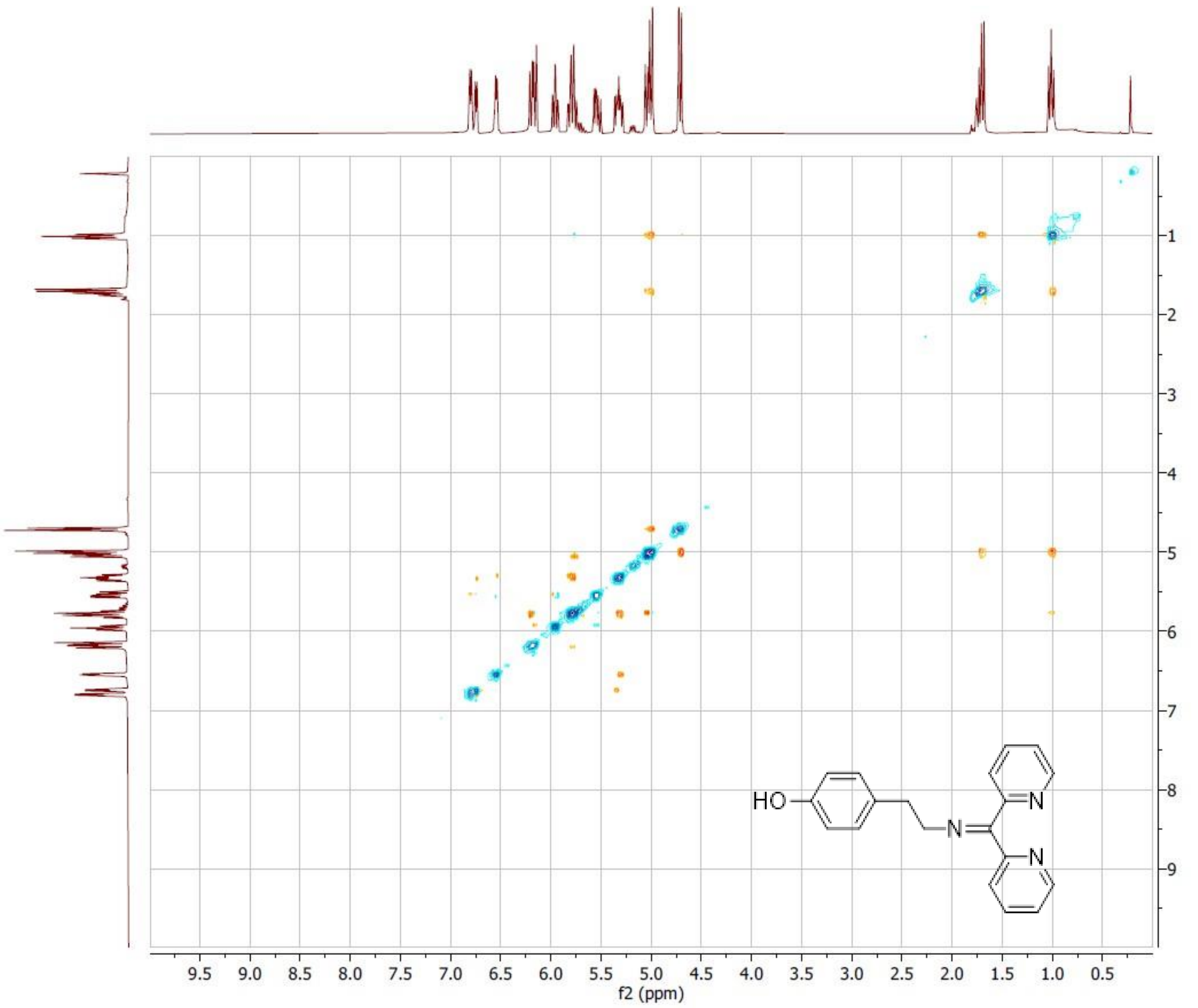

Current Data Parameters
NAME ser00024
EXPNO 3
PROCNO 1
USER majoral

F2 - Acquisition Parameters
Date_ 20061028
Time 10.14
INSTRUM spect
PROBHD 5 mm TXO 31P/1
PULPROG ls_noesyphrv
TD 4096
SOLVENT THF
NS 8
DS 0
SWH 2997.602 Hz
FIDRES 0.731836 Hz
AQ 0.6832628 sec
RG 128
DW 166.800 usec
DE 10.00 usec
TE 298.0 K
d0 0.00015903 sec
D1 1.00000000 sec
D8 1.00000000 sec
IN0 0.00033360 sec
MCREST 0.00000000 sec
MCWRK 1.00000000 sec

======== CHANNEL f1 =
NUC1 1H
P1 6.10 usec
PL1 -6.00 dB
SFO1 300.1315007 MHz

F1 - Acquisition parameters
ND0 1
TD 248
SFO1 300.1315 MHz
FIDRES 12.087105 Hz
SW 9.988 ppm
FnMODE TPPI

F2 - Processing parameters
SI 4096
SF 300.1300000 MHz
WDW QSINE
SSB 2
LB 0.00 Hz
GB 0
PC 1.40

F1 - Processing parameters
SI 4096
MC2 TPPI
SF 300.1300000 MHz
WDW QSINE
SSB 2
LB 0.00 Hz
GB 0

^{13}C {^{1}H} NMR of Compound 3

^{13}C

ser00022.2.fid — C13_DECOUPLE_H1 — C-124:2, CDCl3/THF

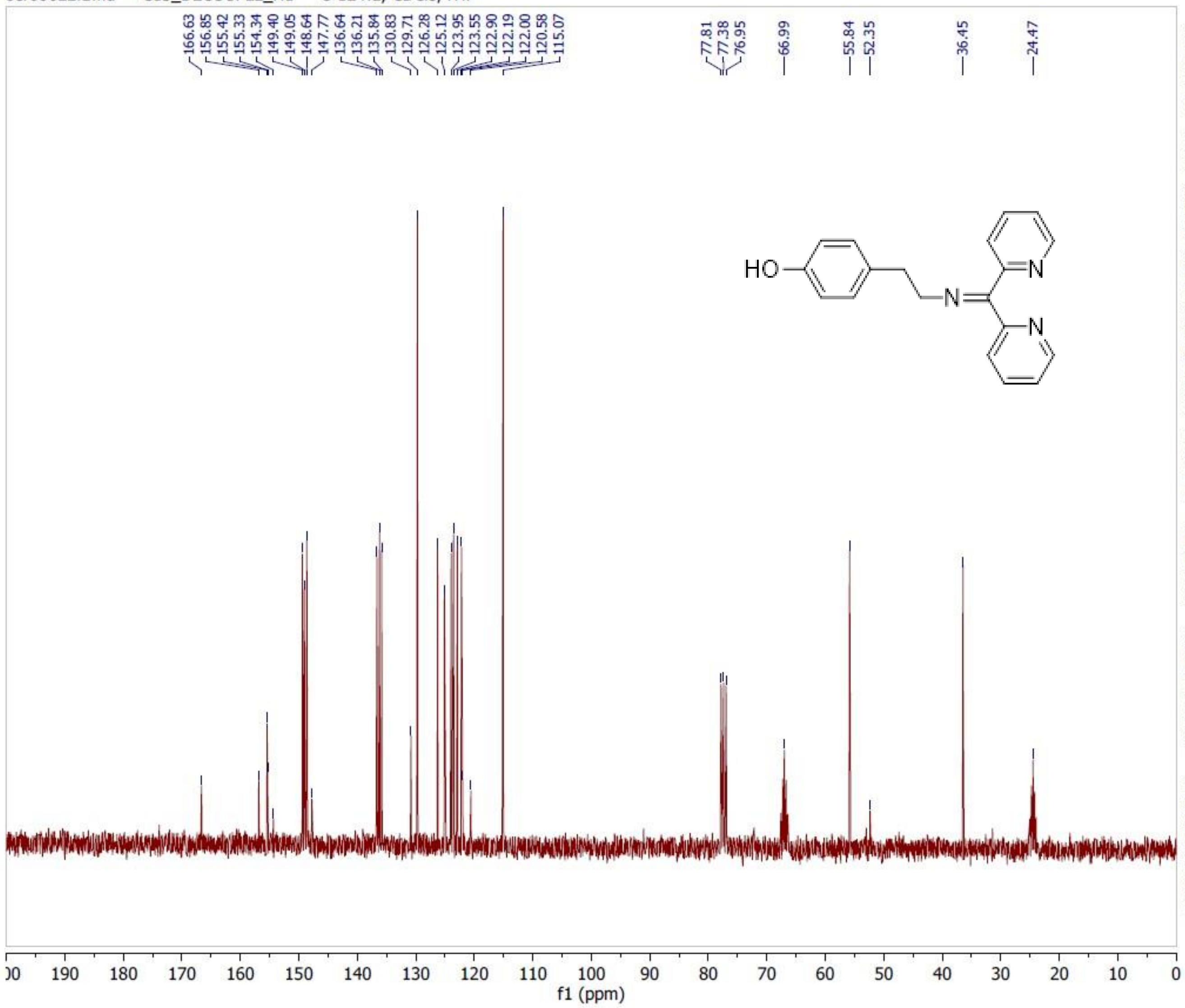

Current Data Parameters
NAME ser00022
EXPNO 2
PROCNO 1
USER majoral

F2 - Acquisition Parameters
Date_ 20061026
Time 13.16
INSTRUM spect
PROBHD 5 mm TXO 31P/1
PULPROG ls_zg60dc
TD 32768
SOLVENT THF
NS 280
DS 0
SWH 18832.393 Hz
FIDRES 0.574719 Hz
AQ 0.8700404 sec
RG 26008
DW 26.550 usec
DE 10.00 usec
TE 297.9 K
D1 1.00000000 sec
d11 0.03000000 sec
MCREST 0.00000000 sec
MCWRK 0.01500000 sec

======== CHANNEL f1 ========
NUC1 13C
P1 7.20 usec
PL1 -6.00 dB
SFO1 75.4756731 MHz

======== CHANNEL f2 ========
CPDPRG2 waltz16
NUC2 1H
PCPD2 88.00 usec
PL2 -6.00 dB
PL12 17.62 dB

SFO2 300.1312005 MHz

F2 - Processing parameters
SI 65536
SF 75.4677490 MHz
WDW EM
SSB 0
LB 1.00 Hz
GB 0
PC 1.40

^{13}C JMOD NMR of Compound 3

^{13}C

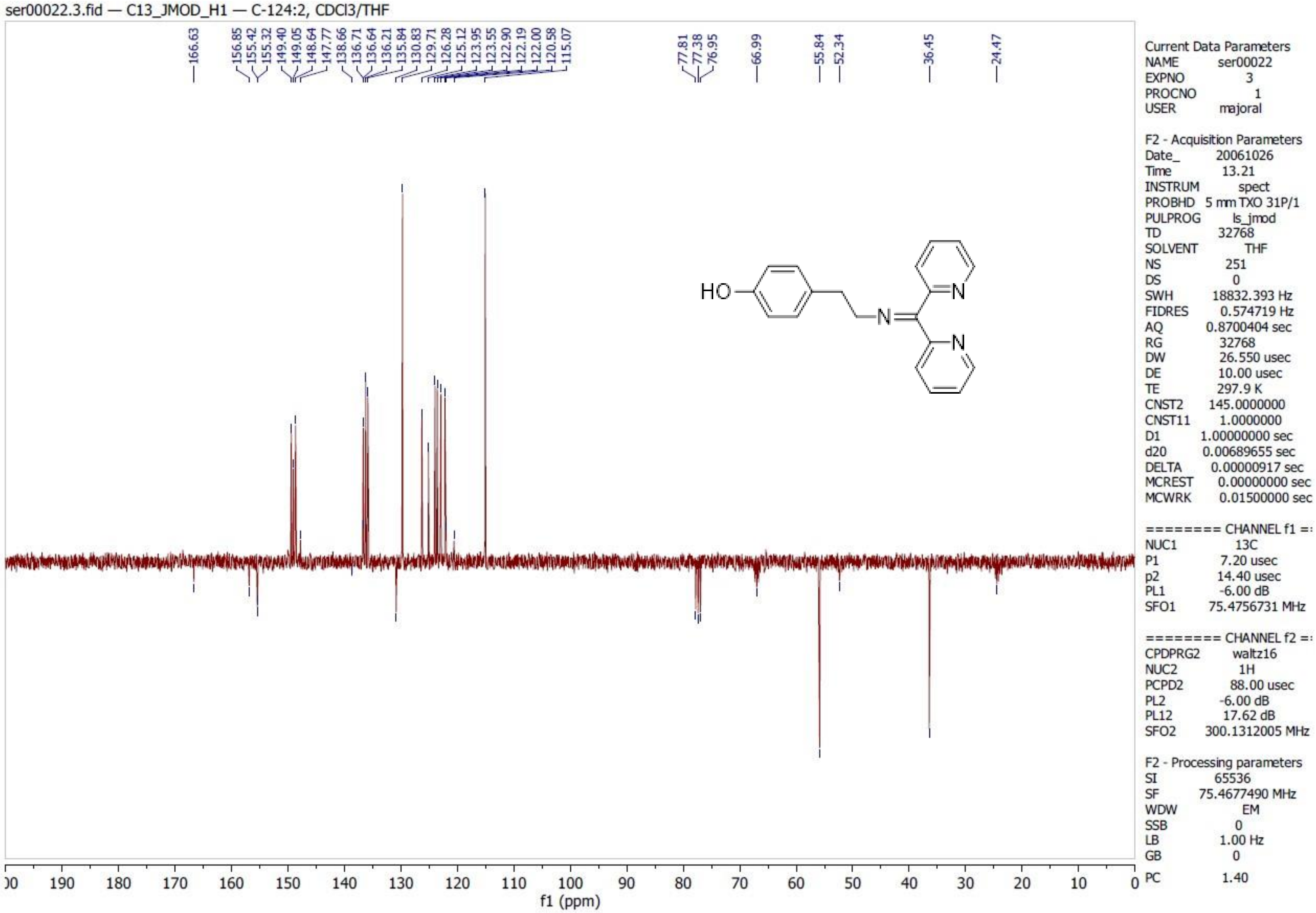
ser00022.3.fid — C13_JMOD_H1 — C-124:2, CDCl3/THF
166.63
156.85
155.42
155.32
149.40
149.05
148.64
147.77
138.66
136.71
136.64
136.21
135.84
130.83
129.71
126.28
125.12
123.95
123.55
122.90
122.19
122.00
120.58
115.07
77.81
77.38
76.95
66.99
55.84
52.34
36.45
24.47
HO
N
N
N
Current Data Parameters
NAME ser00022
EXPNO 3
PROCNO 1
USER majoral
F2 - Acquisition Parameters
Date_ 20061026
Time 13.21
INSTRUM spect
PROBHD 5 mm TXO 31P/1
PULPROG ls_jmod
TD 32768
SOLVENT THF
NS 251
DS 0
SWH 18832.393 Hz
FIDRES 0.574719 Hz
AQ 0.8700404 sec
RG 32768
DW 26.550 usec
DE 10.00 usec
TE 297.9 K
CNST2 145.0000000
CNST11 1.0000000
D1 1.00000000 sec
d20 0.00689655 sec
DELTA 0.00000917 sec
MCREST 0.00000000 sec
MCWRK 0.01500000 sec
======== CHANNEL f1 ==
NUC1 13C
P1 7.20 usec
p2 14.40 usec
PL1 -6.00 dB
SFO1 75.4756731 MHz
======== CHANNEL f2 ==
CPDPRG2 waltz16
NUC2 1H
PCPD2 88.00 usec
PL2 -6.00 dB
PL12 17.62 dB
SFO2 300.1312005 MHz
F2 - Processing parameters
SI 65536
SF 75.4677490 MHz
WDW EM
SSB 0
LB 1.00 Hz
GB 0
PC 1.40
00 190 180 170 160 150 140 130 120 110 100 90 80 70 60 50 40 30 20 10 0
f1 (ppm)

^{13}C

HMQC NMR of Compound 3

$_{13}$C

ser00022.4.ser — C13_HMQC_GP_QF — C-124:2, CDCl3/THF

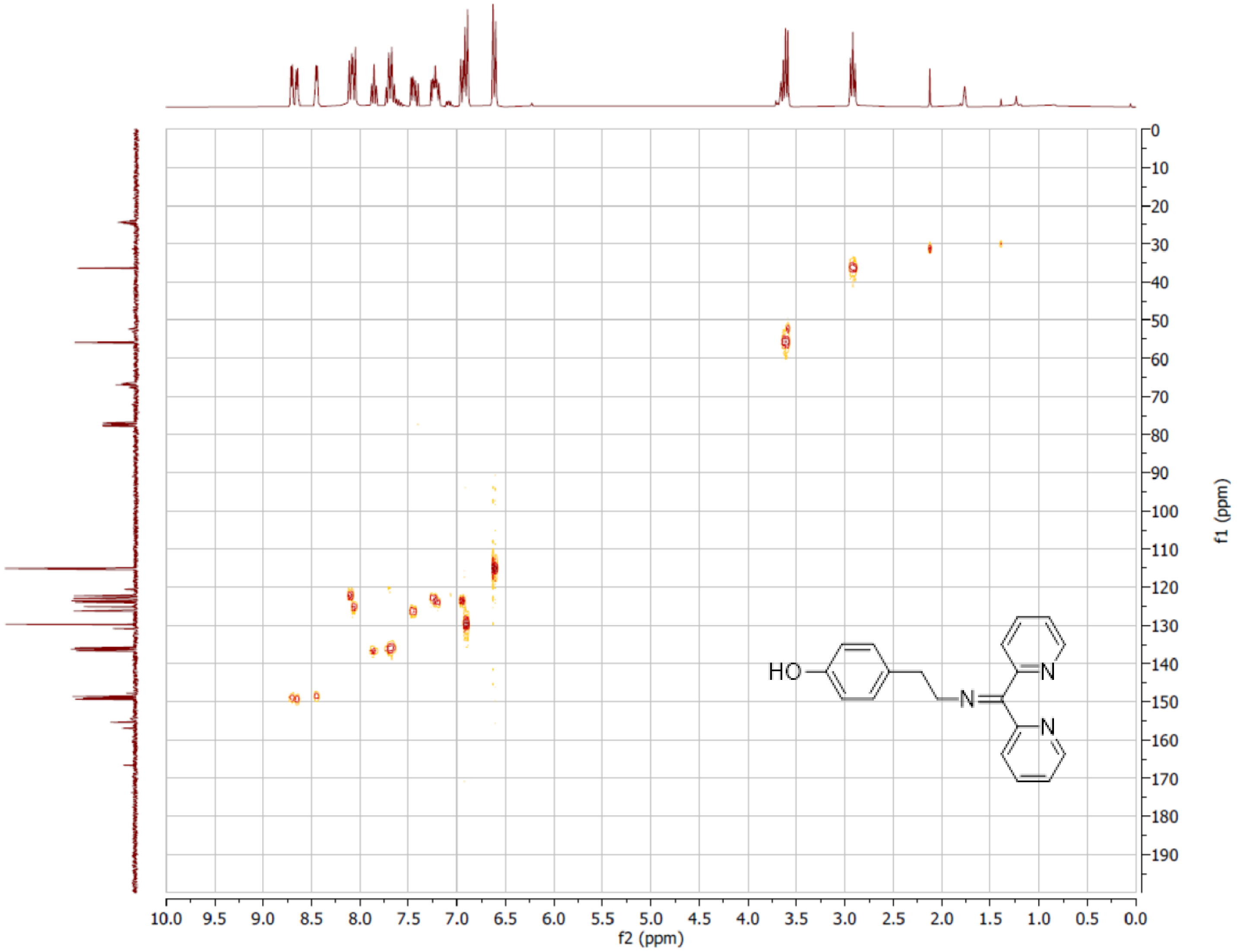

Current Data Parameters
NAME ser00022
EXPNO 4
PROCNO 1
USER majoral

F2 - Acquisition Parameters
Date_ 20061026
Time 13.29
INSTRUM spect
PROBHD 5 mm TXO 31P/1
PULPROG ls_hmqcgpqf
TD 4096
SOLVENT THF
NS 2
DS 0
SWH 3591.954 Hz
FIDRES 0.876942 Hz
AQ 0.5702132 sec
RG 128
DW 139.200 usec
DE 10.00 usec
TE 297.9 K
CNST2 145.0000000
d0 0.00000300 sec
D1 2.00000000 sec
d2 0.00344828 sec
d12 0.00002000 sec
d13 0.00000400 sec
D16 0.00020000 sec
DELTA1 0.00222428 sec
IN0 0.00002650 sec
MCREST 0.00000000 sec
MCWRK 2.00000000 sec

======== CHANNEL f1 =====
NUC1 1H
P1 6.10 usec
p2 12.20 usec
PL1 -6.00 dB
SFO1 300.1315007 MHz

======== CHANNEL f2 =====
CPDPRG2 garp
NUC2 13C
P3 6.50 usec
PCPD2 84.00 usec
PL2 -6.00 dB
PL12 16.00 dB
SFO2 75.4756731 MHz

====== GRADIENT CHANNEL =
GPNAM1 SINE.100
GPNAM2 SINE.100
GPNAM3 SINE.100
GPX1 0.00 %
GPX2 0.00 %
GPX3 0.00 %
GPY1 0.00 %
GPY2 0.00 %
GPY3 0.00 %
GPZ1 50.00 %
GPZ2 30.00 %
GPZ3 40.10 %
P16 1000.00 usec

F1 - Acquisition parameters
ND0 2
TD 256
SFO1 75.47567 MHz
FIDRES 73.702827 Hz
SW 249.987 ppm
FnMODE QF

F2 - Processing parameters
SI 4096
SF 300.1300000 MHz
WDW QSINE
SSB 2
LB 0.00 Hz
GB 0
PC 1.40

F1 - Processing parameters
SI 4096
MC2 QF
SF 75.4677490 MHz
WDW QSINE
SSB 2
LB 0.00 Hz
GB 0

$^{13}C\{^1H\}$ NMR of Compound 3

a

¹H NMR of Compound 3

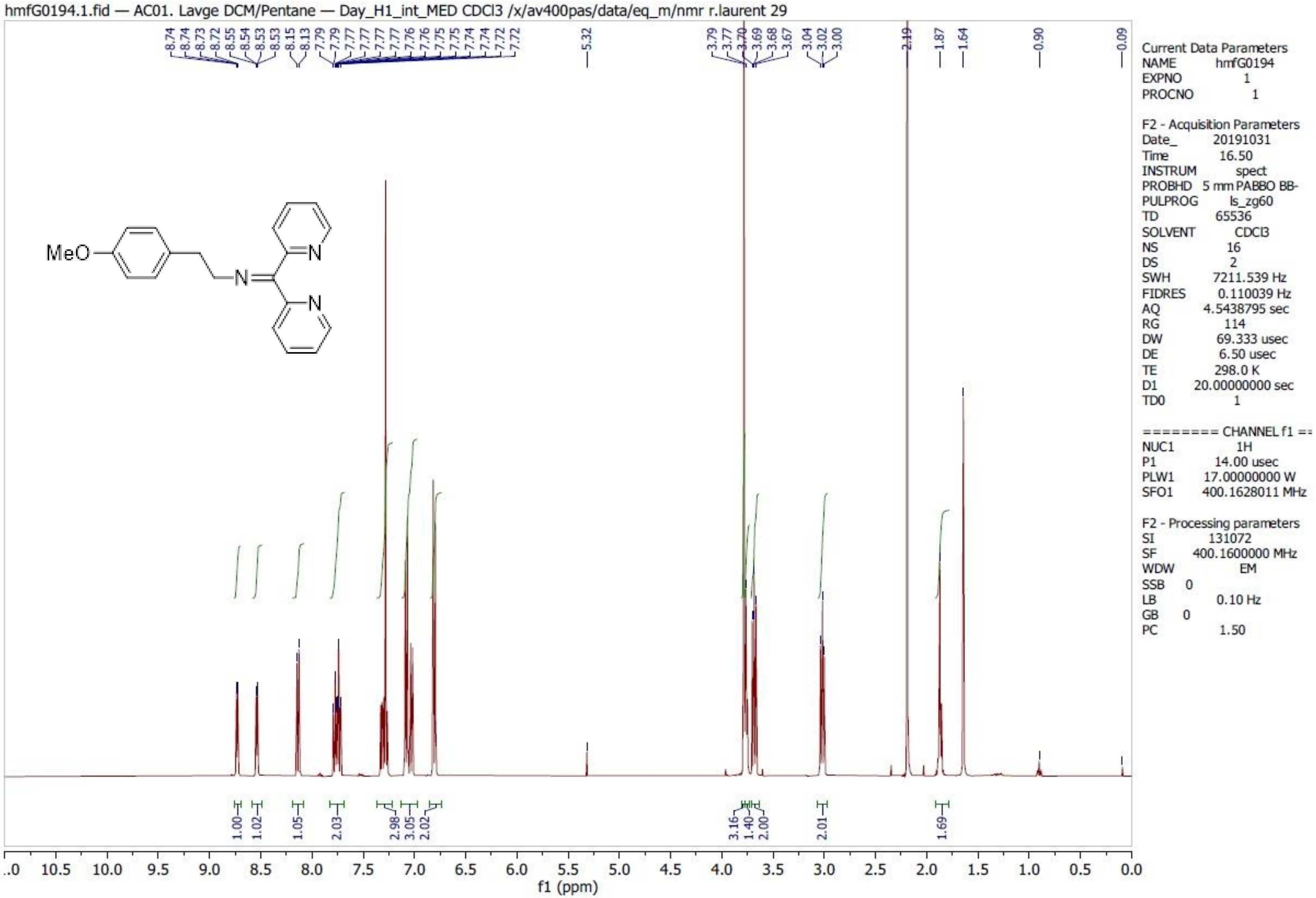

$^{13}C\{^{1}H\}$ NMR of Compound 3

a

^{1}H NMR of Compound 3

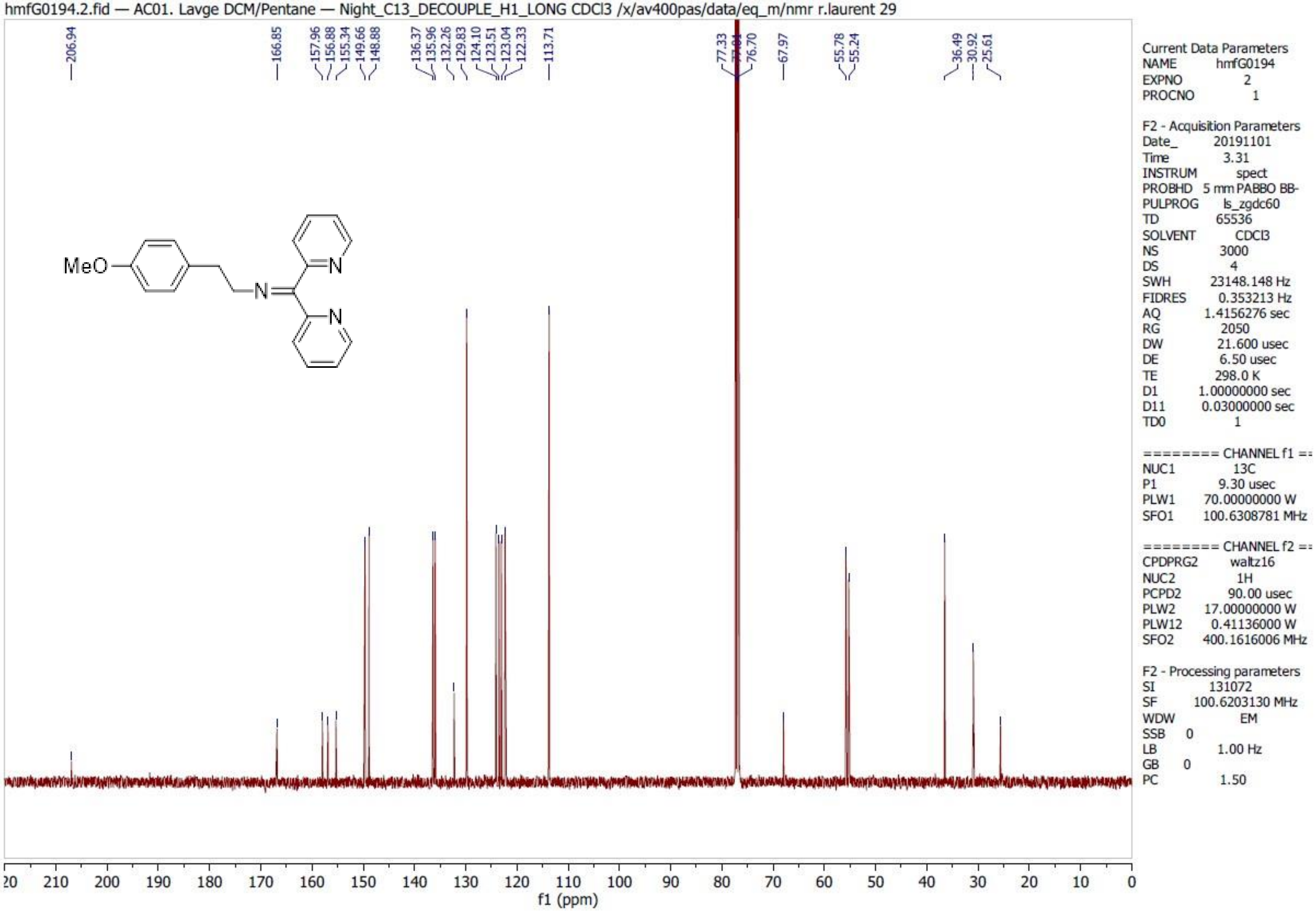

$^{13}C\{^{1}H\}$ NMR of Compound 3

-Pd

^{1}H NMR of Compound 3

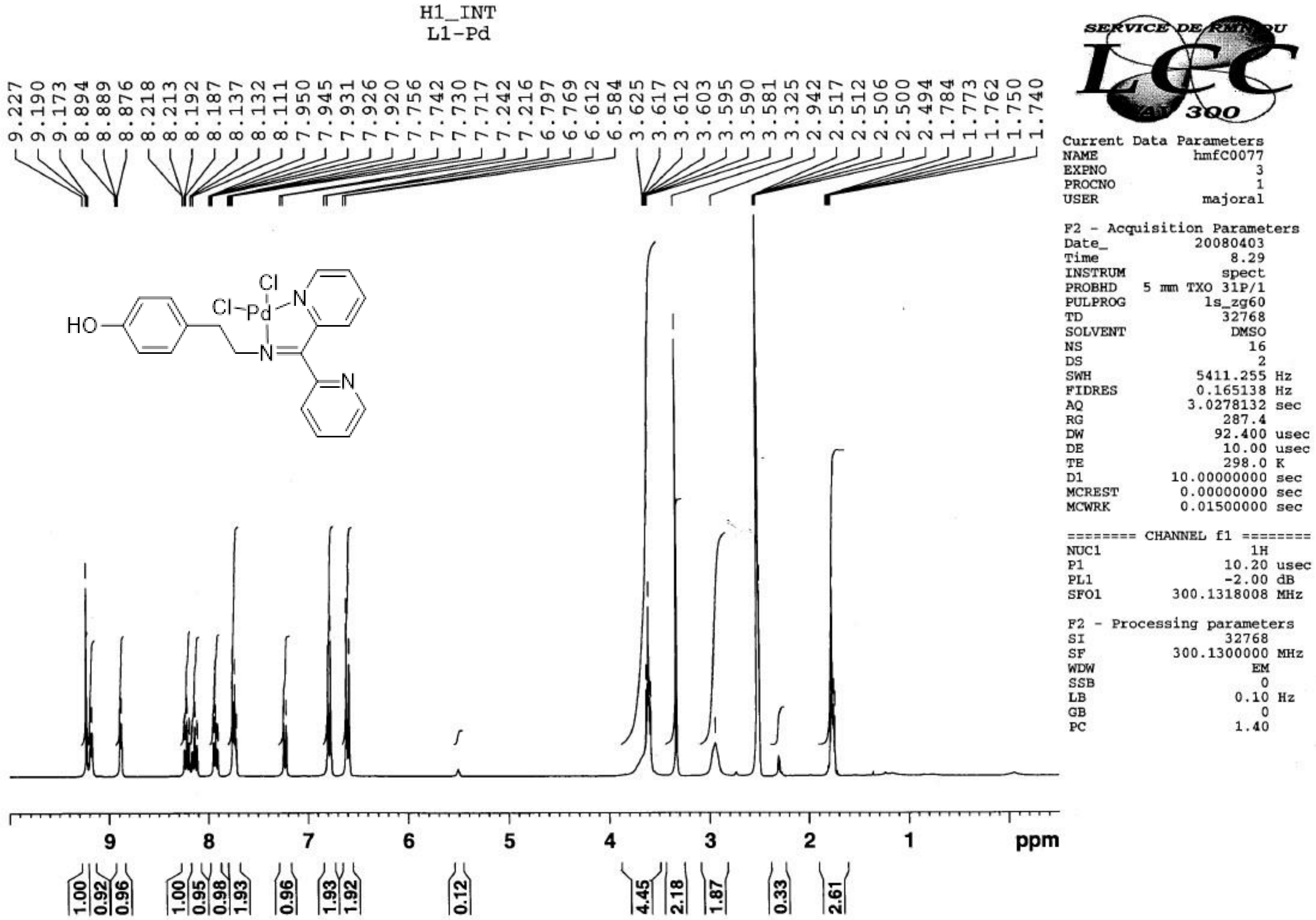

$^{13}C\{^{1}H\}$ NMR of Compound 3-Pd

^{1}H NMR of Compound 3

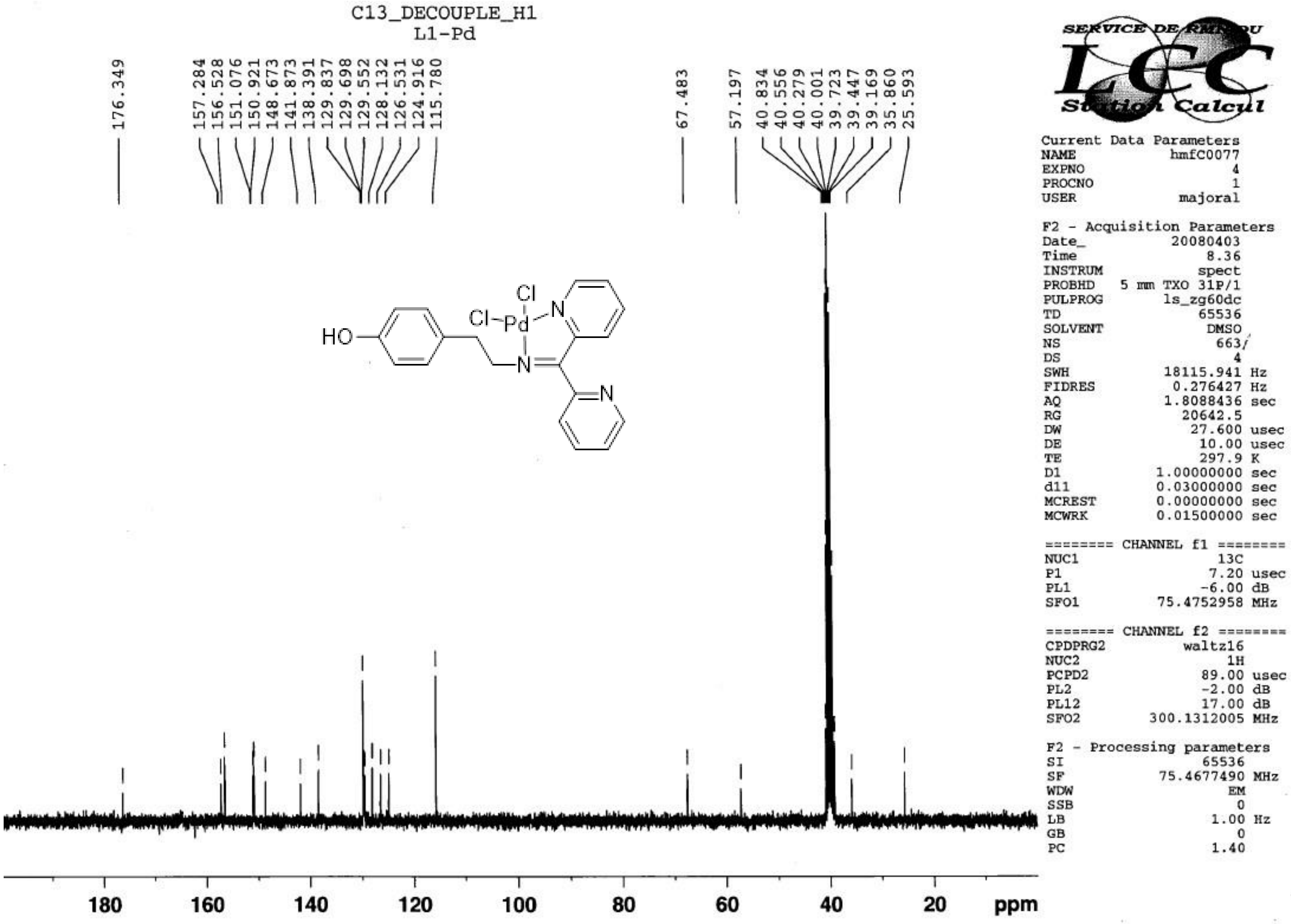

$^{13}C\{^{1}H\}$ NMR of Compound 3

a-Pd

^{1}H NMR of Compound 3

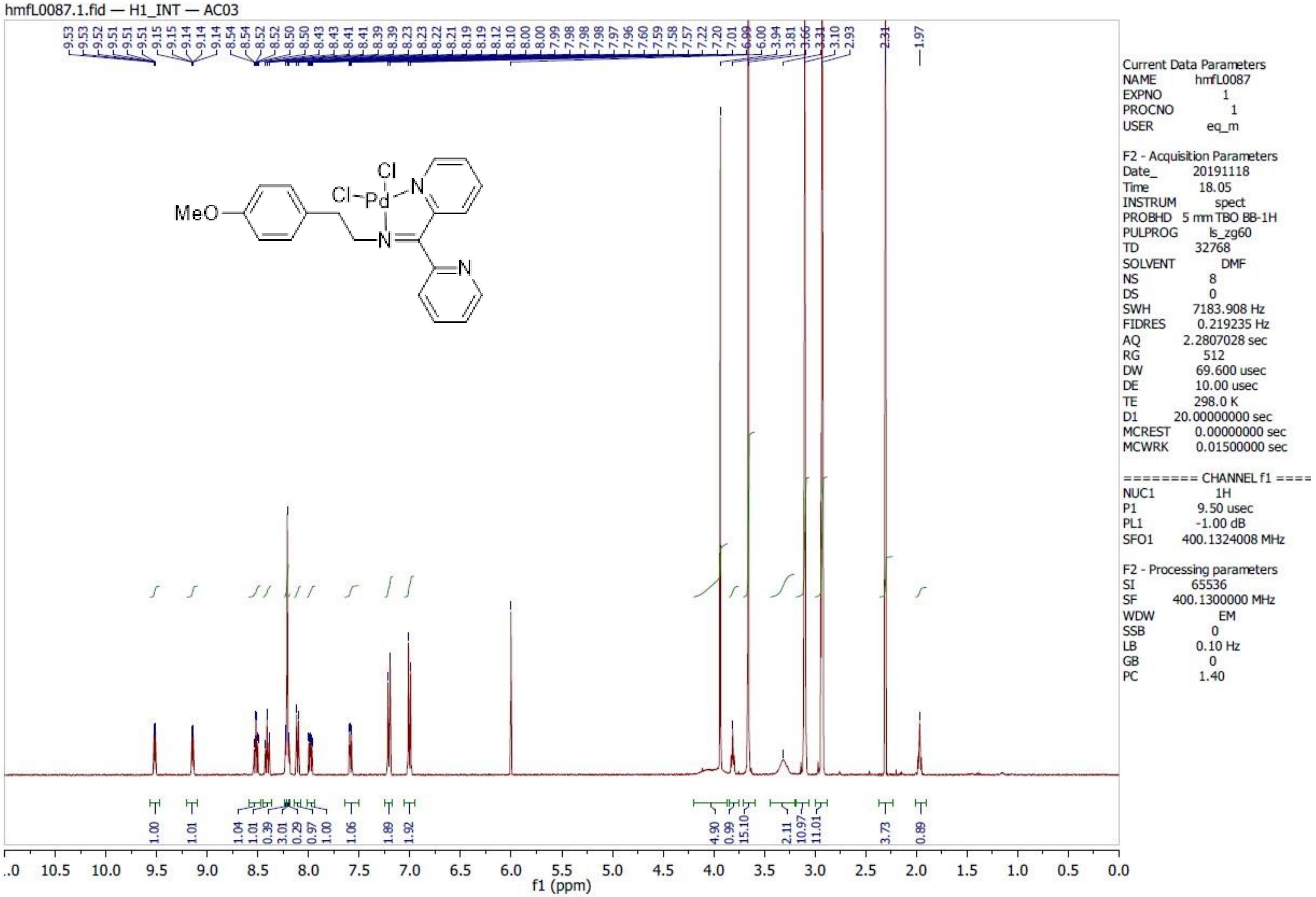

$^{13}C\{^1H\}$ NMR of Compound 3

a-Pd

^{1}H NMR of Compound 3

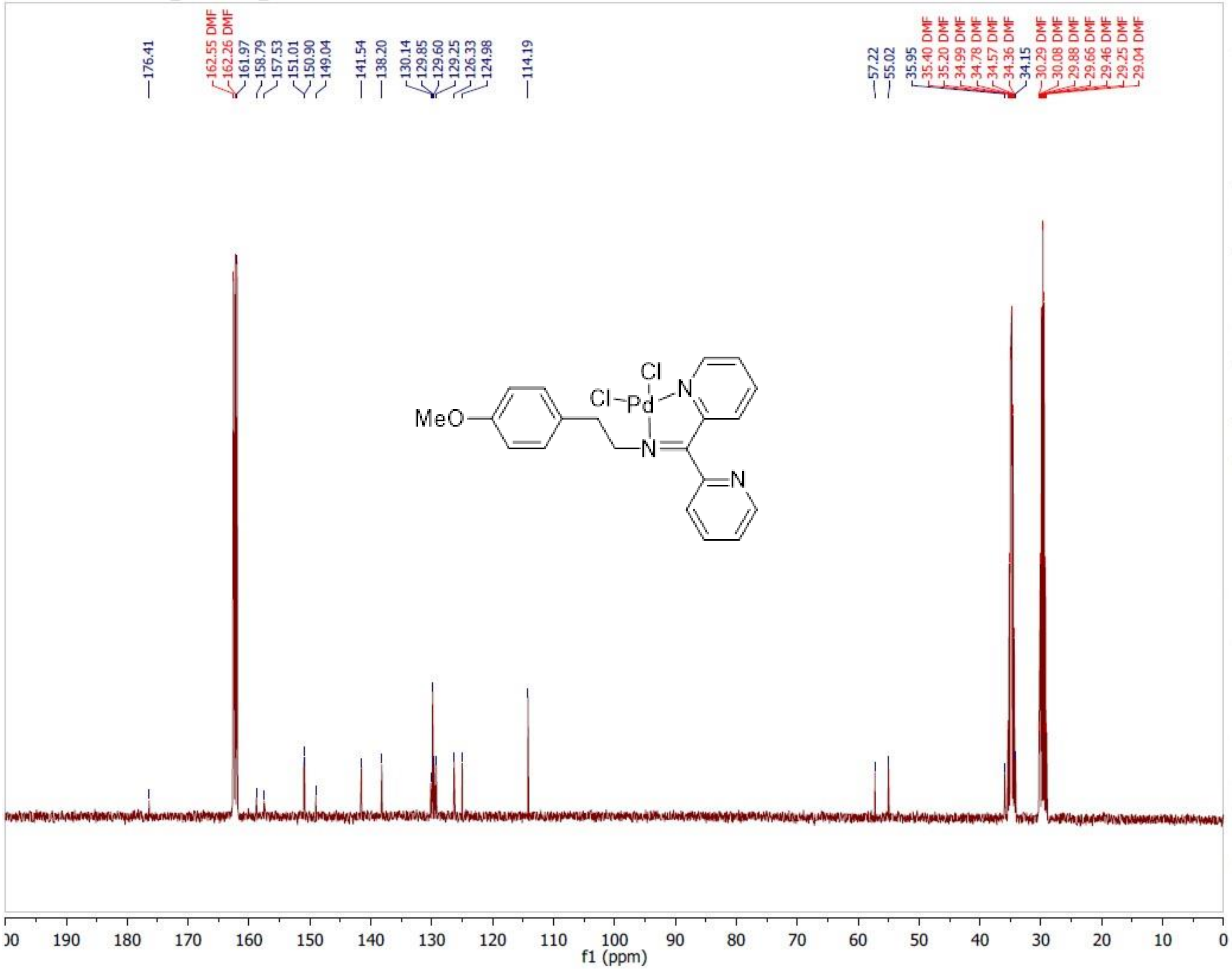

Current Data Parameters
NAME hmfL0087
EXPNO 2
PROCNO 1
USER eq_m

F2 - Acquisition Parameters
Date_ 20191118
Time 21.09
INSTRUM spect
PROBHD 5 mm TBO BB-1H
PULPROG ls_zg60dc
TD 32768
SOLVENT DMF
NS 6400
DS 4
SWH 25125.629 Hz
FIDRES 0.766773 Hz
AQ 0.6521332 sec
RG 26008
DW 19.900 usec
DE 10.00 usec
TE 297.9 K
D1 1.00000000 sec
d11 0.03000000 sec
MCREST 0.00000000 sec
MCWRK 0.01500000 sec

======== CHANNEL f1 ====
NUC1 13C
P1 10.00 usec
PL1 2.00 dB
SFO1 100.6233333 MHz

======== CHANNEL f2 ====
CPDPRG2 waltz16
NUC2 1H
PCPD2 92.00 usec
PL2 -1.00 dB
PL12 18.45 dB
SFO2 400.1318006 MHz

F2 - Processing parameters
SI 65536
SF 100.6127690 MHz
WDW EM
SSB 0
LB 1.00 Hz
GB 0
PC 0.30

^{1}H NMR of Compound 4

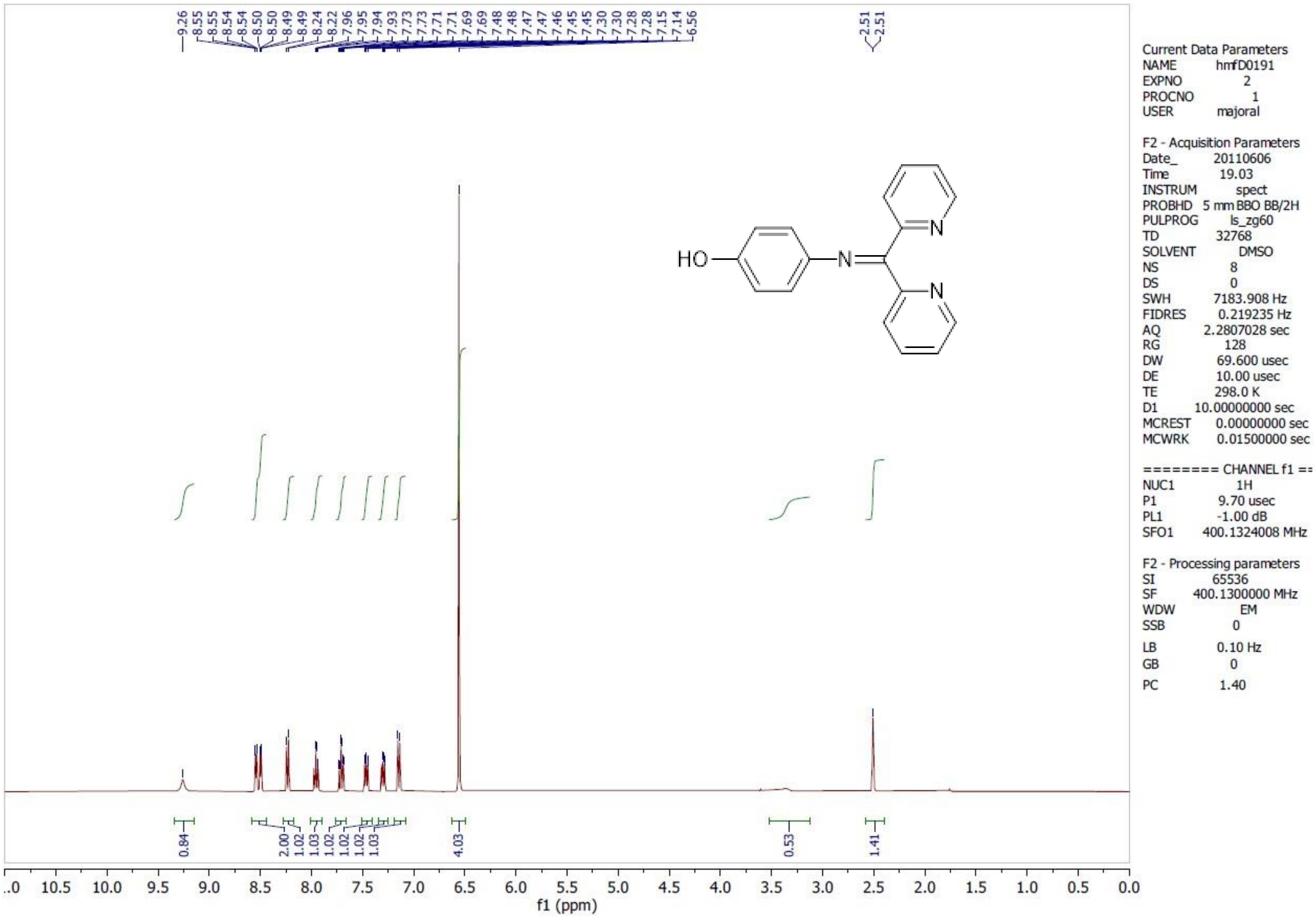

¹H COSY NMR of Compound 4

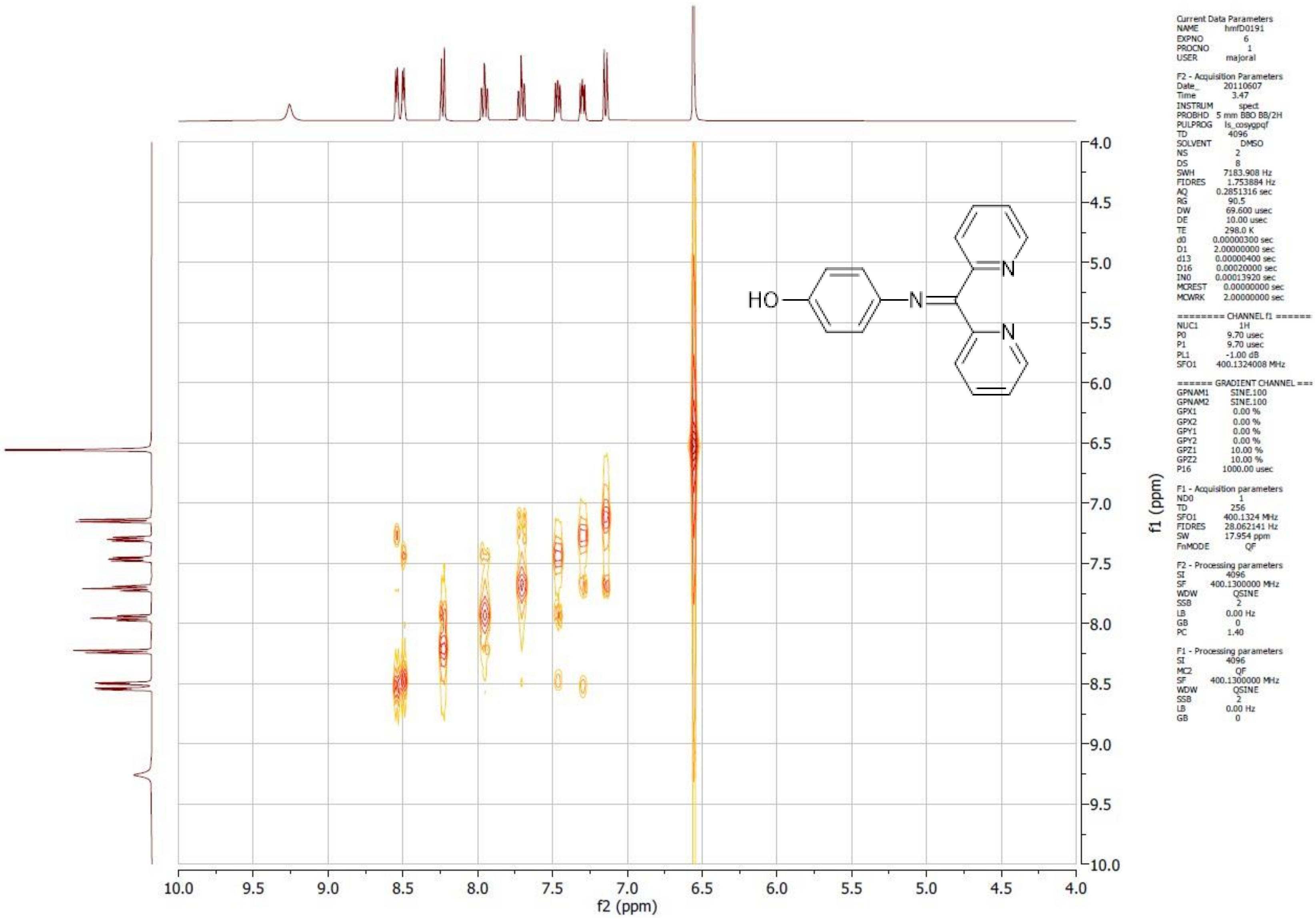

$^{13}C\{^{1}H\}$ NMR of Compound 4

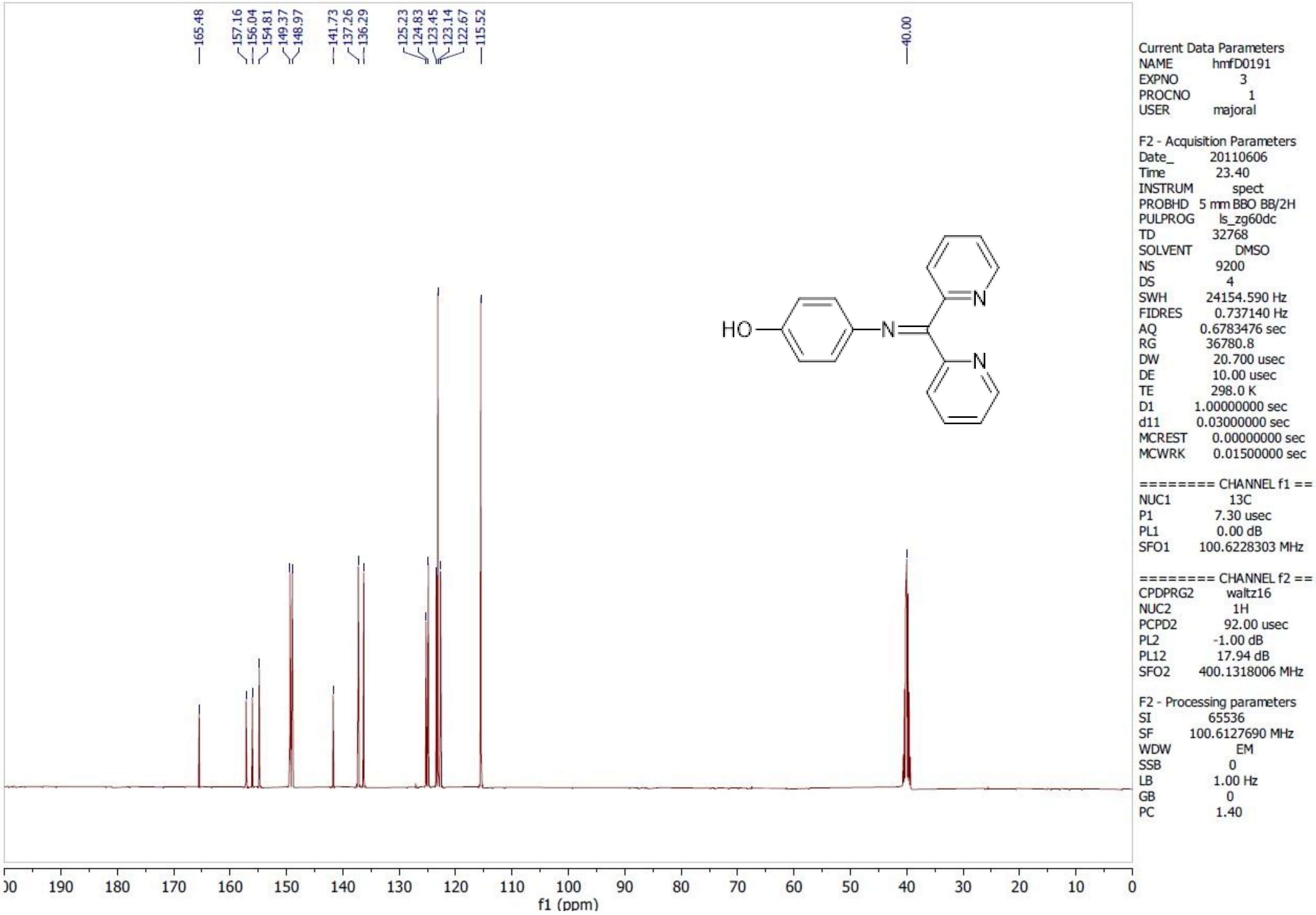

^{13}C HMQC NMR of Compound 4

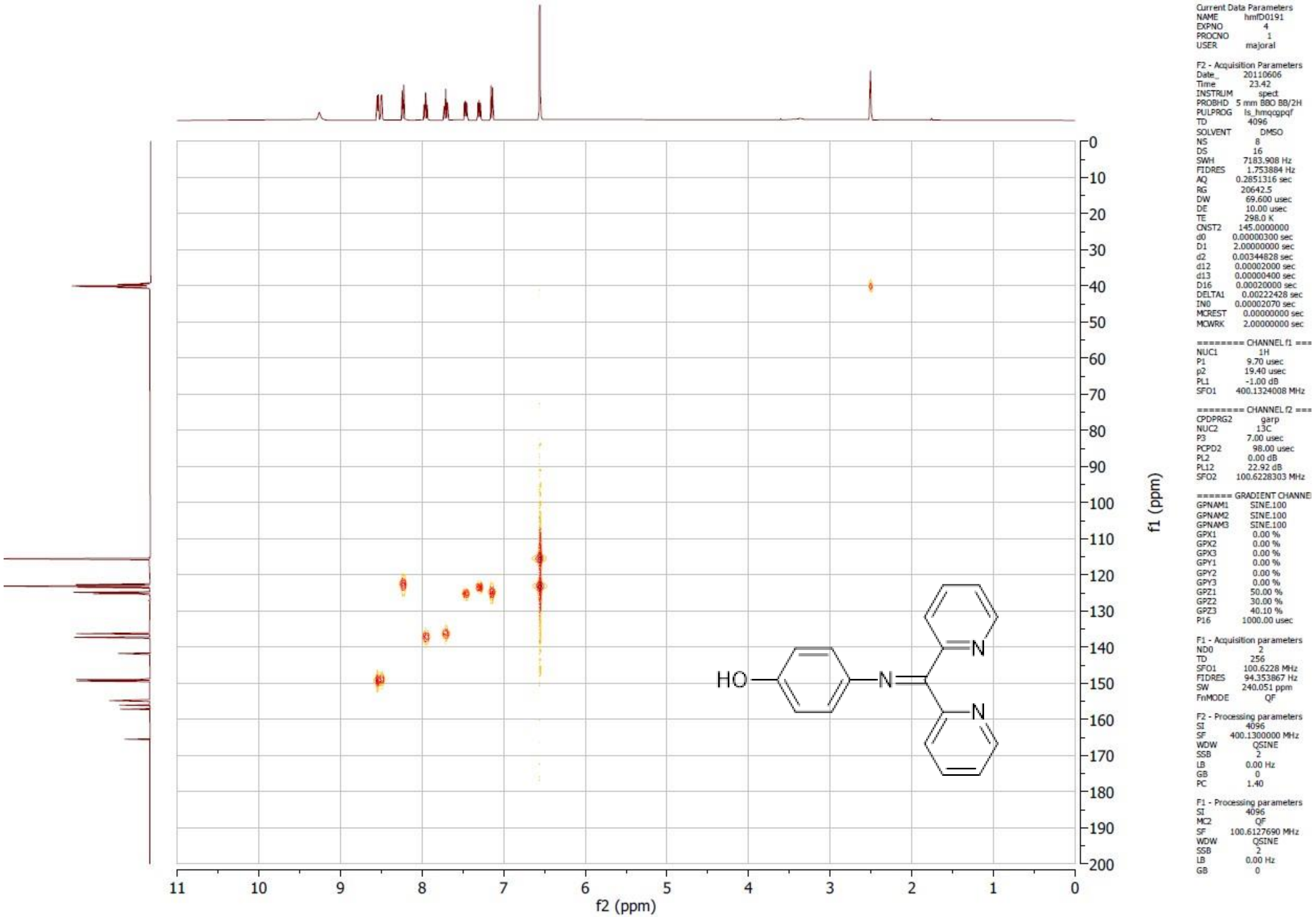

^{13}C HMQC long range NMR of Compound 4

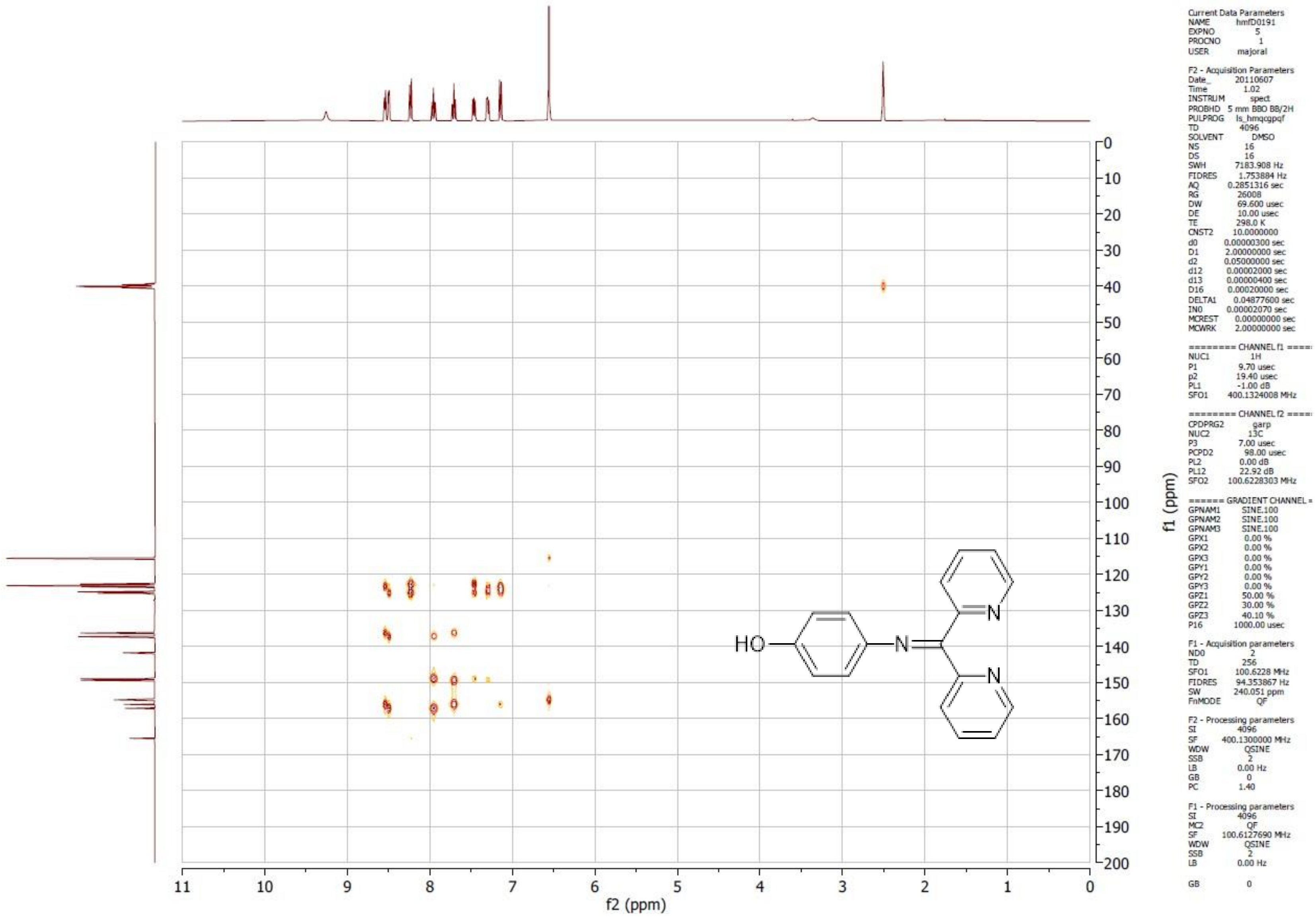

¹H NMR of Compound 5

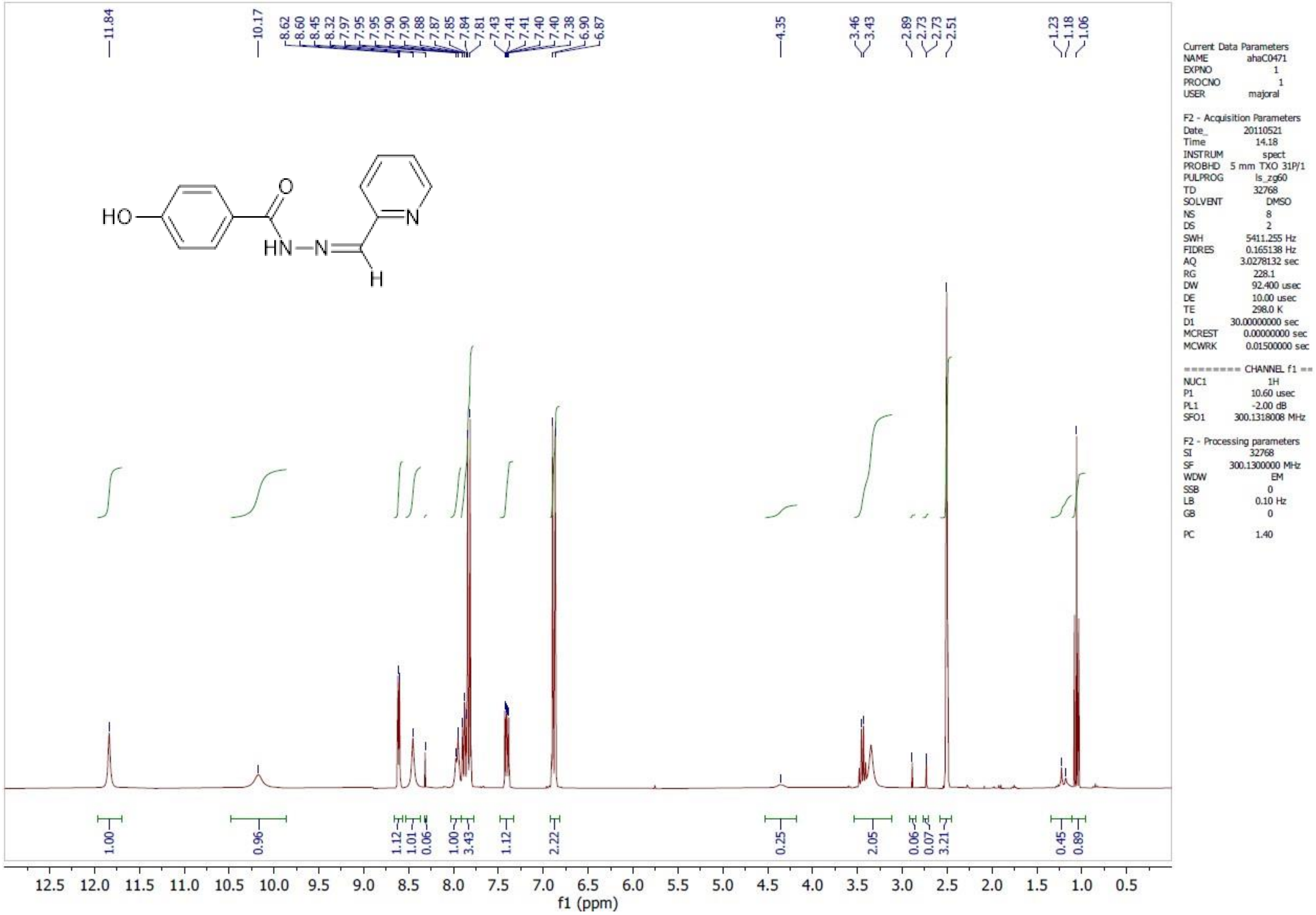

^{1}H COSY NMR of Compound 5

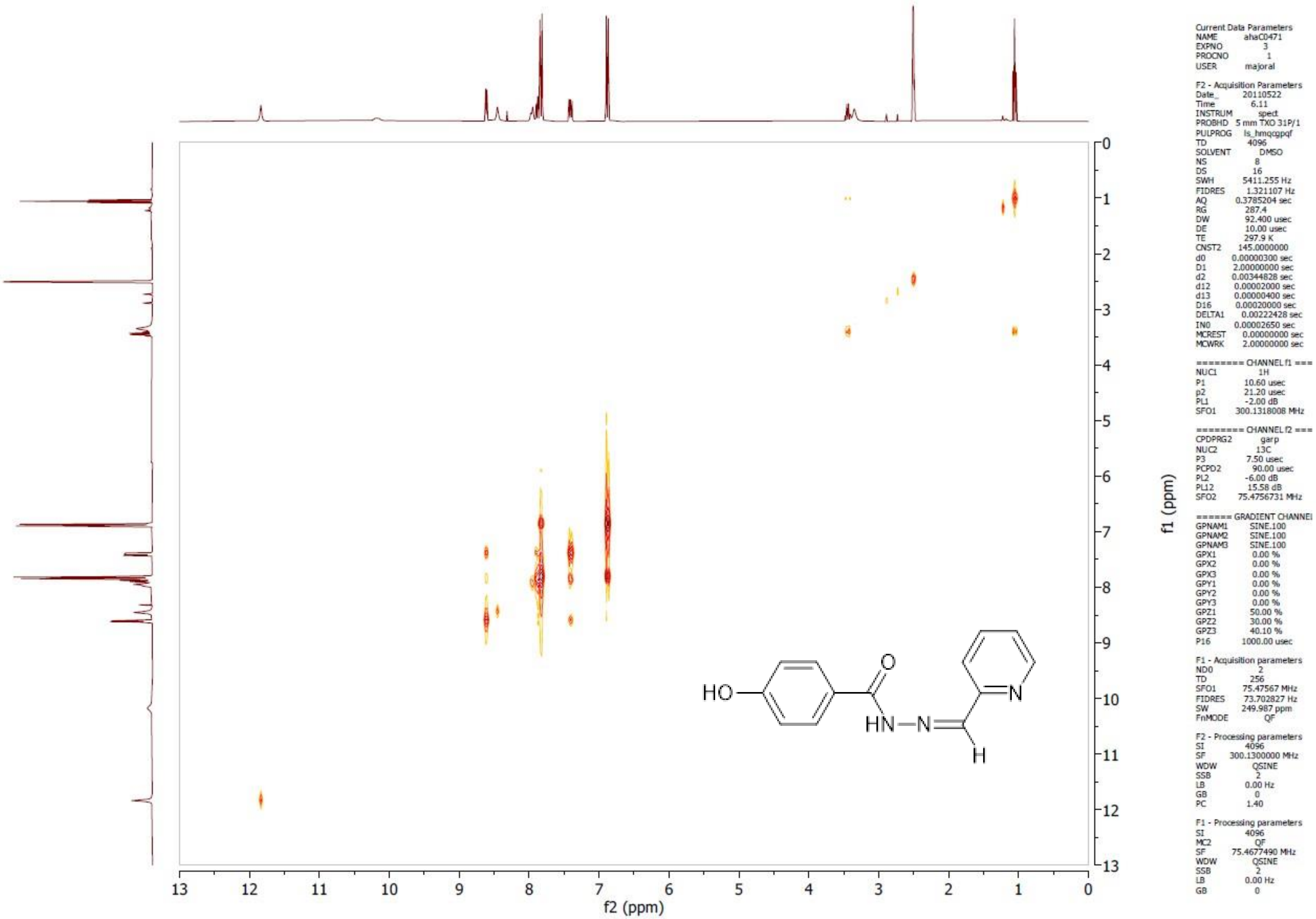

$^{13}C\{^1H\}$ **NMR of Compound 5**

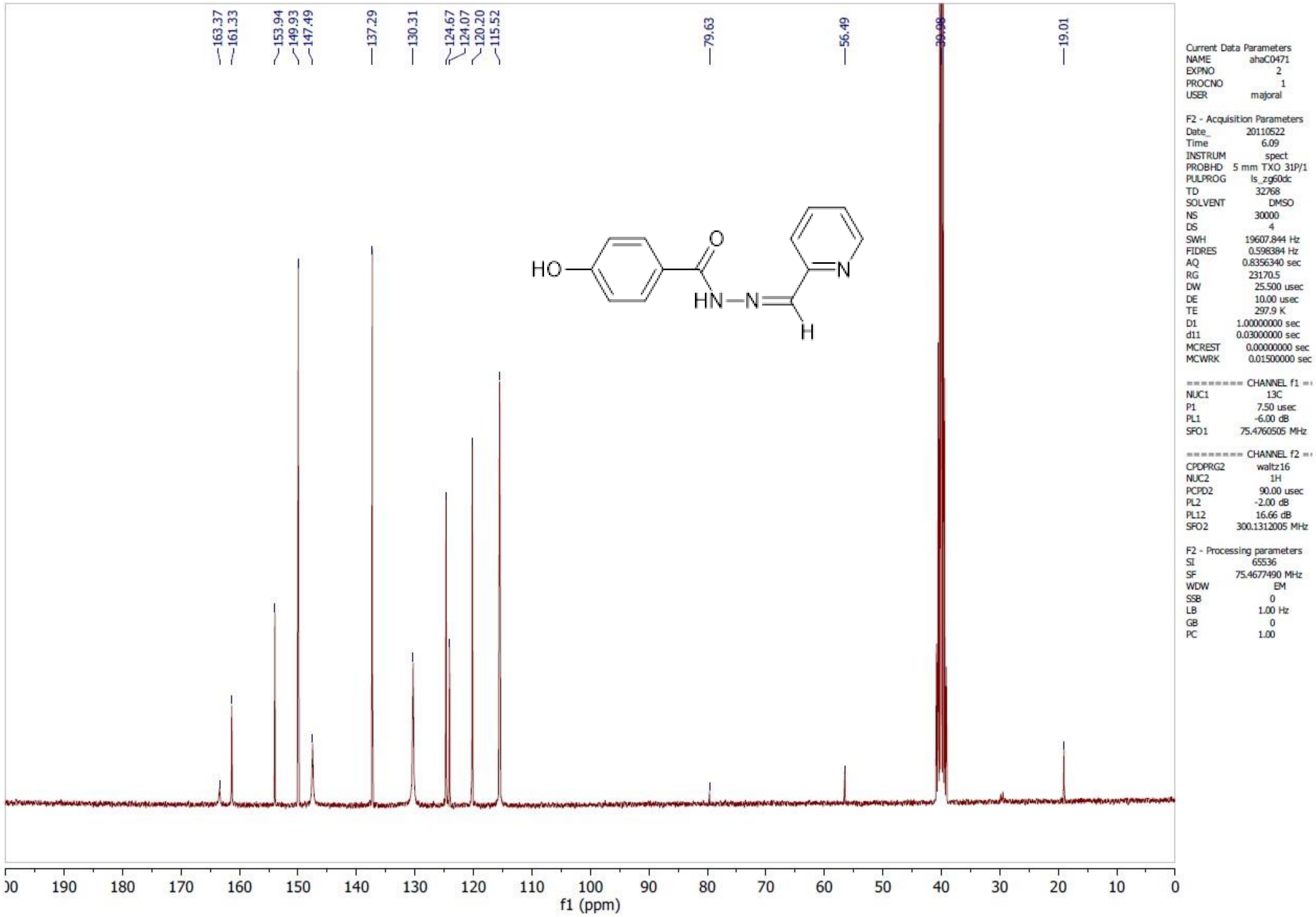

^{13}C HMBC NMR of Compound 5

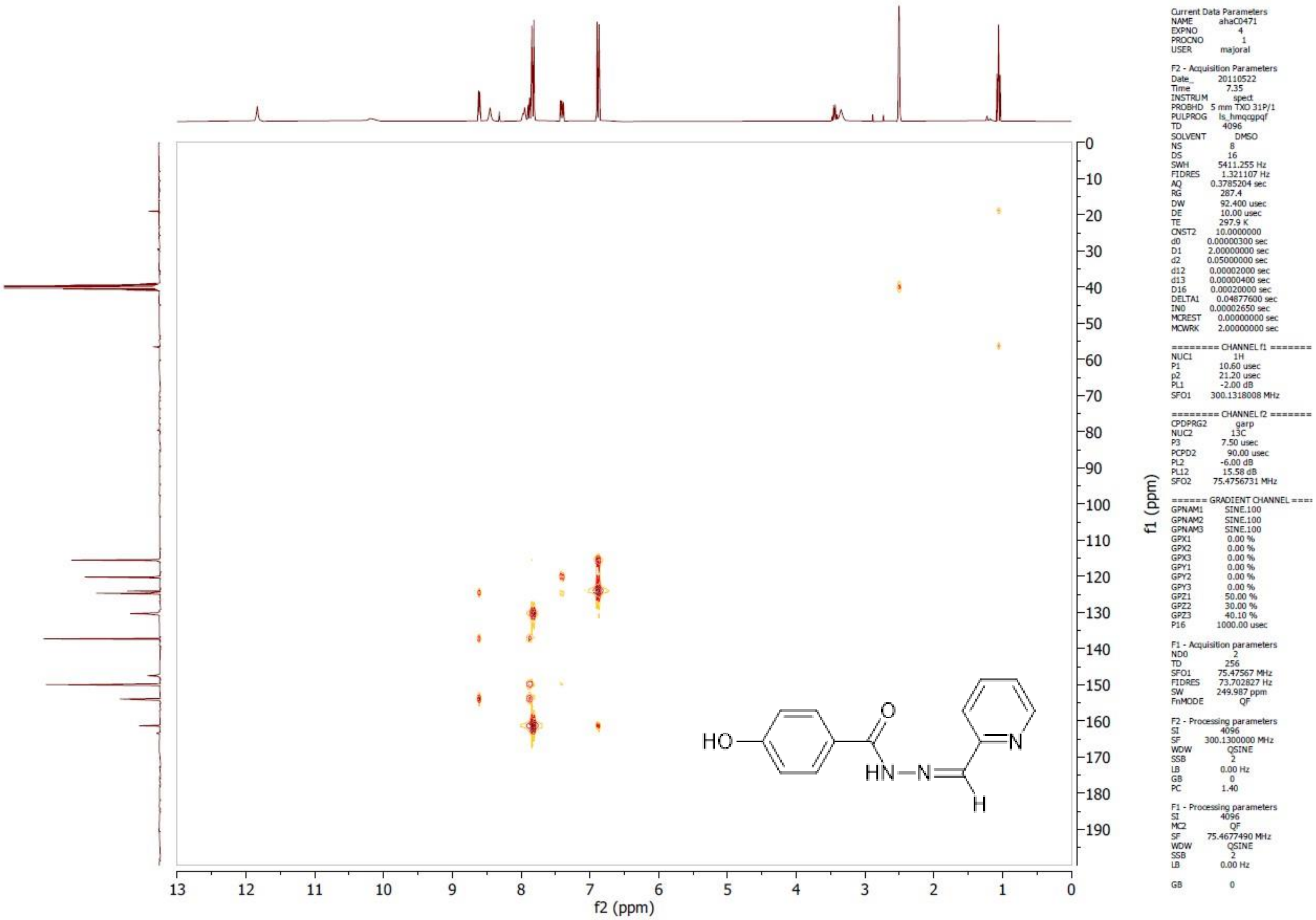

^{13}C HMQC NMR of Compound 5

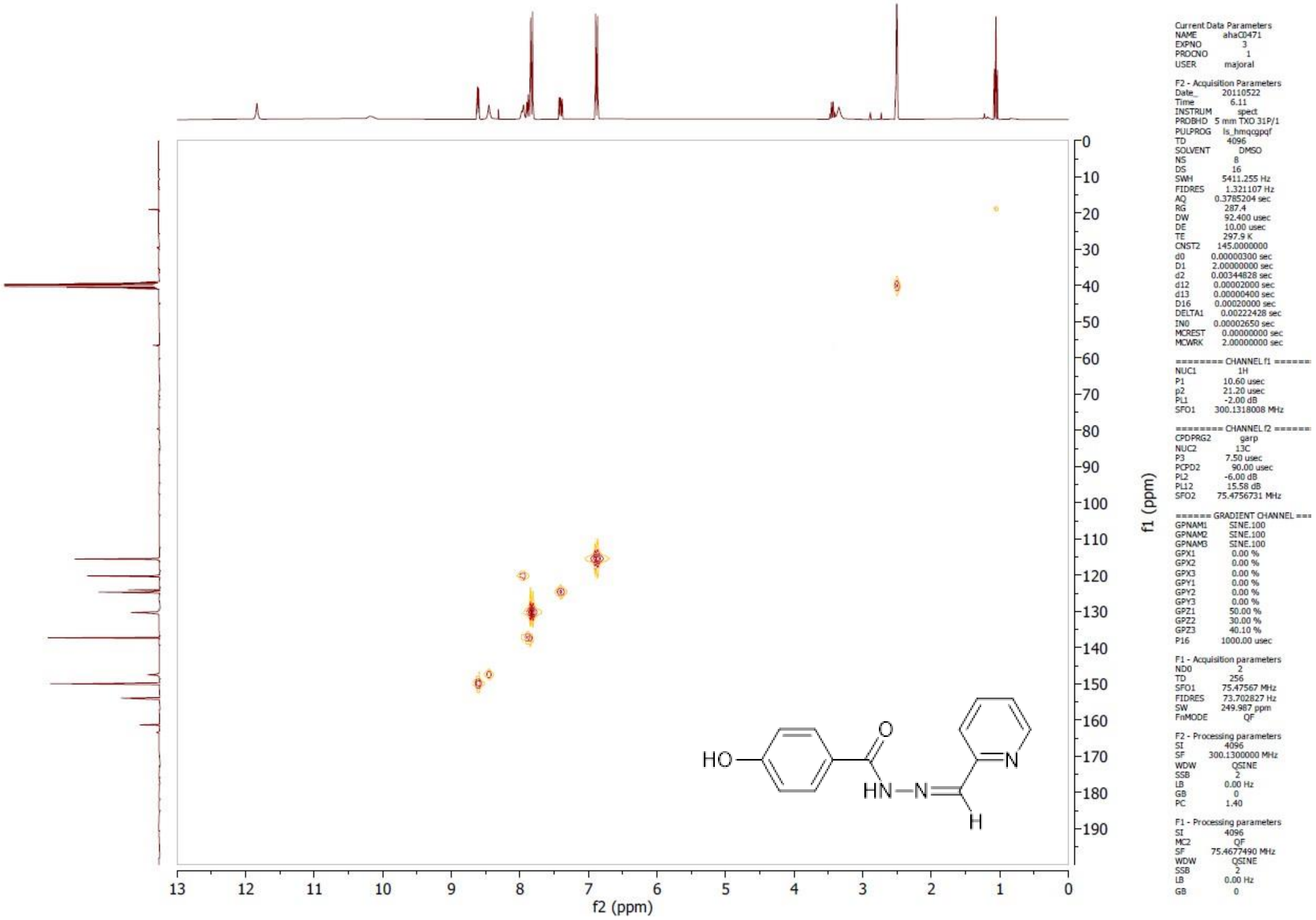

^{1}H NMR of Compound 6

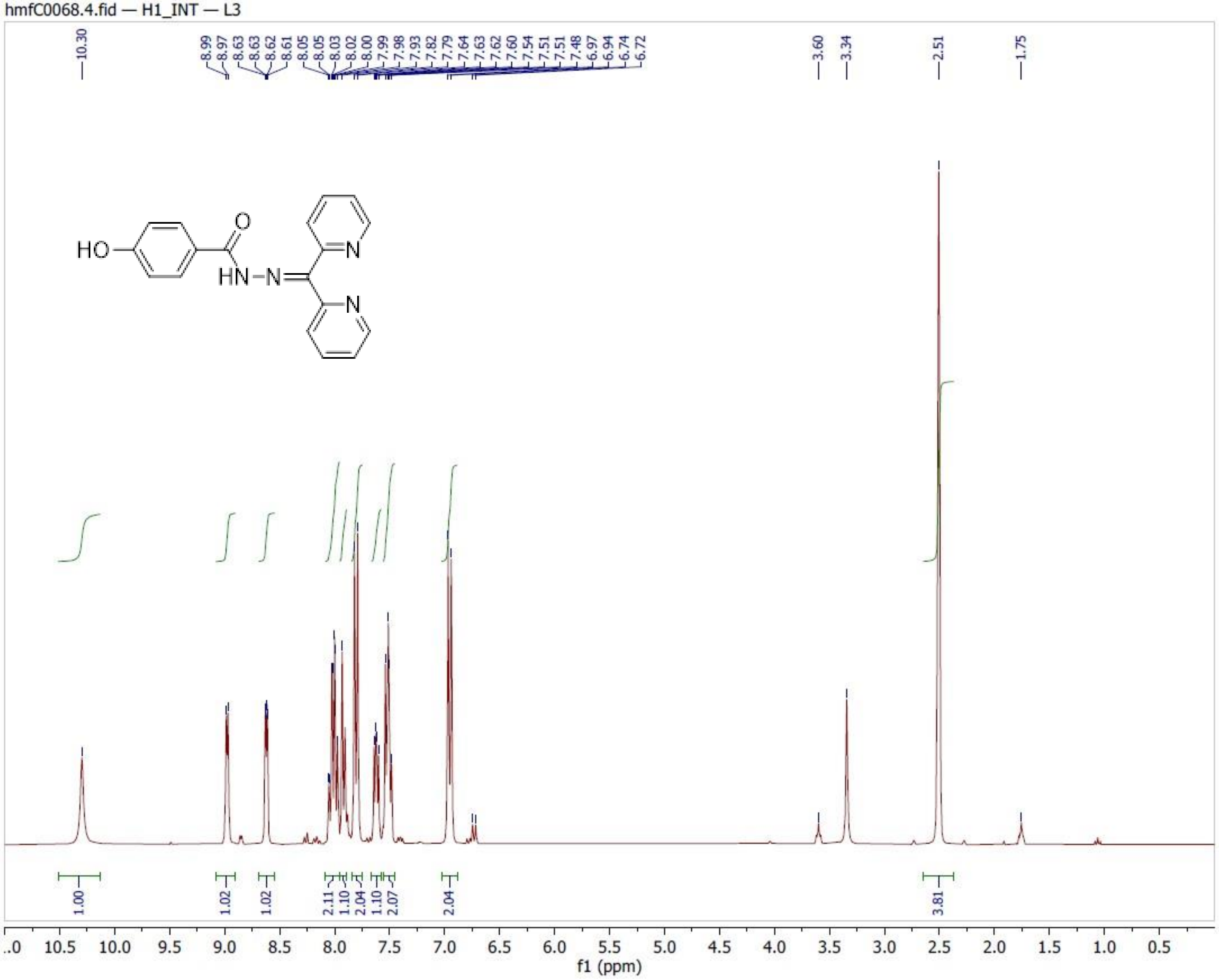

Current Data Parameters	
NAME	hmfC0068
EXPNO	4
PROCNO	1
USER	majoral
F2 - Acquisition Parameters	
Date_	20080327
Time	8.52
INSTRUM	spect
PROBHD	5 mm TXO 31P/1
PULPROG	ls_zg60
TD	32768
SOLVENT	DMSO
NS	8
DS	2
SWH	5411.255 Hz
FIDRES	0.165138 Hz
AQ	3.0278132 sec
RG	228.1
DW	92.400 usec
DE	10.00 usec
TE	298.0 K
D1	10.00000000 sec
MCREST	0.00000000 sec
MCWRK	0.01500000 sec
======== CHANNEL f1 ==	
NUC1	1H
P1	10.20 usec
PL1	-2.00 dB
SFO1	300.1318008 MHz
F2 - Processing parameters	
SI	32768
SF	300.1300000 MHz
WDW	EM
SSB	0
LB	0.10 Hz
GB	0
PC	1.40

$^{13}C\{^{1}H\}$ NMR of Compound 6

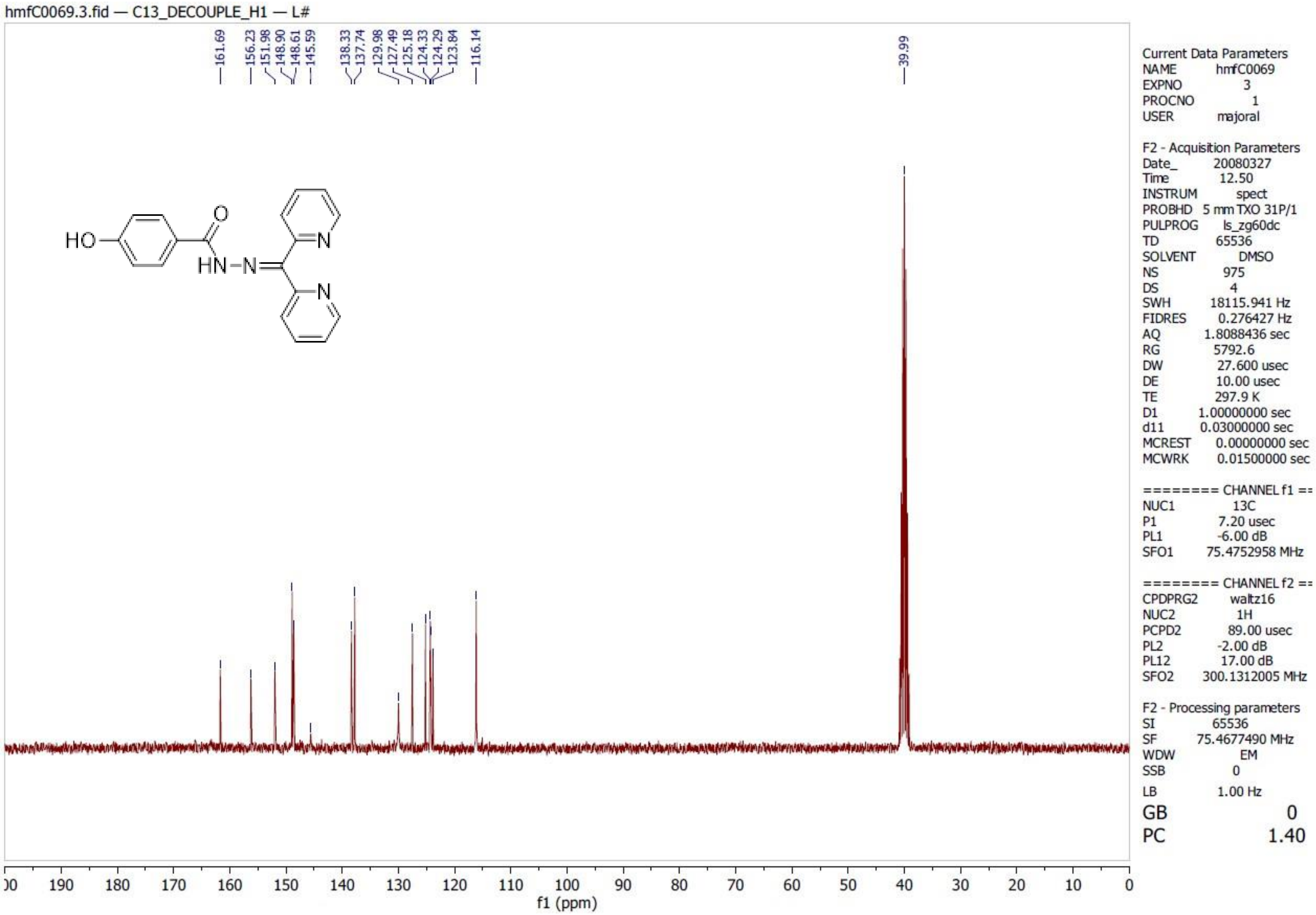

[13]C HMQC of Compound 6

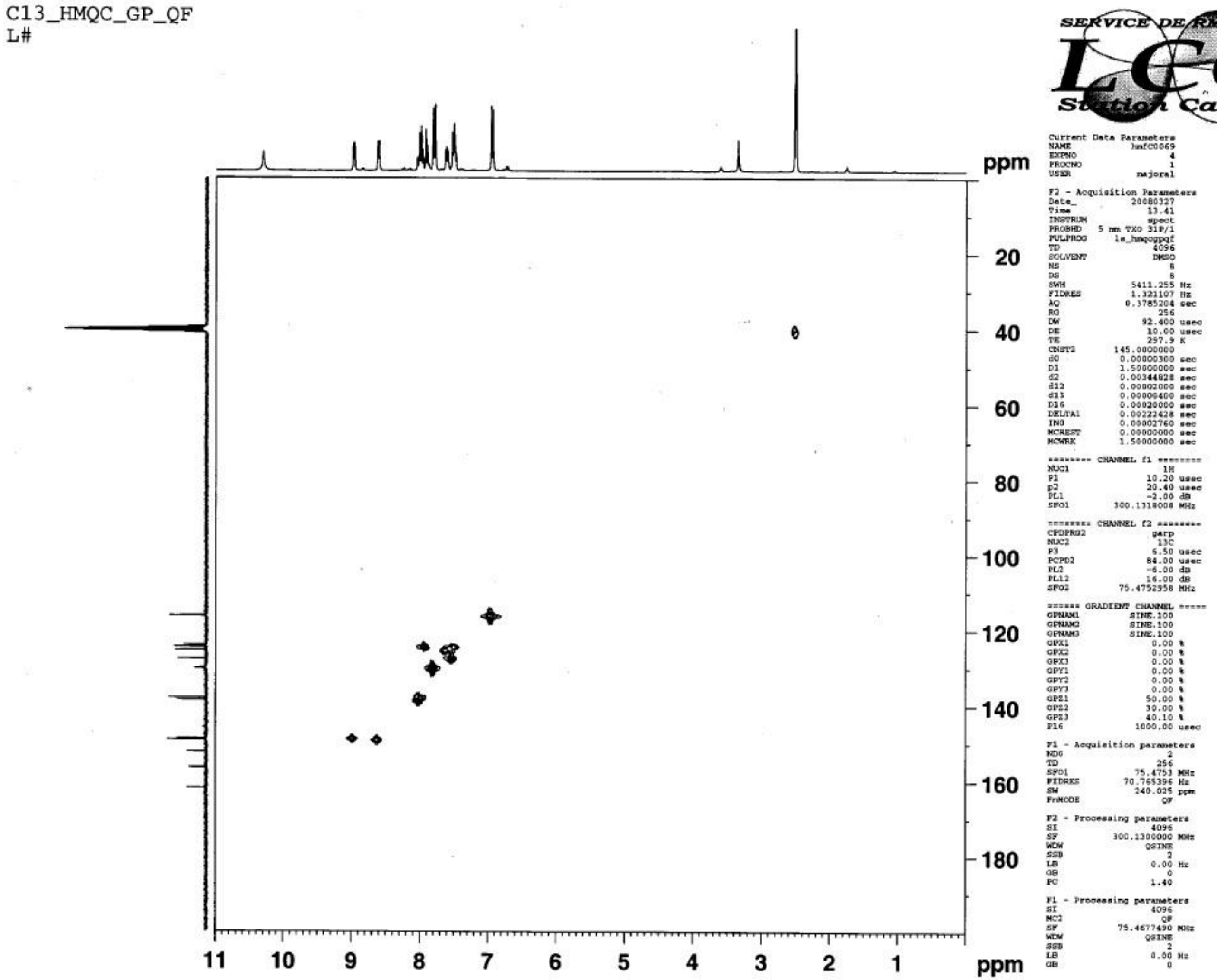

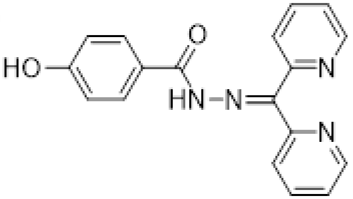

$^{31}P\{^1H\}$ NMR during the course of the synthesis of 1-G$_0$

$^{31}P\{^1$

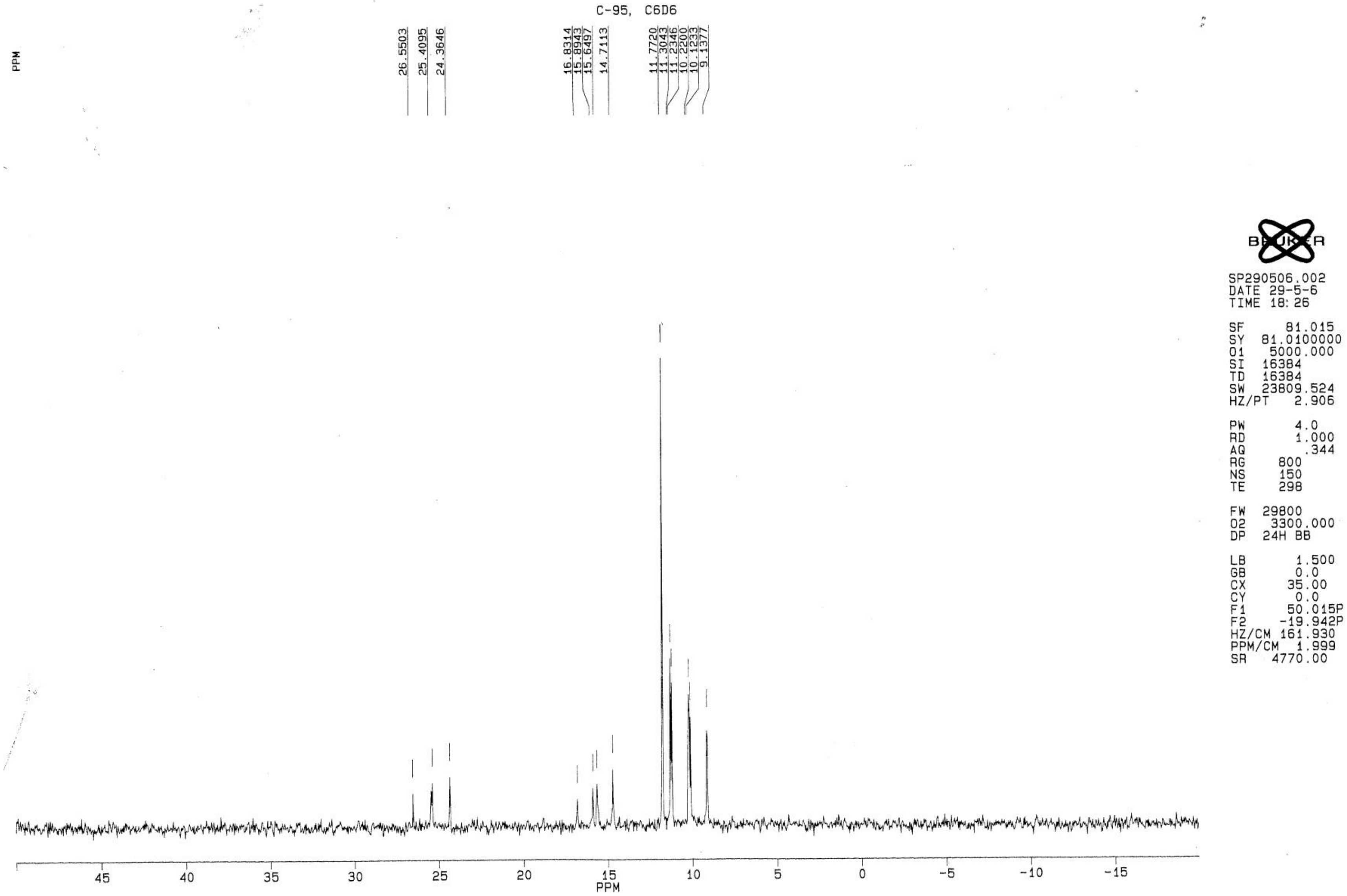

C-95, C6D6
PPM
26.5503
25.4095
24.3646
16.8314
15.8943
15.6497
14.7113
11.7720
11.3043
11.2346
10.2200
10.1233
9.1377
SP290506.002
DATE 29-5-6
TIME 18:26
SF 81.015
SY 81.0100000
O1 5000.000
SI 16384
TD 16384
SW 23809.524
HZ/PT 2.906
PW 4.0
RD 1.000
AQ .344
RG 800
NS 150
TE 298
FW 29800
O2 3300.000
DP 24H BB
LB 1.500
GB 0.0
CX 35.00
CY 0.0
F1 50.015P
F2 -19.942P
HZ/CM 161.930
PPM/CM 1.999
SR 4770.00
45 40 35 30 25 20 15 10 5 0 -5 -10 -15
PPM

$^{31}P\{^1H\}$ NMR of 1-G_0

^{31}P {1

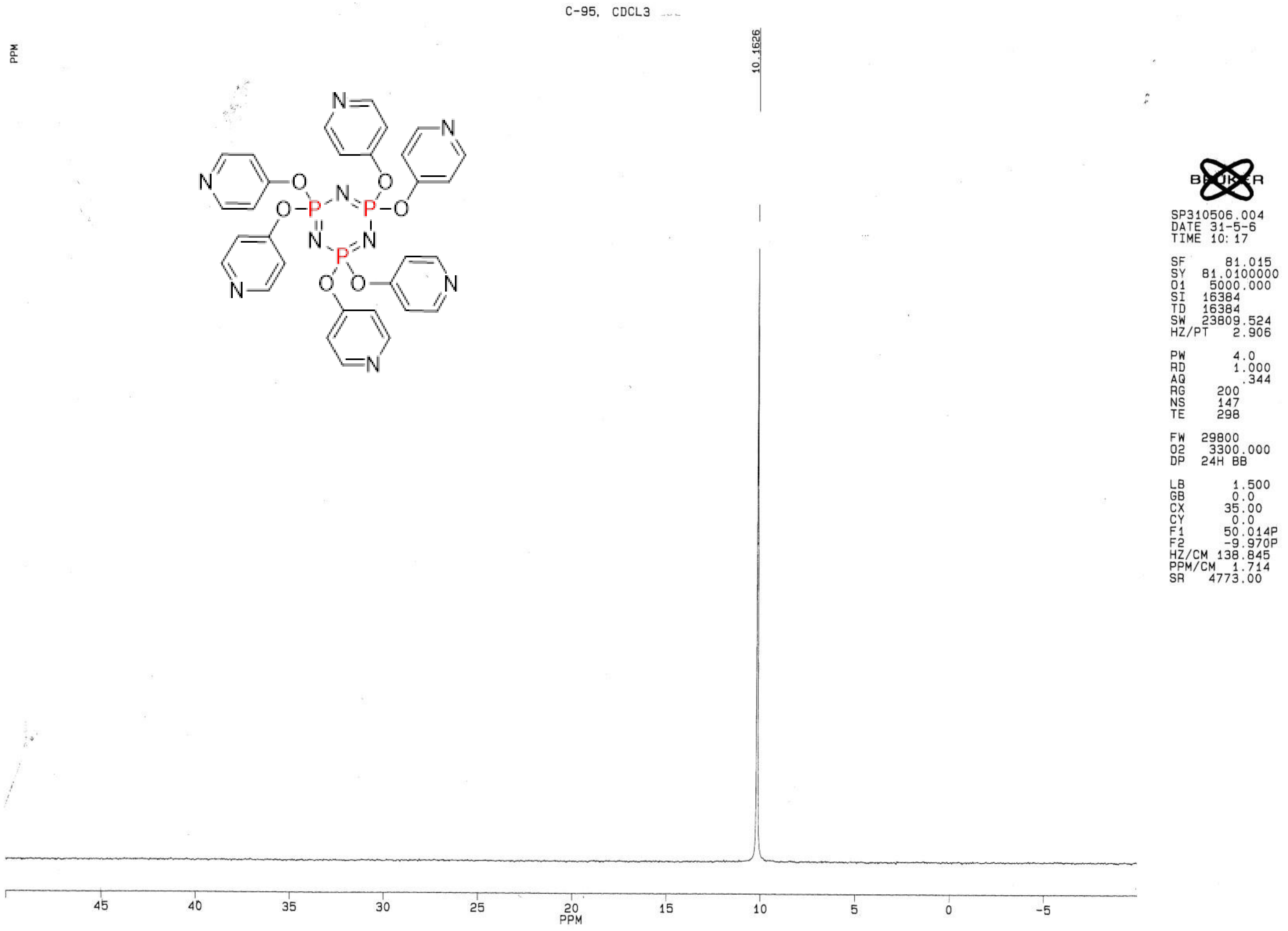

C-95, CDCL3
10.1626
PPM
SP310506.004
DATE 31-5-6
TIME 10:17
SF 81.015
SY 81.0100000
O1 5000.000
SI 16384
TD 16384
SW 23809.524
HZ/PT 2.906
PW 4.0
RD 1.000
AQ .344
RG 200
NS 147
TE 298
FW 29800
O2 3300.000
DP 24H BB
LB 1.500
GB 0.0
CX 35.00
CY 0.0
F1 50.014P
F2 -9.970P
HZ/CM 138.845
PPM/CM 1.714
SR 4773.00
45 40 35 30 25 20 15 10 5 0 -5
PPM

$^{31}P\{^{1}H\}$ NMR of dendrimer 1-G$_1$

^{31}P {1

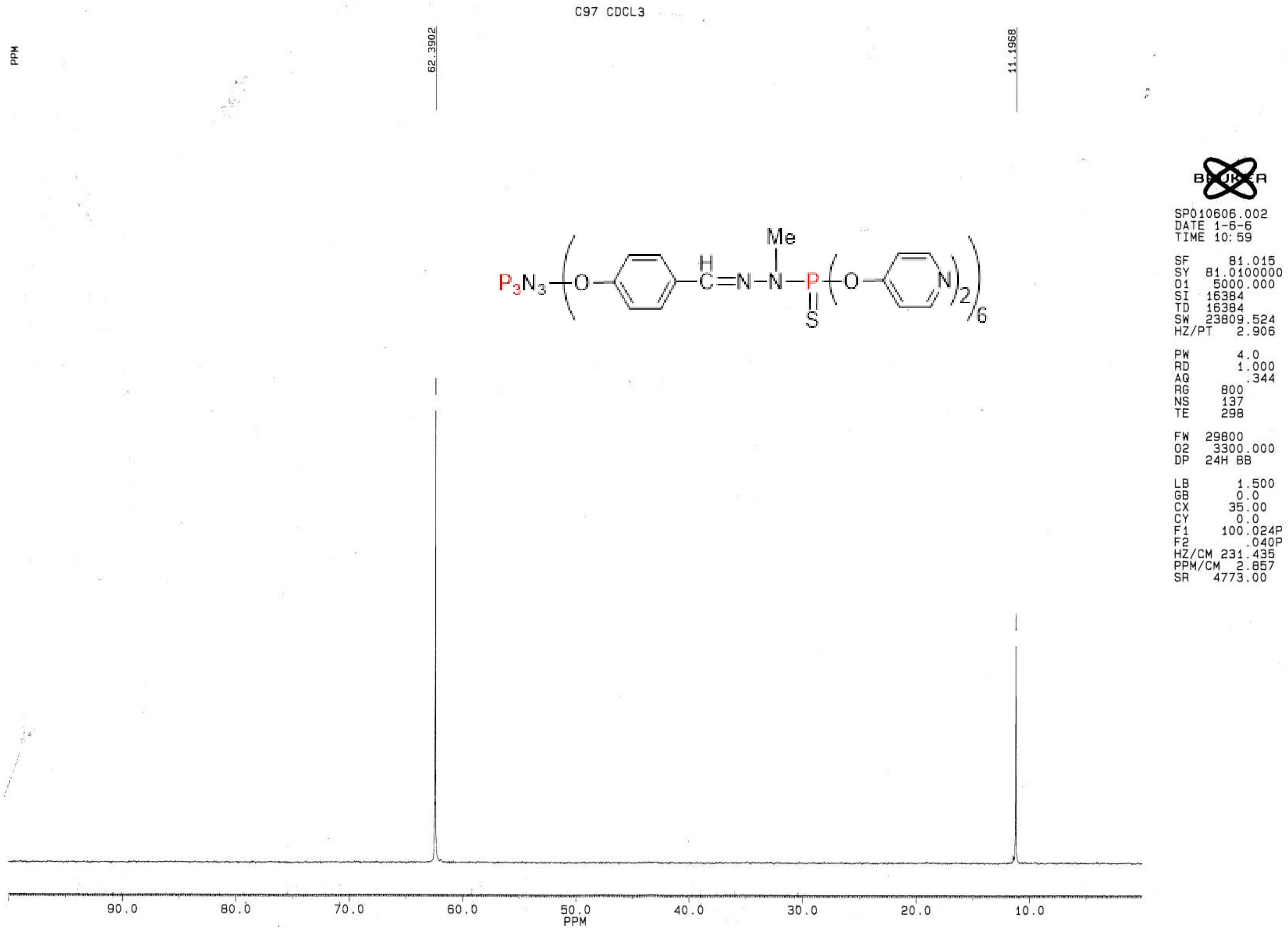
C97 CDCL3
PPM
62.3902
11.1968
P3N3
O
H
C=N
N
Me
P
S
O
N
2
6
SP010606.002
DATE 1-6-6
TIME 10:59
SF 81.015
SY 81.0100000
O1 5000.000
SI 16384
TD 16384
SW 23809.524
HZ/PT 2.906
PW 4.0
RD 1.000
AQ .344
RG 800
NS 137
TE 298
FW 29800
O2 3300.000
DP 24H BB
LB 1.500
GB 0.0
CX 35.00
CY 0.0
F1 100.024P
F2 .040P
HZ/CM 231.435
PPM/CM 2.857
SR 4773.00
90.0
80.0
70.0
60.0
50.0
40.0
30.0
20.0
10.0
PPM

^{1}H NMR of dendrimer 1-G$_1$

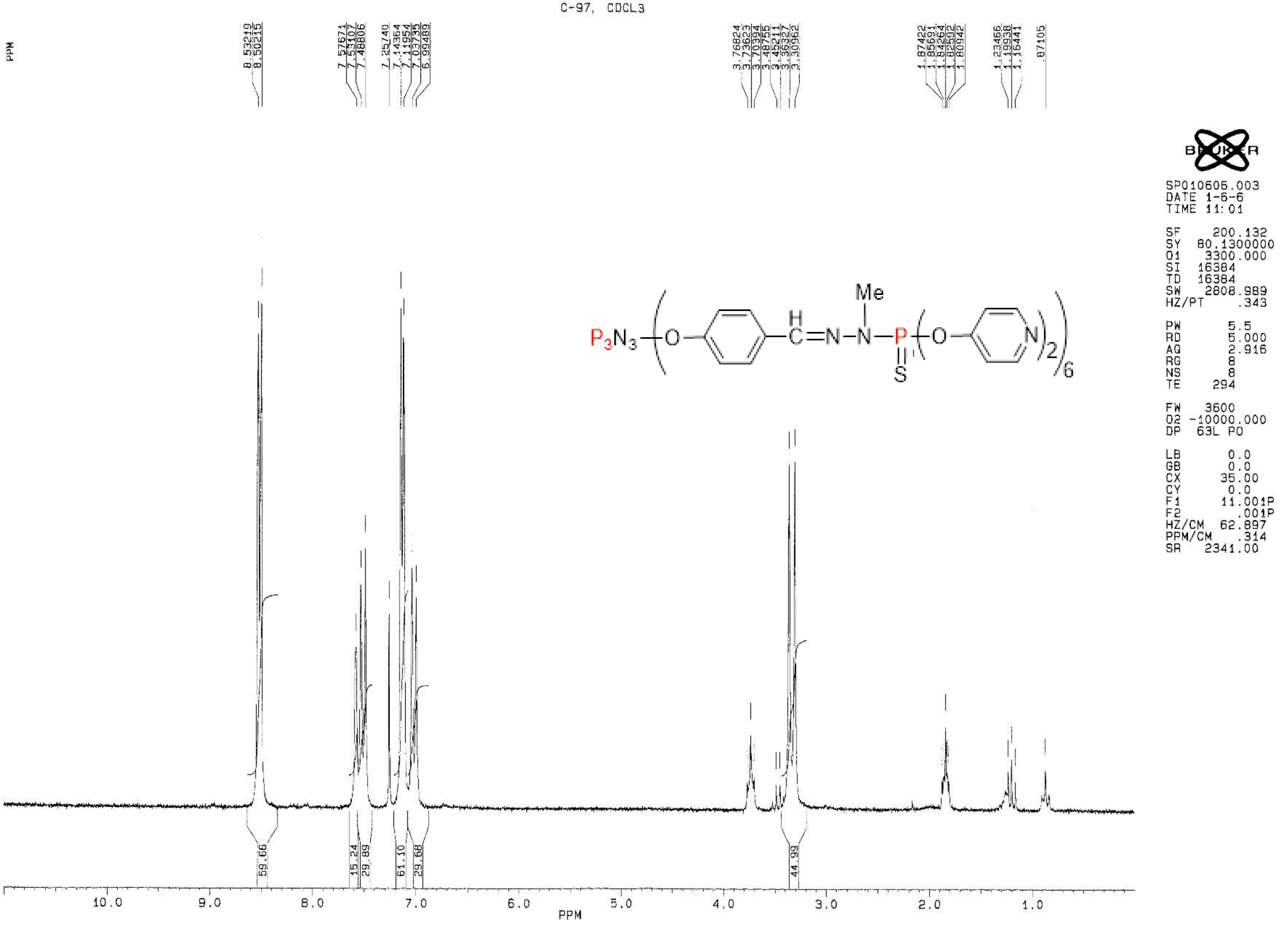

C-97, CDCL3
SP010606.003
DATE 1-6-6
TIME 11:01
SF 200.132
SY 80.1300000
O1 3300.000
SI 16384
TD 16384
SW 2808.989
HZ/PT .343
PW 5.5
RD 5.000
AQ 2.916
RG 8
NS 8
TE 294
FW 3600
O2 -10000.000
DP 63L PO
LB 0.0
GB 0.0
CX 35.00
CY 0.0
F1 11.001P
F2 .001P
HZ/CM 62.897
PPM/CM .314
SR 2341.00
PPM

$^{13}C\{^{1}H\}$ NMR of dendrimer 1-G$_1$

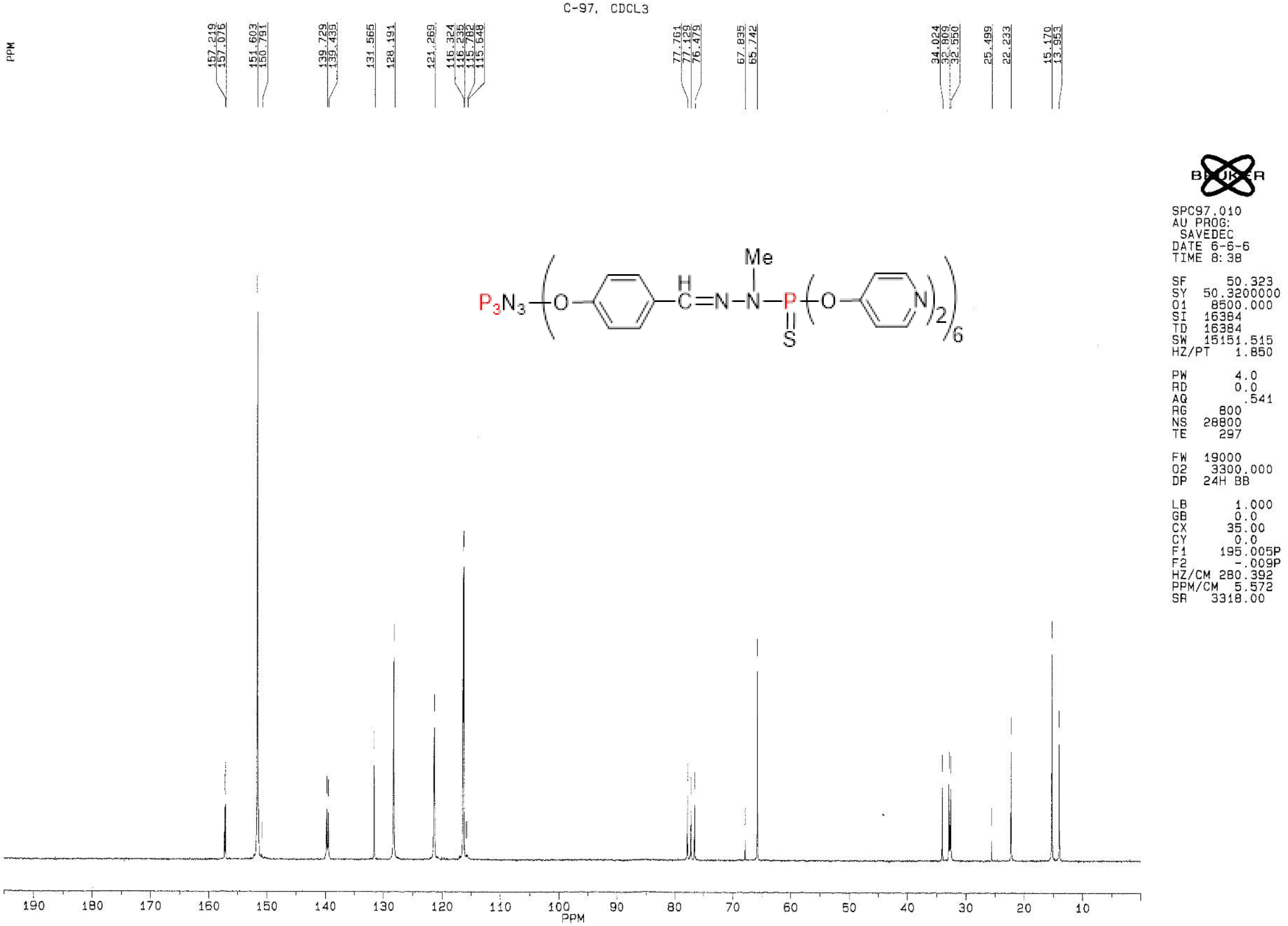

^{31}P {^{1}H} NMR of dendrimer 3-G_1-Pd_{12}

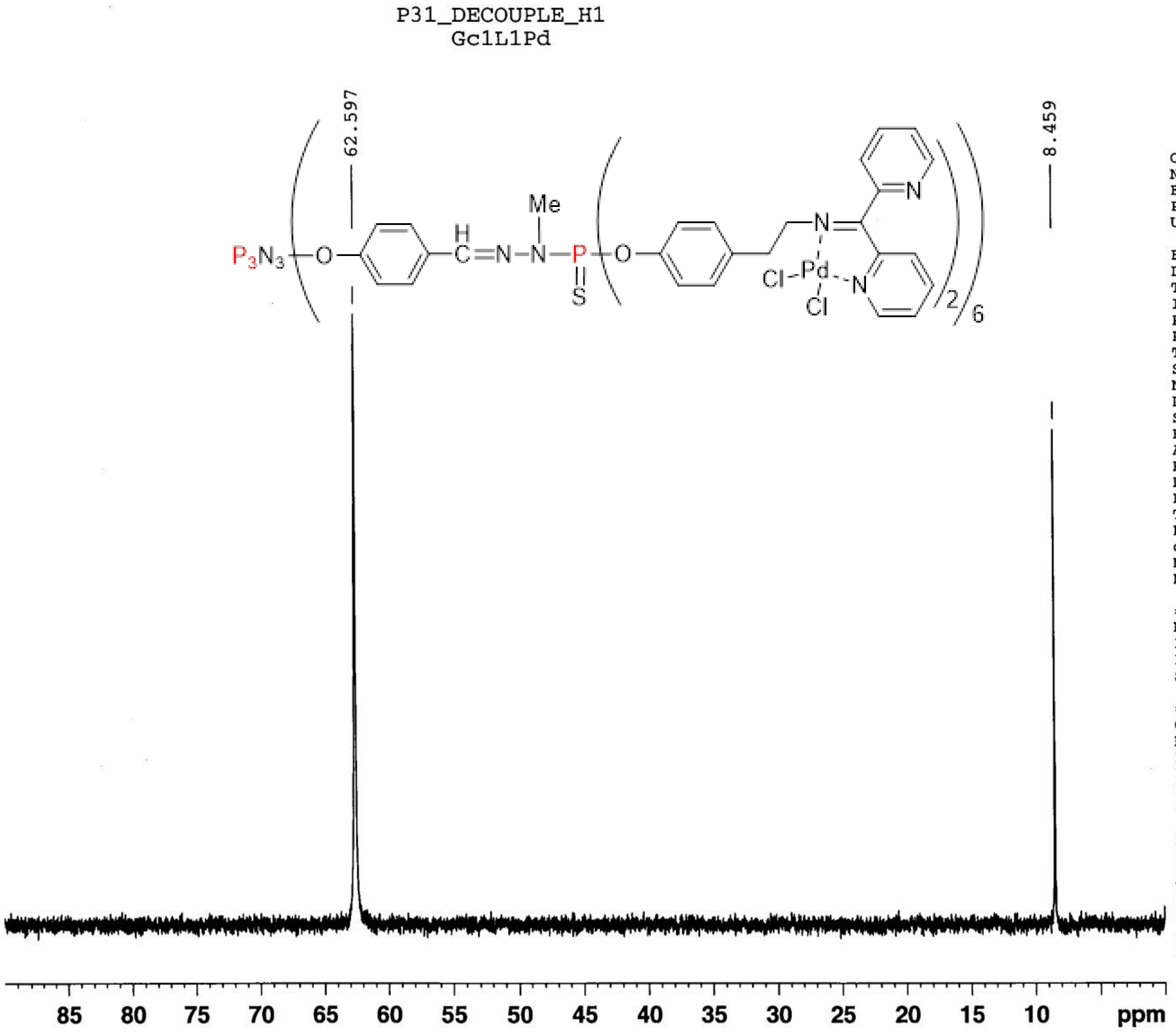


```
Current Data Parameters
NAME          hmfC0096
EXPNO                3
PROCNO               1
USER           majoral

F2 - Acquisition Parameters
Date_         20080429
Time             18.56
INSTRUM          spect
PROBHD   5 mm TXO 31P/1
PULPROG      ls_zg60dc
TD               65536
SOLVENT           DMSO
NS                 400
DS                   4
SWH          36496.352 Hz
FIDRES        0.556890 Hz
AQ           0.8978932 sec
RG             41285.1
DW              13.700 usec
DE               10.00 usec
TE               297.9 K
D1          1.00000000 sec
d11         0.03000000 sec
MCREST      0.00000000 sec
MCWRK       0.01500000 sec

======== CHANNEL f1 ========
NUC1               31P
P1                8.80 usec
PL1               0.00 dB
SFO1       121.4948510 MHz

======== CHANNEL f2 ========
CPDPRG2        waltz16
NUC2                1H
PCPD2            89.00 usec
PL2              -2.00 dB
PL12             17.00 dB
SFO2       300.1312005 MHz

F2 - Processing parameters
SI               65536
SF         121.4948510 MHz
WDW                 EM
SSB                  0
LB                1.00 Hz
GB                   0
PC                1.40
```

^{1}H NMR of dendrimer 3-G$_{1}$-Pd$_{12}$

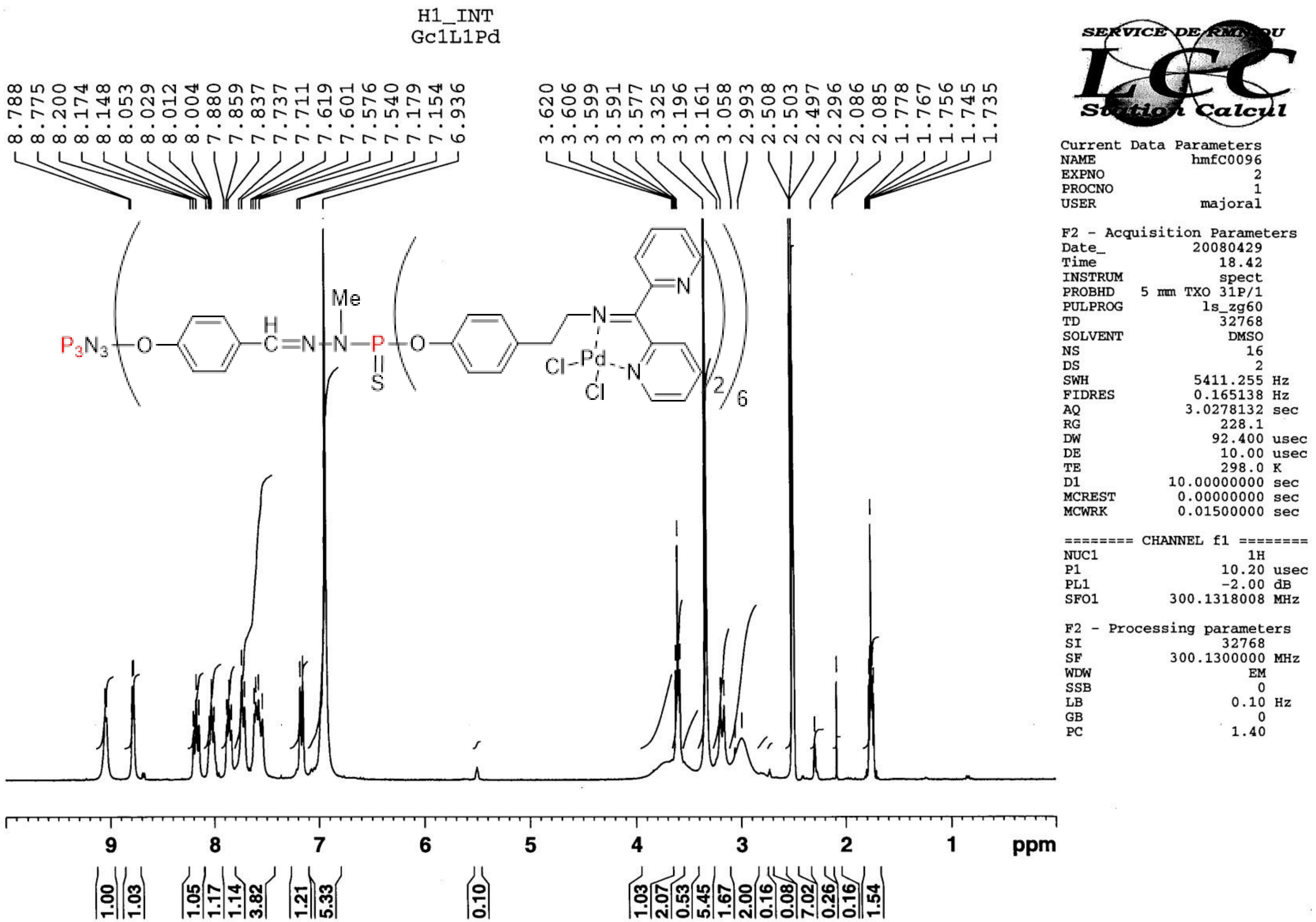
H1_INT
Gc1L1Pd
8.788 8.775 8.200 8.174 8.148 8.053 8.029 8.012 8.004 7.880 7.859 7.837 7.737 7.711 7.619 7.601 7.576 7.540 7.179 7.154 6.936
3.620 3.606 3.599 3.591 3.577 3.325 3.196 3.161 3.058 2.993 2.508 2.503 2.497 2.296 2.086 2.085 1.778 1.767 1.756 1.745 1.735
SERVICE DE RMN DU LCC Station Calcul
Current Data Parameters
NAME hmfC0096
EXPNO 2
PROCNO 1
USER majoral
F2 - Acquisition Parameters
Date_ 20080429
Time 18.42
INSTRUM spect
PROBHD 5 mm TXO 31P/1
PULPROG ls_zg60
TD 32768
SOLVENT DMSO
NS 16
DS 2
SWH 5411.255 Hz
FIDRES 0.165138 Hz
AQ 3.0278132 sec
RG 228.1
DW 92.400 usec
DE 10.00 usec
TE 298.0 K
D1 10.00000000 sec
MCREST 0.00000000 sec
MCWRK 0.01500000 sec
======== CHANNEL f1 ========
NUC1 1H
P1 10.20 usec
PL1 -2.00 dB
SFO1 300.1318008 MHz
F2 - Processing parameters
SI 32768
SF 300.1300000 MHz
WDW EM
SSB 0
LB 0.10 Hz
GB 0
PC 1.40
Me
H
P3N3
O
C
N
P
S
Cl
Pd
2
6
9 8 7 6 5 4 3 2 1 ppm
1.00 1.03 1.05 1.17 1.14 3.82 1.21 5.33 0.10 1.03 2.07 0.53 5.45 1.67 2.00 0.16 0.08 7.02 0.26 0.16 1.54

^{13}C {^{1}H} NMR of dendrimer 3-G_1-Pd_{12}

13

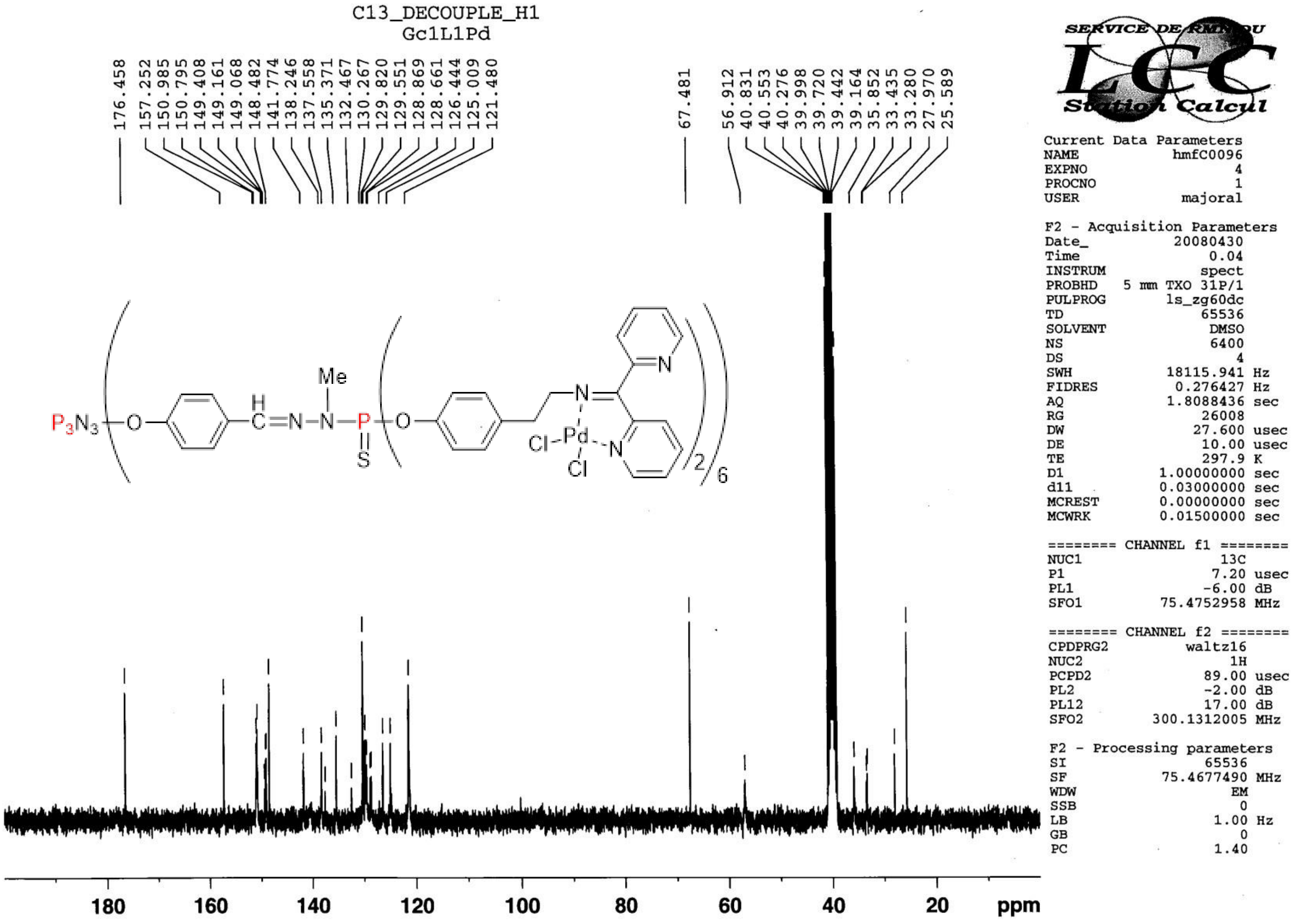
C13_DECOUPLE_H1
Gc1L1Pd
176.458 157.252 150.985 150.795 149.408 149.161 149.068 148.482 141.774 138.246 137.558 135.371 132.467 130.267 129.820 129.551 128.869 128.661 126.444 125.009 121.480
67.481
56.912 40.831 40.553 40.276 39.998 39.720 39.442 39.164 35.852 33.435 33.280 27.970 25.589
Current Data Parameters
NAME hmfC0096
EXPNO 4
PROCNO 1
USER majoral
F2 - Acquisition Parameters
Date_ 20080430
Time 0.04
INSTRUM spect
PROBHD 5 mm TXO 31P/1
PULPROG ls_zg60dc
TD 65536
SOLVENT DMSO
NS 6400
DS 4
SWH 18115.941 Hz
FIDRES 0.276427 Hz
AQ 1.8088436 sec
RG 26008
DW 27.600 usec
DE 10.00 usec
TE 297.9 K
D1 1.00000000 sec
d11 0.03000000 sec
MCREST 0.00000000 sec
MCWRK 0.01500000 sec
======== CHANNEL f1 ========
NUC1 13C
P1 7.20 usec
PL1 -6.00 dB
SFO1 75.4752958 MHz
======== CHANNEL f2 ========
CPDPRG2 waltz16
NUC2 1H
PCPD2 89.00 usec
PL2 -2.00 dB
PL12 17.00 dB
SFO2 300.1312005 MHz
F2 - Processing parameters
SI 65536
SF 75.4677490 MHz
WDW EM
SSB 0
LB 1.00 Hz
GB 0
PC 1.40
180 160 140 120 100 80 60 40 20 ppm

^{13}C JMOD NMR of dendrimer 3-G_1-Pd_{12}

13

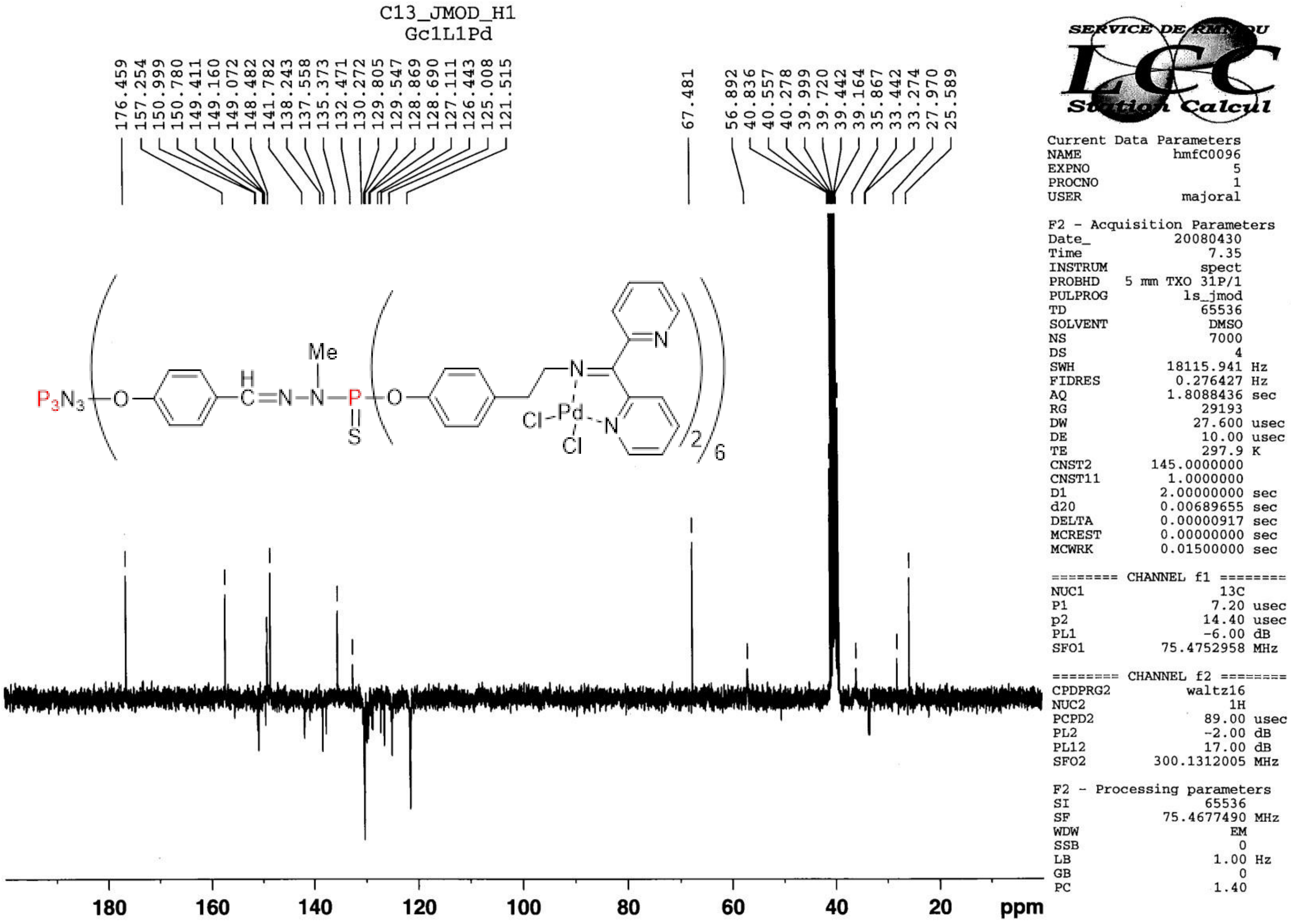

C13_JMOD_H1
Gc1L1Pd
176.459
157.254
150.999
150.780
149.411
149.160
149.072
148.482
141.782
138.243
137.558
135.373
132.471
130.272
129.805
129.547
128.869
128.690
127.111
126.443
125.008
121.515
67.481
56.892
40.836
40.557
40.278
39.999
39.720
39.442
39.164
35.867
33.442
33.274
27.970
25.589
SERVICE DE RMN DU
LCC
Station Calcul
Current Data Parameters
NAME hmfC0096
EXPNO 5
PROCNO 1
USER majoral
F2 - Acquisition Parameters
Date_ 20080430
Time 7.35
INSTRUM spect
PROBHD 5 mm TXO 31P/1
PULPROG ls_jmod
TD 65536
SOLVENT DMSO
NS 7000
DS 4
SWH 18115.941 Hz
FIDRES 0.276427 Hz
AQ 1.8088436 sec
RG 29193
DW 27.600 usec
DE 10.00 usec
TE 297.9 K
CNST2 145.0000000
CNST11 1.0000000
D1 2.00000000 sec
d20 0.00689655 sec
DELTA 0.00000917 sec
MCREST 0.00000000 sec
MCWRK 0.01500000 sec
======== CHANNEL f1 ========
NUC1 13C
P1 7.20 usec
p2 14.40 usec
PL1 -6.00 dB
SFO1 75.4752958 MHz
======== CHANNEL f2 ========
CPDPRG2 waltz16
NUC2 1H
PCPD2 89.00 usec
PL2 -2.00 dB
PL12 17.00 dB
SFO2 300.1312005 MHz
F2 - Processing parameters
SI 65536
SF 75.4677490 MHz
WDW EM
SSB 0
LB 1.00 Hz
GB 0
PC 1.40
180
160
140
120
100
80
60
40
20
ppm

^{13}C HMQC NMR of dendrimer 3-G$_1$-Pd$_{12}$

13

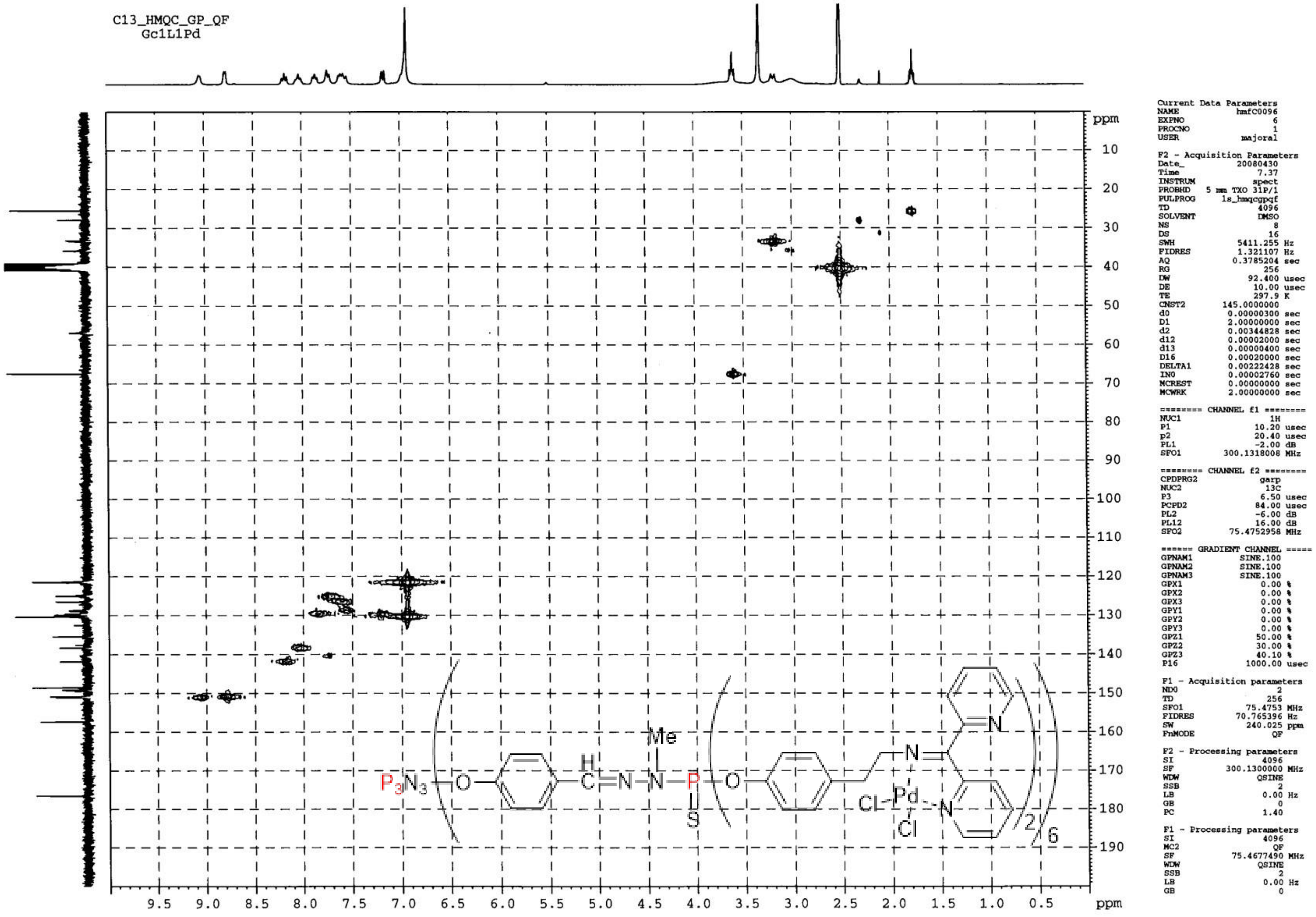
C13_HMQC_GP_QF
Gc1L1Pd
ppm
9.5 9.0 8.5 8.0 7.5 7.0 6.5 6.0 5.5 5.0 4.5 4.0 3.5 3.0 2.5 2.0 1.5 1.0 0.5
10 20 30 40 50 60 70 80 90 100 110 120 130 140 150 160 170 180 190
P3N3 O H C N N Me P S O N N Pd Cl Cl N 2 6
Current Data Parameters
NAME hmfC0096
EXPNO 6
PROCNO 1
USER majoral
F2 - Acquisition Parameters
Date_ 20080430
Time 7.37
INSTRUM spect
PROBHD 5 mm TXO 31P/1
PULPROG ls_hmqcgpqf
TD 4096
SOLVENT DMSO
NS 8
DS 16
SWH 5411.255 Hz
FIDRES 1.321107 Hz
AQ 0.3785204 sec
RG 256
DW 92.400 usec
DE 10.00 usec
TE 297.9 K
CNST2 145.0000000
d0 0.00000300 sec
D1 2.00000000 sec
d2 0.00344828 sec
d12 0.00002000 sec
d13 0.00000400 sec
D16 0.00020000 sec
DELTA1 0.00222428 sec
IN0 0.00002760 sec
MCREST 0.00000000 sec
MCWRK 2.00000000 sec
======== CHANNEL f1 ========
NUC1 1H
P1 10.20 usec
p2 20.40 usec
PL1 -2.00 dB
SFO1 300.1318008 MHz
======== CHANNEL f2 ========
CPDPRG2 garp
NUC2 13C
P3 6.50 usec
PCPD2 84.00 usec
PL2 -6.00 dB
PL12 16.00 dB
SFO2 75.4752958 MHz
====== GRADIENT CHANNEL =====
GPNAM1 SINE.100
GPNAM2 SINE.100
GPNAM3 SINE.100
GPX1 0.00 %
GPX2 0.00 %
GPX3 0.00 %
GPY1 0.00 %
GPY2 0.00 %
GPY3 0.00 %
GPZ1 50.00 %
GPZ2 30.00 %
GPZ3 40.10 %
P16 1000.00 usec
F1 - Acquisition parameters
ND0 2
TD 256
SFO1 75.4753 MHz
FIDRES 70.765396 Hz
SW 240.025 ppm
FnMODE QF
F2 - Processing parameters
SI 4096
SF 300.1300000 MHz
WDW QSINE
SSB 2
LB 0.00 Hz
GB 0
PC 1.40
F1 - Processing parameters
SI 4096
MC2 QF
SF 75.4677490 MHz
WDW QSINE
SSB 2
LB 0.00 Hz
GB 0

{^{1}H} NMR of dendrimer 3

^{31}P **-G$_2$-Pd$_{24}$**

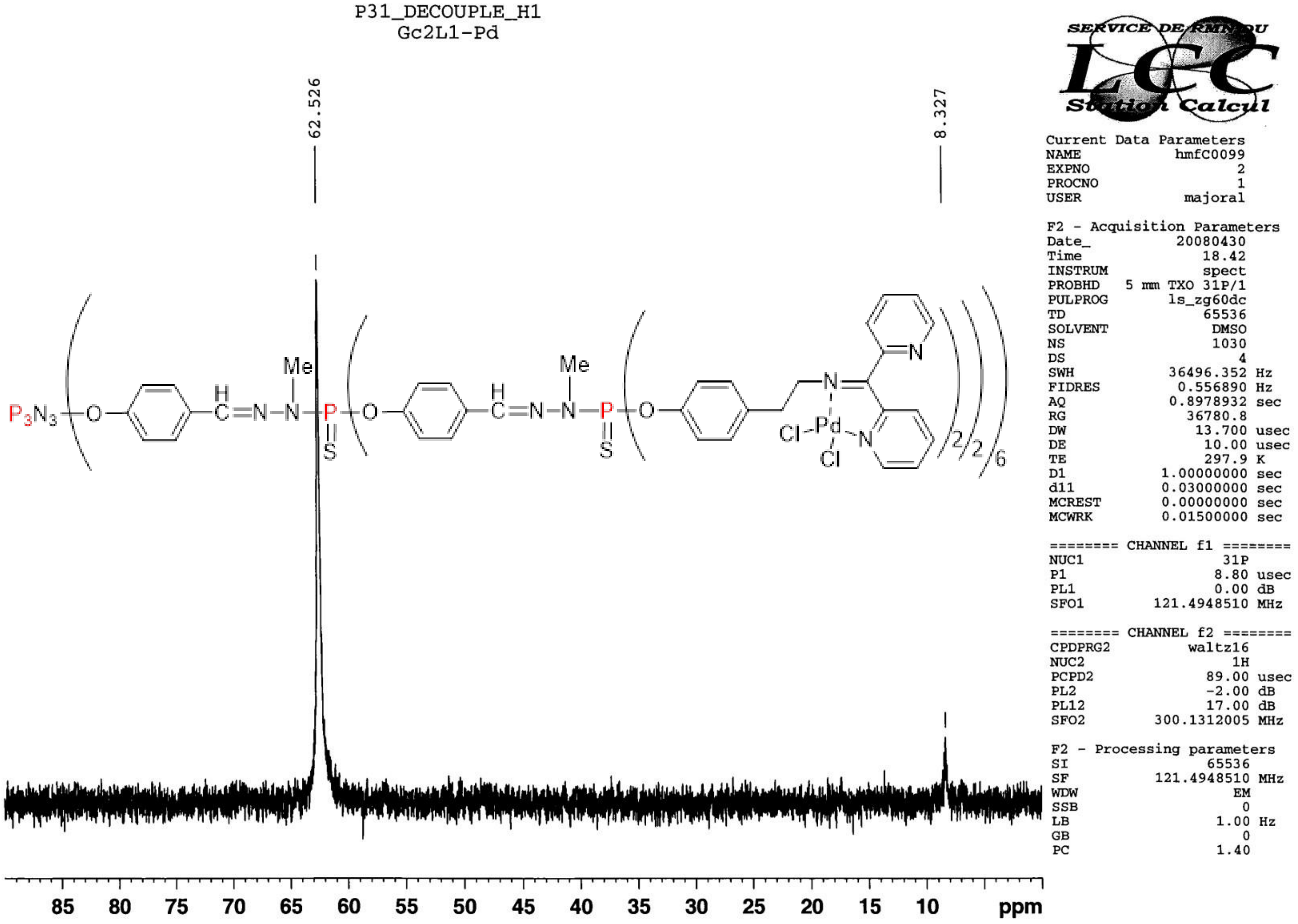

^{1}H NMR of dendrimer 3-G_2-Pd_{24}

{1H} NMR of dendrimer 3

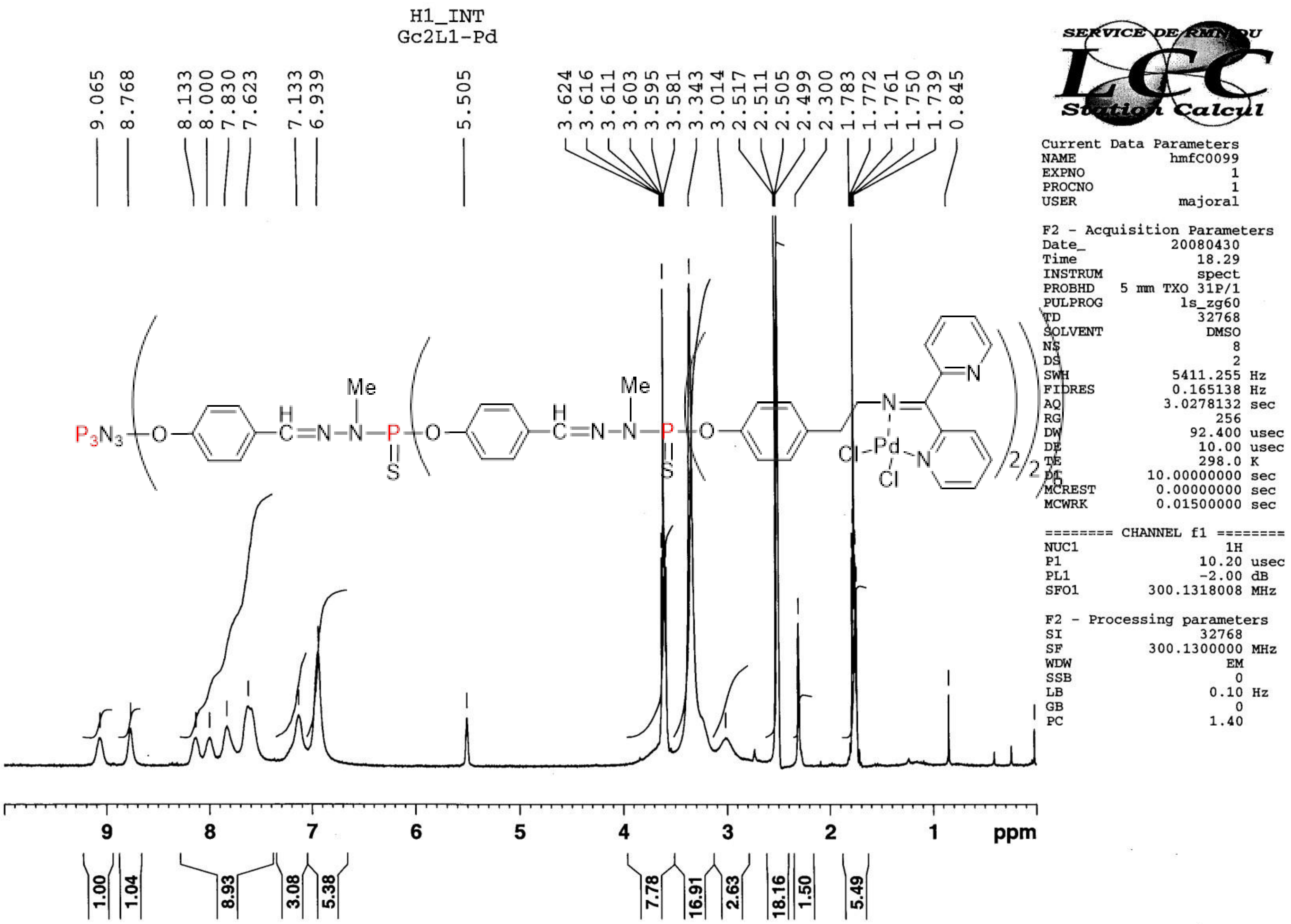

13C -G2-Pd24

{^{1}H} NMR of dendrimer 3

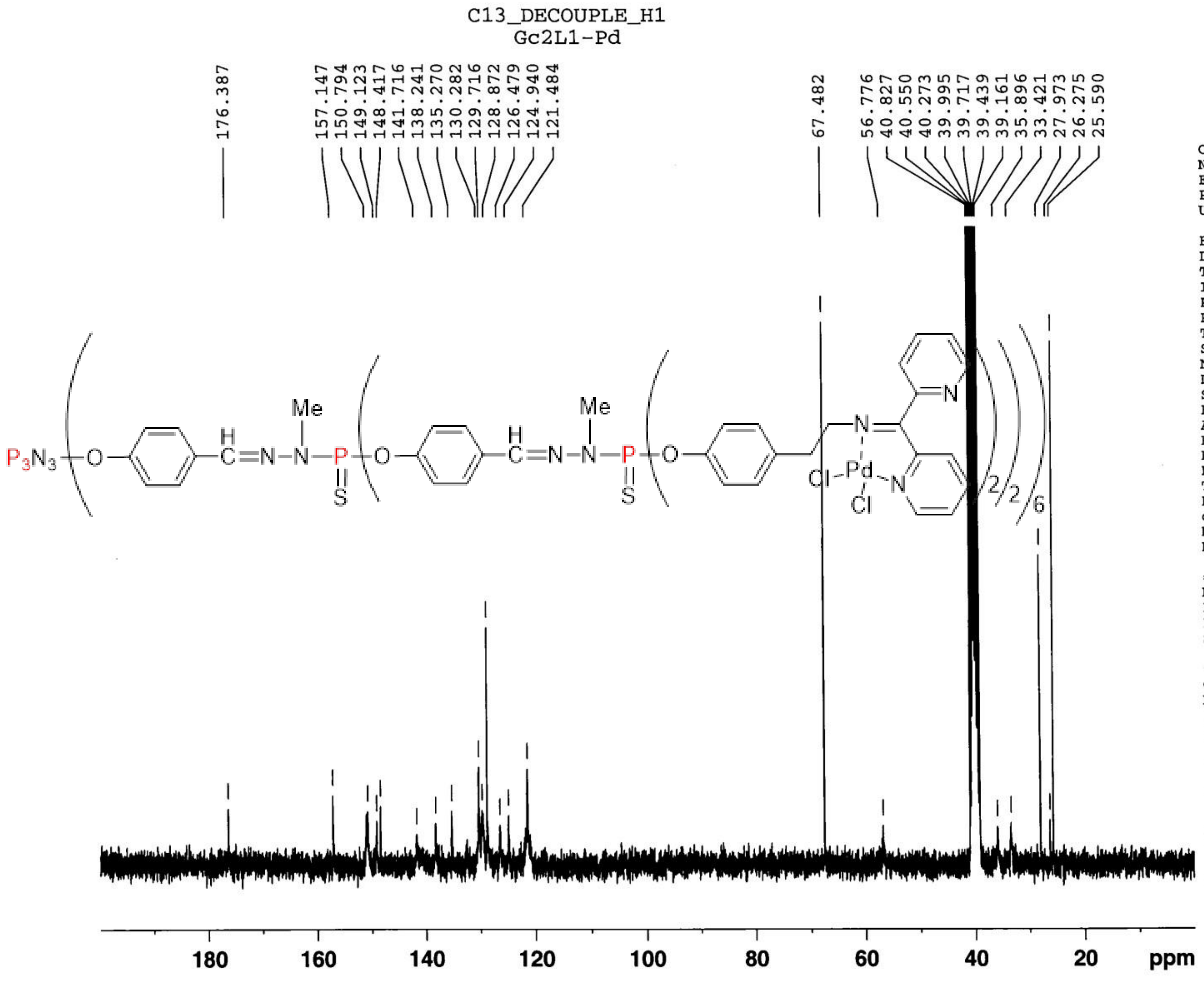

Current Data Parameters
NAME hmfC0099
EXPNO 3
PROCNO 1
USER majoral

F2 - Acquisition Parameters
Date_ 20080501
Time 10.37
INSTRUM spect
PROBHD 5 mm TXO 31P/1
PULPROG ls_zg60dc
TD 65536
SOLVENT DMSO
NS 19200
DS 4
SWH 18115.941 Hz
FIDRES 0.276427 Hz
AQ 1.8088436 sec
RG 29193
DW 27.600 usec
DE 10.00 usec
TE 297.9 K
D1 1.00000000 sec
d11 0.03000000 sec
MCREST 0.00000000 sec
MCWRK 0.01500000 sec

======== CHANNEL f1 ========
NUC1 13C
P1 7.20 usec
PL1 -6.00 dB
SFO1 75.4752958 MHz

======== CHANNEL f2 ========
CPDPRG2 waltz16
NUC2 1H
PCPD2 89.00 usec
PL2 -2.00 dB
PL12 17.00 dB
SFO2 300.1312005 MHz

F2 - Processing parameters
SI 65536
SF 75.4677490 MHz
WDW EM
SSB 0
LB 1.00 Hz
GB 0
PC 1.40

^{13}C JMOD NMR of dendrimer 3-G_2-Pd_{24}

{^{1}H} NMR of dendrimer 3

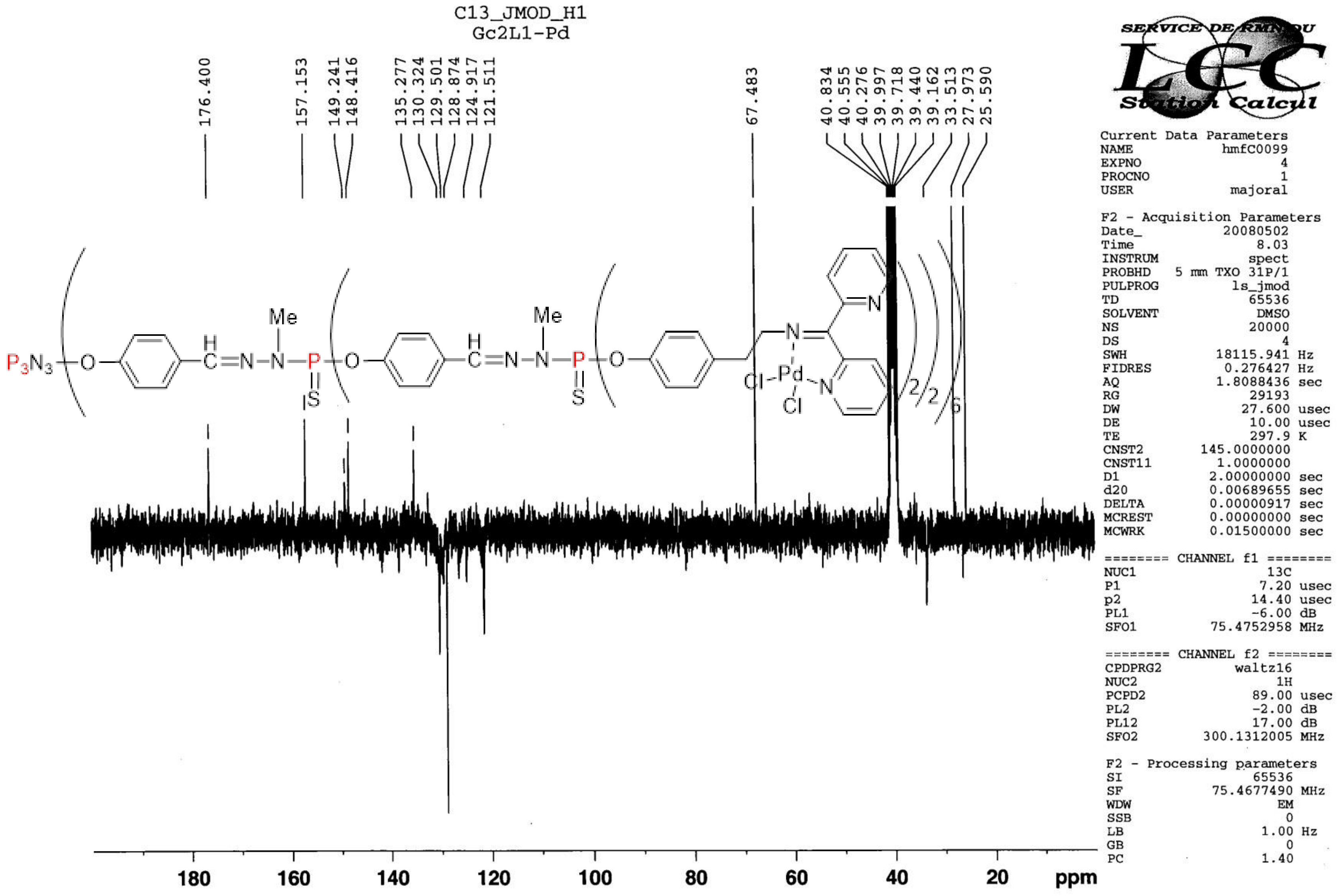

^{31}P **-G$_3$-Pd$_{48}$**

{¹H} NMR of dendrimer 3

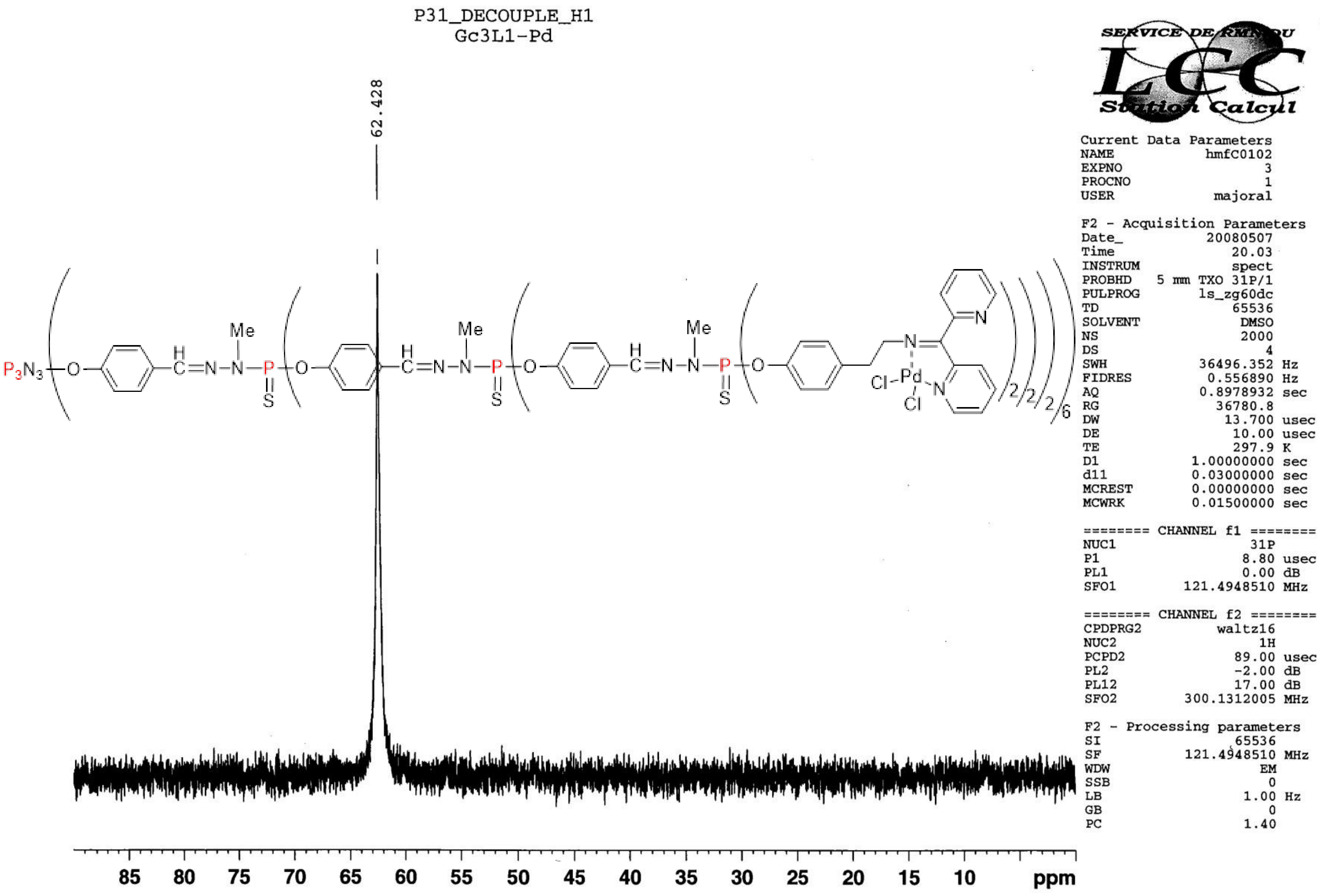

^{1}H NMR of dendrimer 3-G$_3$-Pd$_{48}$

{^{1}H} NMR of dendrimer 3

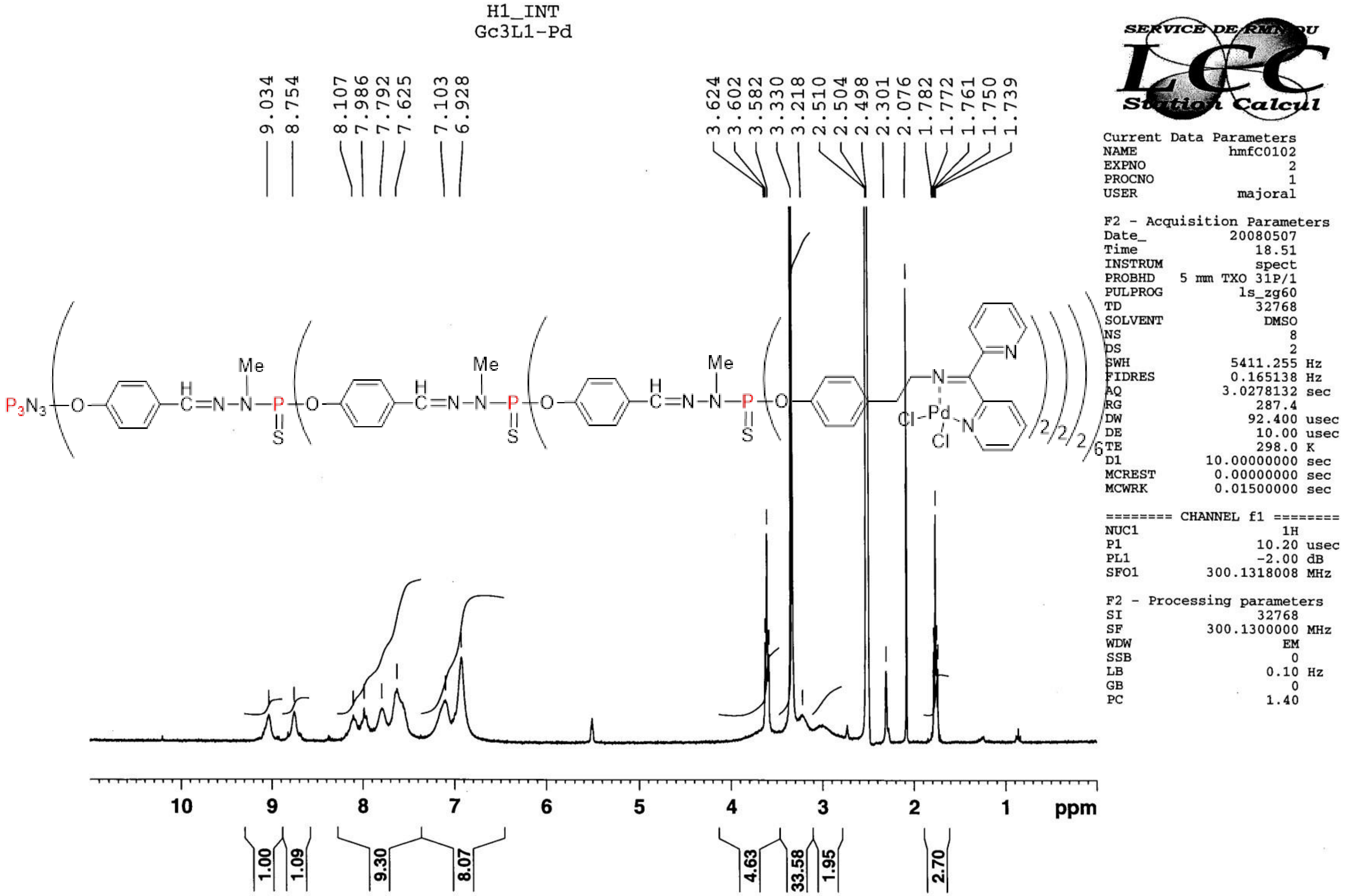

$^{13}C\{^1H\}$ NMR of dendrimer 3-G_3-Pd_{48}

{^{1}H} NMR of

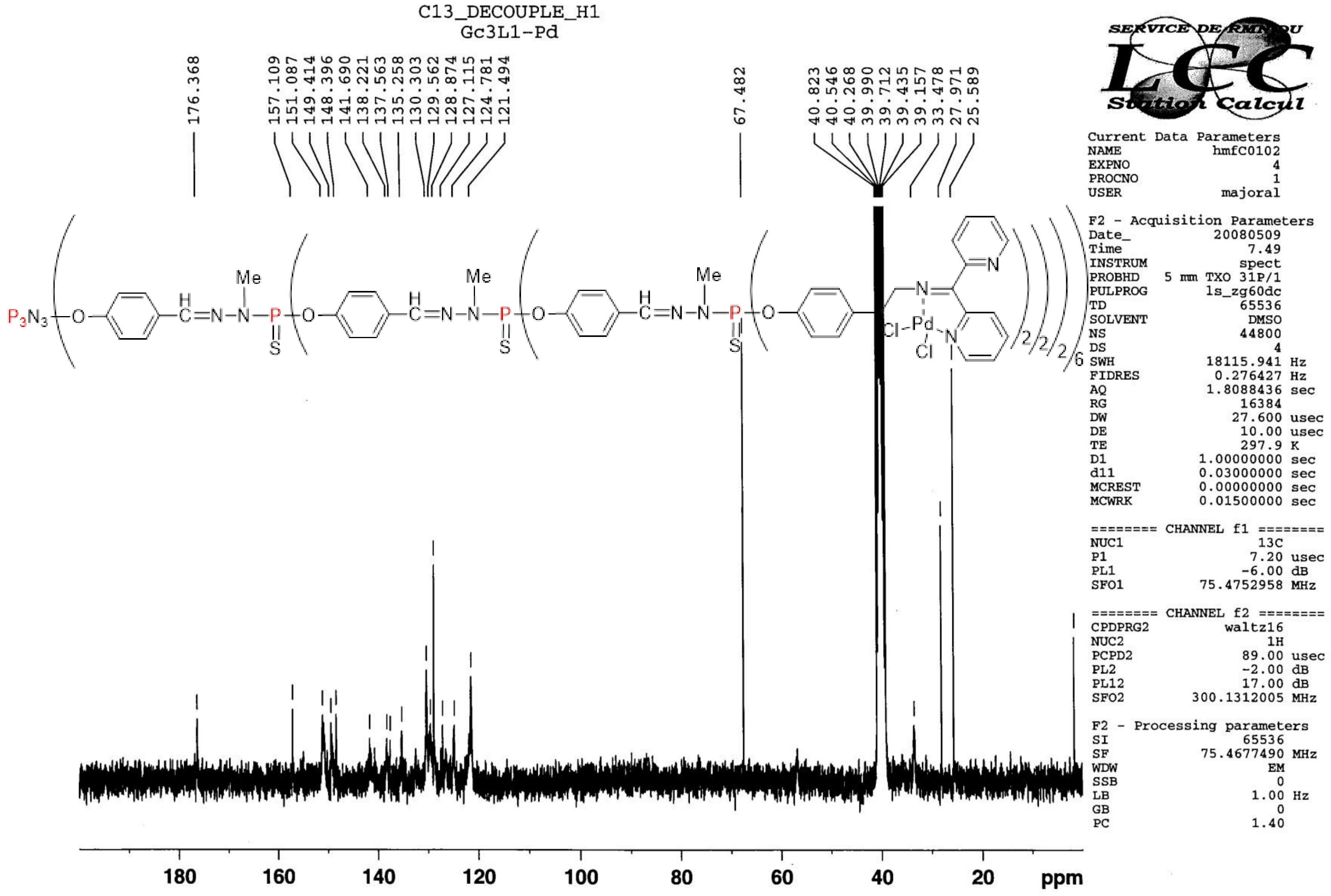

C13_DECOUPLE_H1
Gc3L1-Pd
176.368
157.109
151.087
149.414
148.396
141.690
138.221
137.563
135.258
130.303
129.562
128.874
127.115
124.781
121.494
67.482
40.823
40.546
40.268
39.990
39.712
39.435
39.157
33.478
27.971
25.589
Current Data Parameters
NAME hmfC0102
EXPNO 4
PROCNO 1
USER majoral
F2 - Acquisition Parameters
Date_ 20080509
Time 7.49
INSTRUM spect
PROBHD 5 mm TXO 31P/1
PULPROG ls_zg60dc
TD 65536
SOLVENT DMSO
NS 44800
DS 4
SWH 18115.941 Hz
FIDRES 0.276427 Hz
AQ 1.8088436 sec
RG 16384
DW 27.600 usec
DE 10.00 usec
TE 297.9 K
D1 1.00000000 sec
d11 0.03000000 sec
MCREST 0.00000000 sec
MCWRK 0.01500000 sec
======== CHANNEL f1 ========
NUC1 13C
P1 7.20 usec
PL1 -6.00 dB
SFO1 75.4752958 MHz
======== CHANNEL f2 ========
CPDPRG2 waltz16
NUC2 1H
PCPD2 89.00 usec
PL2 -2.00 dB
PL12 17.00 dB
SFO2 300.1312005 MHz
F2 - Processing parameters
SI 65536
SF 75.4677490 MHz
WDW EM
SSB 0
LB 1.00 Hz
GB 0
PC 1.40
180
160
140
120
100
80
60
40
20
ppm
